# Update on the Inactivation Procedures for the Vaccine Development Prospects of a New Highly Virulent RGNNV Isolate

**DOI:** 10.3390/vaccines9121441

**Published:** 2021-12-07

**Authors:** Alberto Falco, Melissa Bello-Perez, Rocío Díaz-Puertas, Matthew Mold, Mikolaj Adamek

**Affiliations:** 1 Institute of Research, Development and Innovation in Healthcare Biotechnology in Elche (IDiBE), Miguel Hernández University, 03202 Elche, Spain; melissa.bello@alu.umh.es (M.B.-P.); rocio.diaz01@goumh.umh.es (R.D.-P.); 2 The Birchall Centre, Lennard-Jones Laboratories, Keele University, Staffordshire ST5 5BG, UK; matthew.j.mold@gmail.com; 3 Fish Disease Research Unit, Institute for Parasitology, University of Veterinary Medicine, 30559 Hannover, Germany; mikolaj.adamek@tiho-hannover.de

**Keywords:** viral nervous necrosis, betanodavirus, nervous necrosis virus, autogenous vaccine, virus inactivation, virus-inactivated vaccine

## Abstract

Viral nervous necrosis (VNN) caused by the nervous necrosis virus (NNV) affects a broad range of primarily marine fish species, with mass mortality rates often seen among larvae and juveniles. Its genetic diversification may hinder the effective implementation of preventive measures such as vaccines. The present study describes different inactivation procedures for developing an inactivated vaccine against a new NNV isolate confirmed to possess deadly effects upon the European seabass (*Dicentrarchus labrax*), an important Mediterranean farmed fish species that is highly susceptible to this disease. First, an NNV isolate from seabass adults diagnosed with VNN was rescued and the sequences of its two genome segments (RNA1 and RNA2) were classified into the red-spotted grouper NNV (RGNNV) genotype, closely clustering to the highly pathogenic 283.2009 isolate. The testing of different inactivation procedures revealed that the virus particles of this isolate showed a marked resistance to heat (for at least 60 °C for 120 min with and without 1% BSA) but that they were fully inactivated by 3 mJ/cm^2^ UV-C irradiation and 24 h 0.2% formalin treatment, which stood out as promising NNV-inactivation procedures for potential vaccine candidates. Therefore, these procedures are feasible, effective, and rapid response strategies for VNN control in aquaculture.

## 1. Introduction

The scarcity of alternative therapeutics against viral infections that are as efficient as vaccines makes them essential in such areas as animal care [1]. Current vaccines can be classified into four main groups: whole-pathogen (traditional), recombinant, protein-based, and nucleic acid vaccines [2]. Most veterinary vaccines in use today fall into the former category because of their cost-effectiveness and/or the insufficient knowledge of many pathogens at the molecular level [3,4,5,6,7]. Whole-pathogen vaccines consist of live-attenuated or weakened microorganisms, which usually yield improved immunological assets versus killed or inactivated microorganisms. In comparison, the latter presents substantial biosafety and handling advantages [2] and thus a higher market acceptance in the animal production industry, including aquaculture [3,4,5,6,7].

The overall production procedure used for all virus-inactivated vaccines consists of harvesting large amounts of viral particles (often purified and concentrated) and their subsequent inactivation by chemical and/or physical methods [8]. In this sense, several inactivation agents have been reported to be successful for this purpose, including many examples for fish viral pathogens. While formalin predominates in this respect [3,4,6,9,10,11], β-propiolactone [11,12], binary ethyleneimine (BEI) [9,13], heat [10,14,15], and ultraviolet (UV) irradiation [10,16] have additionally been used. Nevertheless, to the best of our knowledge, only formalin has been used for the inactivation of licensed fish viral vaccines [3,4,6].

The most critical factor in developing these vaccines is to ascertain, for a given inactivating agent, the specificities at which it achieves the complete inactivation of a particular pathogen without compromising its antigenicity [3,8]. Indeed, maintaining the integrity of the antigen after inactivation is required by the host immune system to facilitate its recognition ability and to avoid potential detrimental effects for the host in following encounters with the pathogen [3,8]. Inactivated antigens usually retain weaker immunogenicity, thus necessitating adjuvants to induce more robust and/or longer-lasting immunity, that may occasionally produce toxic reactions in the host [6,8,17]. In overcoming these drawbacks, inactivated vaccines offer (i) biosafety rewards, such as the impossibility of initiating new outbreaks or a high biodegradability within the environment and in vaccinated organisms, and (ii) a high suitability for the rapid generation of tailor-made or autogenous vaccines to tackle problematic local pathogen isolates primarily affecting animal farming [6,18]. One promising candidate, for example, may be the betanodavirus (*Nodaviridae* family) in the fish aquaculture sector.

*Nodaviridae* comprises ~30 nm non-enveloped icosahedral viruses with genomes consisting of two positive-sense single-stranded RNA segments—namely, RNA1 and RNA2 [19,20]. RNA1 is about 3.1 kilobases (kb) in length and encodes for the RNA-dependent RNA polymerase (RdRP) and the subgenomic transcript RNA3 encoding for the non-structural B2-like protein, which potentially binds double-stranded RNA, preventing its recognition and degradation by host immune defenses [19,20,21,22]. RNA2 is about 1.3 kb and encodes for the capsid protein (CP), which assembles into virus particles using approximately 180 units with a triangulation number of 3 [19,20,23].

Nodaviruses are classified based on their hosts into the genera *Alphanodavirus* (insects) and *Betanodavirus* (fish), as well as a suggested additional one termed *Gammavirus* (prawns) [20,24]. Based on its histopathology, the betanodavirus, also known as nervous necrosis virus (NNV), cause a fish diseases termed viral nervous necrosis (VNN) [25], encephalomyelitis, or vacuolating encephalopathy and retinopathy (VER), among others [19,26]. In over 120 species spanning 30 families from 11 different orders reported to be affected by NNV worldwide so far, most cases are associated with warm water marine fish, particularly groupers, seabass, and flatfish [19,26,27]. In addition, the horizontal and, especially, vertical transmission abilities of NNV [28,29] highly contribute to the expansion and severity of occurring outbreaks [27,30], since fish larvae and juveniles are usually more susceptible to this infection, often leading to mass mortality outcomes [31,32,33]. In fact, since the first reported NNV outbreaks in the mid-late 1980s [25,34], the number of identified severe NNV outbreaks has increased, diverged, and expanded continuously [19,26,27], resulting in a severe constraint to the aquaculture industry globally [19,27,35]. Facing this scenario, vaccination postulates a promising control strategy, since survivors of NNV infections show higher immunoglobulin expression [36] and the presence of neutralizing antibodies and resistance to re-infection [37]. Furthermore, numerous different experimental vaccines have proven high protection levels against VNN [19,27].

However, the genetic diversification of the virus may hinder the effective implementation of vaccines. Indeed, the comparison of the highly variable region of the gene encoding for the CP, corresponding to the so-called T4 region located at its C-terminal half [38], has allowed the classification of betanodaviruses into one of four genotypes [39]: barfin flounder NNV (BFNNV), red-spotted grouper NNV (RGNNV), striped jack NNV (SJNNV), and tiger puffer NNV (TPNNV). Subsequent phylogenetic studies, including partial and full sequences of both genome segments, have confirmed this organization and reported the natural occurrence of multiple reassortants, particularly between RGNNV and SJNNV [35,40,41], increasing the complexity of their epidemiology. Moreover, and also unfortunately for vaccine implementation prospects, the four NNV genotypes fall into three different serotypes (A, B, and C, corresponding to SJNNV, TPNNV, and BFNNV/RGNNV, respectively) [42], and no cross-neutralization has been found among SJNNV and BFNNV/RGNNV serotypes in different experiments using SJNNV-polyclonal or RGNNV-polyclonal/monoclonal antibodies [30,43].

Given these drawbacks, it is not surprising that, to the best of our knowledge, no commercial vaccines spanning all NNV genotypes are available yet, but just two inactivated (formalin-killed) anti-RGNNV vaccines for sea bass in the Mediterranean market—i.e., Icthiovac^®^ VNN (Laboratorios Hipra SA, Amer, Spain) and Alpha ject micro^®^ 1Noda (Pharmaq AS, Oslo, Norway). Thus, the improvement of the development of inactivated vaccines against local VNN incidences—i.e., autogenous vaccines—has arisen as a topic of increasing interest, supported by the high efficiency of the antibody response elicited and the protection conferred after vaccination reported for several experimental NNV-inactivated vaccines [44,45,46].

In the present study, an NNV isolate from a seabass exhibiting typical VNN pathological symptoms was rescued, propagated, identified, and characterized for this task. Subsequently, the efficiency of different physical and chemical virus inactivation procedures, including ultraviolet radiation, heat, and formalin treatments, combining inputs from different related works on NNV, was determined in vitro by analyzing the viral replication of treated samples by quantitative polymerase chain reaction (qPCR) in an attempt to increase the sensing of infectious virus in comparison to previous studies in the field. In parallel, the inoculated cells were also analyzed by qPCR regarding the potential induction of the interferon (IFN) system in response to virus replication or residual viral components. Altogether, the present work provides a summary of procedures used to initially develop autogenous vaccines for the aquaculture sector, using NNV as model pathogen given its growing incidence and genetic diversification, as well as the limited number and reach of commercial vaccines.

## 2. Materials and Methods

### 2.1. Cell Lines and Virus

The cell line from fins of gilt-head seabream SAF-1 was purchased from the European Collection of Authenticated Cell Cultures (ECACC, Porton Down, UK, ref. no. 00122301) [47]. The E11 clone of the persistently C-type retrovirus (SnRV) -infected cell line SSN-1 derived from striped snakehead, *Channa striatus* [48,49], was also purchased from ECACC (ref. no. 01110916). Both cell lines were grown at 25 °C in a 5% CO_2_ atmosphere in Leibovitz L-15 medium supplemented with 10% foetal bovine serum (FBS), 2 mM glutamine, 100 U/mL penicillin, and 0.1 mg/mL streptomycin (all products from Sigma, St. Louis, MO, USA).

The 378/I02 NNV isolate from the RGNNV genotype (GenBank ref. nos. RNA1/RNA2: JX290515/JX290517) was used for optimization and control testing. Virus stocks from all NNV isolates were obtained by propagating in ~80% confluent E11 cell monolayers at 25 °C using the previously described cell growth media with 2% instead of 10% FBS (infection media). Thus, when the cytopathogenic effect (CPE) was extensive (~60%), the infected cell cultures were subjected to a freeze/thaw cycle (−20/25 °C), harvested, and clarified by centrifugation at 4000× *g* for 10 min and, finally, aliquoted and stored at −80 °C until they could be used. Virus stocks were titrated (50% cell-culture infective dose (CCID_50_) per mL) using the Reed–Muench method [50].

### 2.2. Microscopy

A DMI 3000B-inverted microscope coupled with an EL6000 compact light source and a DFC3000G digital camera (Leica, Bensheim, Germany) was used to visualize and capture photographs of cell monolayers.

### 2.3. Isolation of NNV

Three seabass individuals of ~25 cm body length exhibiting typical pathological symptoms of NNV infection from a fish farm in the Canary Islands (Spain) in 2015 were sacrificed and their brain tissue was collected and stored at −80 °C until it could be used.

The viral isolates were rescued from these tissues using E11 cells in 25 cm^2^ cell culture flasks according to the methodology described by the OIE [51]. Briefly, after three blind culture passages (~4 days per passage), cell monolayers started showing evident CPE. When CPE was extensive, virus stocks were obtained as described above. The identification of the new viral isolates was carried out by sequencing the putative NNV genomic fragments amplified by reverse transcriptase-polymerase chain reaction (RT-PCR) (see Section 2.5 for further details in this regard). The new isolate was internally labeled as V1059.

### 2.4. RNA Isolation and cDNA Synthesis

For the processing of samples for conventional PCR and qPCR, total RNA was isolated from cultured cells and organ tissues using the EZNA^®^ HP Total RNA and EZNA^®^ HP Tissue RNA kits from Omega Bio-tek (Norcross, GA, USA), respectively. The DNase treatment was performed with the Turbo DNA-free™ kit (Ambion Inc., Austin, TX, USA). Each cultured cell sample was obtained by pooling four of the 96 wells in the plates. RNA concentrations were estimated with a Nanodrop 1000 spectrophotometer (Thermo Fisher Scientific, Waltham, MA, USA). Isolated RNA samples were stored at −80 °C until they could be used. Then, for the synthesis of cDNA, Moloney murine leukemia virus (M-MuLV) reverse transcriptase (Invitrogen, Carlsbad, CA, USA) and 1 μg of isolated RNA from each sample were used as previously described [52].

### 2.5. PCR and Sequencing

All conventional PCR amplification reactions were carried out in a GeneAmp^®^ PCR System 2700 cycler (Applied Biosystems, Foster City, CA, USA) using 0.5 μL dNTP mix (10 mM each), 0.125 μL Taq DNA polymerase, 2.5 μL Taq 10× buffer (all from Invitrogen), 1 μL of forward and reverse primers (20 μM) (Sigma), and 2 μL of cDNA in a final volume of 25 μL.

The specific primers used for each NNV genome fragment were designed targeting highly conserved regions of the RGNNV genotype, as the most predominant worldwide and in the particular geographical area where the outbreak was located [35]. The multiple sequence alignments used to identify such regions were created using the bioinformatic tool Clustal Omega hosted at the server of the European Molecular Biology Laboratory of the European Bioinformatics Institute (EMBL-EBI, Hinxton, UK, http://www.ebi.ac.uk/Tools/msa/clustalo/) (accessed on 16 May 2021) [53]. For this task, the RGNNV complete sequences sharing less than 99% identity that were available at the GenBank database were retrieved (National Center for Biotechnology Information, NCBI, National Institutes of Health, NIH, Bethesda, MD, USA) (Tables S1 and S2 for RNA1 and RNA2 sequences, respectively) (accessed on 16 May 2021). Thus, the respective forward and reverse primer sequences chosen were 5′-CCGATATCACGATGAGTTCAC-3′ and 5′-CTTAGCCCAGCCAATGTC-3′ for RNA1 (*rdrp* gene) and 5′-AATGGTACGCAAAGGTGA-3′ and 5′-GCACTAGGGAACCGGAT-3′ for RNA2 (*cp* gene). These alignments and the locations of the primer sequences within them are shown in Figures S1 and S2 for RNA1 and RNA2, respectively. A search using the Basic Local Alignment Search Tool (BLAST, NCBI) with the primer sequences showed no genomic cross-reactivity with other virus families.

The amplification conditions consisted of a denaturing step at 94 °C for 1 min followed by 35 cycles of 95 °C for 30 s, 58 °C for 30 s, and 72 °C for 1 min, followed by a final extension step of 72 °C for 7 min. PCR products (5 μL) were visualized on a 1.5% agarose gel stained with SYBR Safe^®^ (Invitrogen). The 1 kb DNA ladder from Promega (Madison, WI, USA) was used as a size marker. Finally, the bands obtained were excised and purified using the ‘‘Isolate PCR and Gel’’ kit (Bioline, London, UK), before being commercially sequenced (Sistemas Genómicos S.L., Valencia, Spain) (Figure S3).

### 2.6. Phylogenetic Analysis

To identify the amplified viral nucleotide fragments of each genome segment, they were individually used to screen the database Nucleotide collection (nr/nt) with the Basic Local Alignment Search Tool (BLAST). BLASTn was preferentially used under default settings for searching highly similar sequences (megablast). The resulting records of sequences displaying the highest e-values with more than 95% identity in 100% alignment coverage are listed in Tables S3 and S4 for the RNA1 and RNA2 sequences, respectively (last accessed: 16 May 2021).

The Molecular Evolutionary Genetics Analysis software version 10.2.5 (MEGA X) was used for the phylogenetic analysis of the obtained sequences [54]. A multiple sequence alignment obtained with Clustal Omega was used as previously described and also included the GenBank complete sequences of all NNV genotypes. It included the reference genotype strains, which shared less than 99% identity (Tables S1 and S2 for RNA1 and RNA2 sequences, respectively) (last accessed: 16 May 2021). The evolutionary history was inferred using the Neighbor-Joining method, and its evolutionary distances were computed using the Maximum Composite Likelihood method. These data were represented in a bootstrap consensus tree from 500 replicate analysis, in which branches corresponding to partitions reproduced in less than 50% bootstrap replicates were collapsed.

### 2.7. NNV Inactivation

In this study, both physical and chemical virus inactivation procedures were tested. All treatments were performed in triplicate, and the final volume of each replicate was 100 μL, corresponding to 50 μL of the virus solution at 2 × 10^5^ CCID_50_/mL in infection media and 50 μL of the inactivating reagents in phosphate-buffered saline (PBS) or just PBS.

Based on previous studies [16,55], the heat inactivation method consisted of heating the virus samples at 60 °C in a water bath for either 30 or 120 min. The addition of 1% bovine serum albumin (BSA, Sigma) as an interfering protein source was also tested. Non-heated control samples with and without NNV were performed the same way but incubated at 25 °C.

For the UV inactivation, NNV samples distributed in 96-well plates were exposed uncovered to different doses (J/cm^2^) of 254 nm UV-C irradiation using a Biolink™ BLX 254 apparatus (Vilber Lourmat, France) emitting at a 5 mJ/s radiation rate, in a similar manner to that described by other authors [16,55]. Control samples (with and without NNV) were manipulated the same way but not irradiated.

Regarding the chemical inactivation, formalin was used as a chemical inactivating agent, since it has been widely studied [44,45,46,55] and already used commercially for this purpose, as mentioned in the introductory section. For these assays, NNV was incubated with different dilutions of the 37% commercial formalin solution, formalin (Sigma), at 25 °C. At the end of each incubation time, residual free formalin was neutralized for 10 min with sodium bisulfite (NaHSO_3_, Sigma) at double the formalin concentration used. The control samples (with and without NNV) did not contain formalin.

After inactivation, all reaction mixtures were topped up to 500 μL with infection media. In order to restore neutral pH, they were supplemented with final HEPES concentrations of 4 mM in 1% BSA reactions and 4, 6, and 10 mM in 0.05, 0.1, and 0.2% formalin reactions, respectively. Finally, each reconstituted reaction mixture was incubated with SAF-1 cell monolayers grown in 24-well plates for 24 h at 25 °C. Infection levels were then determined by qPCR and validated using the Reed–Muench method when complete inactivation was detected.

### 2.8. qPCR

qPCR was performed as described previously [56]. For each sample, the number of transcripts of each target gene was normalized to the corresponding one of the endogenous reference gene utilizing a Livak and Schmittgen’s method modification [57] with the formula 2^Ct ref.−Ct target^. The elongation factor 1α (*ef1a*) was used as an endogenous reference gene. The in vitro experiments included the analysis of transcripts of the myxovirus (influenza) resistance protein (*mx*), a reporter gene of the induction of the interferon response and, therefore, also of viral infection [58,59]. The replication of NNV was determined by analyzing the expression of both *rdrp* and *cp* genes with specific primers designed within highly conserved regions for the RGNNV genotype, while also considering the sequences of the PCR products amplified in this study. Figures S1 and S2 show the locations of RNA1 and RNA2, respectively. All the primer sequences used for these quantitative studies are listed in Table 1.

### 2.9. Statistical Analysis and Graphics

Transcript expression data are shown as mean and standard deviation (SD) (*n* = 3), and the corresponding applied statistical methods are stated together with the analyzed data in the results section. Significant changes are indicated as: * (*p* < 0.05), ** (*p* < 0.01) and *** (*p* < 0.001). Prism v7 (Graphpad software, La Jolla, CA, USA) was used for creating the graphs and the statistical analysis.

## 3. Results and Discussion

### 3.1. NNV Isolation and Identification

**Figure 1:**
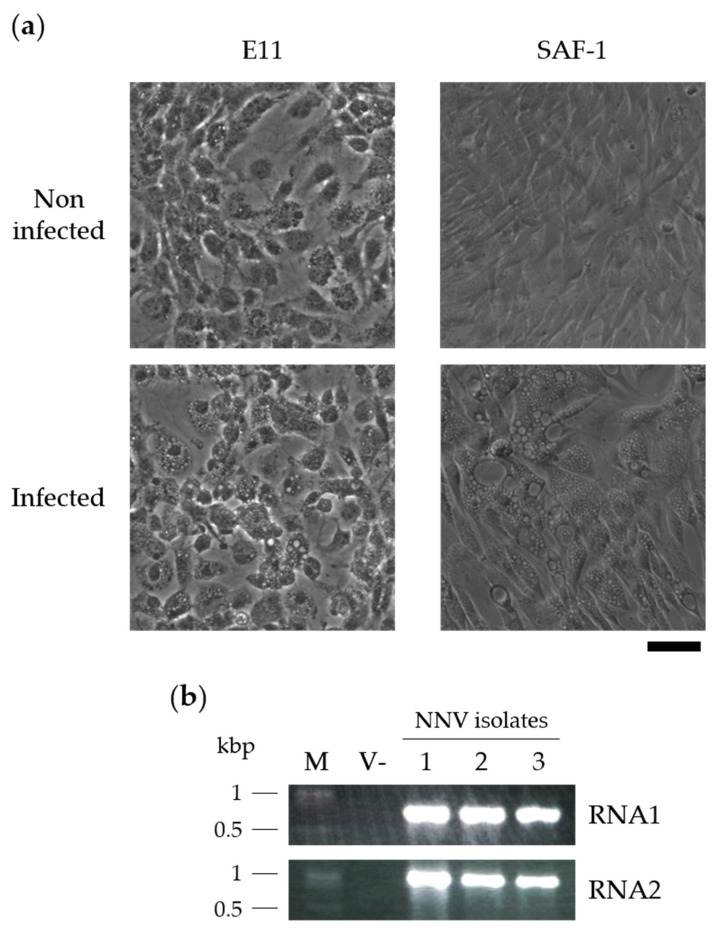
In vitro detection of NNV infection from rescued isolates. (**a**) Light microscopy of E11 and SAF-1 monolayers either inoculated or not inoculated with experimental isolates. Scale bar: 100 µm. (**b**) RT-PCR analysis of NNV RNA1 and RNA2 expression in E11 cells incubated with experimental isolates. PCR products were separated in a 1.5% agarose gel and stained with SYBR Safe^®^. M, DNA marker; V-, control cells; 1–3, cells inoculated with experimental isolates 1–3.

All brain tissue samples, which were obtained from the three seabass individuals showing typical VNN pathological symptoms, induced classically in vitro NNV CPE. Massive cellular vacuolization was observed in both E11 and SAF-1 cells (Figure 1a). Parallel in vitro assays confirmed NNV as the causative agent of the mentioned symptoms/effects, since single PCR products with expected lengths were amplified with specific primers for genes from both NNV genome fragments (~776 bp for *rdrp* in RNA1 and ~1000 bp for *cp* in RNA2) (Figure 1b). In this sense, the same PCR results were obtained when analyzing the original brain tissues for these genes (data not shown). Likewise, the reinfection of healthy seabass fry by the intraperitoneal injection of 10^4^ CCID_50_/fish reproduced the disease with its typical symptoms in these animals from the 7th day post infection, in which the presence of NNV was also detected in brain tissue as described before.

**Figure 2:**
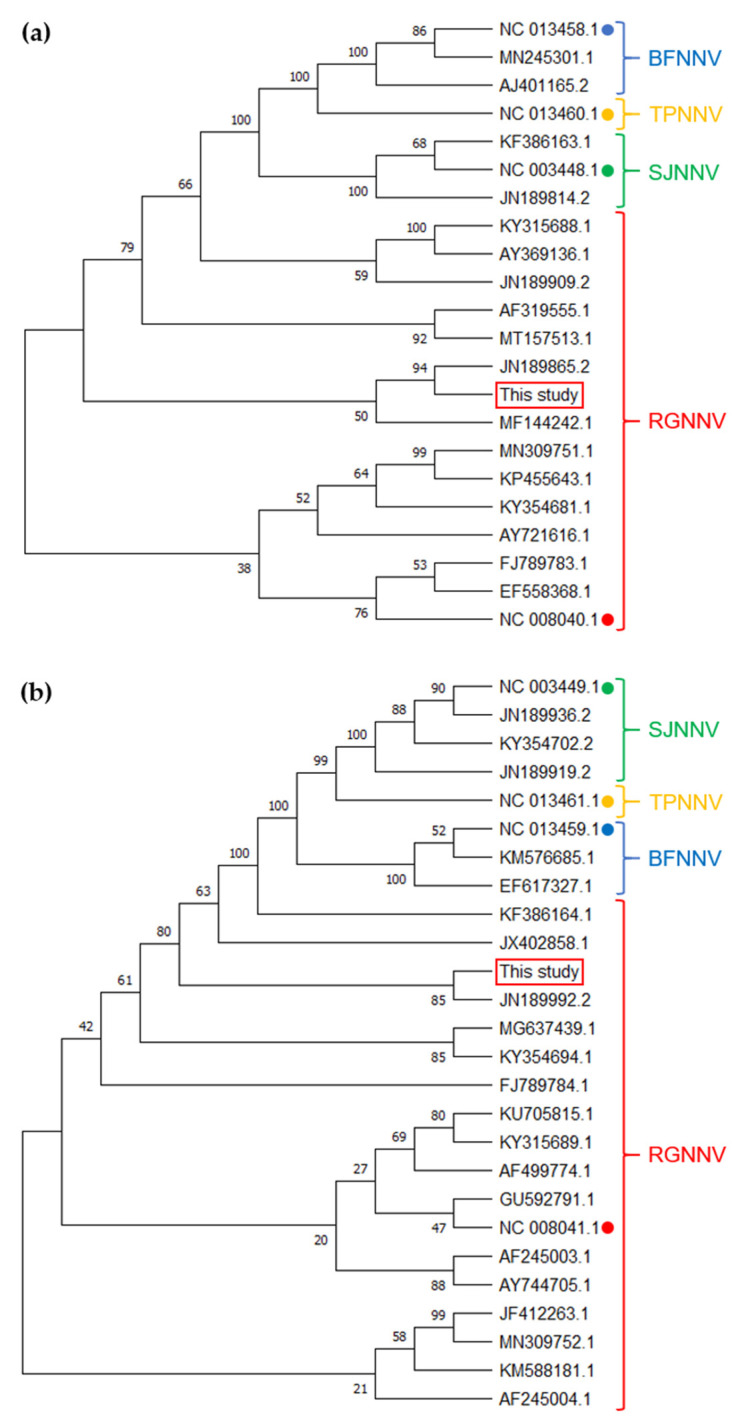
Phylogenetic analysis of both RNA1 (**a**) and RNA2 (**b**) genome-amplified fragments from the isolate of this study. The phylogenetic tree was generated, including complete nucleotide sequences of all NNV genotypes deposited in GenBank (listed in Tables S3 and S4 for RNA1 and RNA2, respectively, and indicated by their accession numbers here). The sequences were aligned with CLUSTAL W, and the tree calculated with MEGA X using the Neighbor–Joining method with Maximum Composite Likelihood on evolutionary distances as the optimality criterion. Numbers on the branches indicate the bootstrap support using distance. Genotype groups and their reference sequences are indicated with accordingly colored keys and dots, respectively. The sequences from this study are in boxes.

The sequences of the obtained PCR products, with lengths of 778 and 987 bp for RNA1 and RNA2, respectively, further confirmed the identity of NNV as the causative agent affecting the diseased fish (Figure S3). BLAST screenings of both amplified fragments (Tables S3 and S4 for RNA1 and RNA2, respectively) showed the highest identity values with RGNNV, and particularly with both genome segments of the RGNNV 283.2009 isolate—i.e., 99.49% for RNA1 (GB ID: JN189865.2) and 99.59% for RNA2 (GB ID: JN189992.2). Similarly, the sequences of this work also branched with those from the 283.2009 isolate within the RGNNV group in the phylogenetic tree drawn, including complete sequences from all NNV genotypes with less than 99% identity between them retrieved from the GenBank database (Figure 2).

The RGNNV 283.2009 isolate was rescued from farmed European seabass in Italy and classified into one of the most predominant phylogenetic clusters in the Mediterranean Sea based on the broad screening performed by Panzarin et al. (2012) [35] from samples collected between 2000 and 2009. Moreover, following in vivo studies aimed at assessing the pathogenicity of different Mediterranean isolates belonging to RGNNV and SJNNV genotypes and their both-sense reassortants, the 283.2009 isolate was revealed as the most pathogenic one in European seabass juveniles that had been bath-infected [61,62]. In these studies, primarily the latter one, it is also shown that mortality significantly increases with temperature in RGNNV infections and particularly with this isolate, reaching optimal values at 25–30 °C. These results confirm in vitro replication assessments in response to temperature using some of the same isolates, including 283.2009 [63]. Recently, the 283.2009 isolate has also shown a high lethality when experimentally infecting three different populations of European seabass juveniles, although with different mortality rates seen between them (47.8–85.8%), probably due to natural protection peculiarities [64].

### 3.2. NNV Physical Inactivations

#### 3.2.1. Heat Treatment

**Figure 3:**
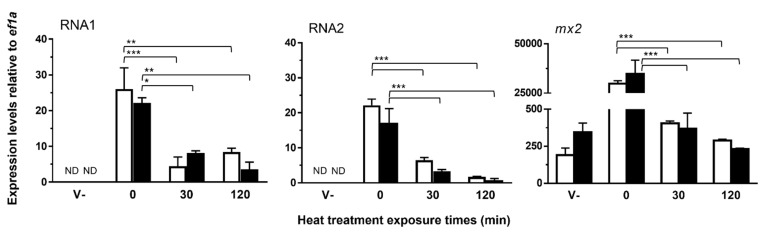
Effect of heat treatment on the infectivity of NNV. After incubation at 60 °C for 0 (negative control), 30, or 120 min in the presence (black bars) or absence (white bars) of 1% BSA, these virus samples were inoculated to SAF-1 cells in order to determine their infectivity levels. Infectivity was measured as the expression levels of their genome fragments by qPCR. The transcription levels of the IFN reporter gene *mx2* were also analyzed on these samples. *ef1a* mRNA was used as an endogenous control to normalize data (×10^6^), which are represented as the mean relative expression level ± SD of three different experiments performed in triplicate. Significant differences were determined by two-way ANOVA and Tukey’s multiple comparison test and indicated, excluding the comparisons with the “V-“ group, as: *, *p* < 0.05; **, *p* < 0.01; ***, *p* < 0.001. V-, control sample consisting of non-heated, clarified supernatants from non-infected E11 cells; ND, not detected.

The heat inactivation of NNV was performed by incubating virus-containing supernatants at 60 °C for 30 or 120 min in the presence or absence of 1% BSA. The replication of NNV genome fragments was measured by qPCR to determine the infectivity of the virus. As shown in Figure 3, the incubation time at 60 °C significantly inhibited the infectivity of the virus (*p* < 0.0001 for both RNA1 and RNA2), although no differences were found between the 30 and 120 min treatments. In contrast, the presence of BSA does not affect the infectivity of NNV when compared to corresponding PBS treatments. Regarding *mx2*, its expression was only increased in cells infected with non-heated virus samples.

To the best of our knowledge, only two previous studies have addressed the heat inactivation of NNV [55,65]. In the work of Frerichs et al. (2000) [55], the effect of various physical agents on the infectivity/inactivation of NNV was studied and, regarding temperature, this virus displayed a high thermal stability, as its infectivity was unaltered after at least 4 weeks at 25 °C or 1 day at 37 °C. Nevertheless, in the same work its heating at 60 °C revealed rapid inactivation, which was total at 30 min in the absence of FBS (just in Hanks’ balanced salt solution) or at 60 min with 10% FBS. A similar time trend appeared to occur in the present study, although here the potential action of an interfering organic material (BSA) was negligible and the replication was not entirely abolished even after 120 min of treatment. In a more recent study [65], the susceptibility of several NNV isolates to 60 °C with BSA was also reported, generally revealing a high resistance compared to other fish viruses. However, some differences were found in this sense between the four NNV isolates used—i.e., two of them were completely inactivated after 1 h of exposure, while the remaining two were highly inactivated by 1 h, and only reached whole inactivation at 24 h (the next sampling point checked).

Apart from potential variances in heat susceptibility between different isolates, it is also worth mentioning that most studies, including the above-cited ones, titrate the infectious NNV particles remaining in the treated suspensions using the Reed–Muench (TCID_50_) method. This contrasts with the qPCR method employed here and might lead to slight discrepancies between studies because of sensitivity differences. In this sense, even if qPCR has already been reported as a cost-effective, reliable, targeted, and sensitive NNV detection tool [66,67], given the possibility of amplifying non-relevant fragments (from residual non-infective virions, for instance) the validation of these results by other methods may be needed. In the present study, due to its primary objectives, this validation was only performed to confirm completely inactivated samples.

In any case, in view of all the available data, the total inactivation of this virus requires more than 2 h and up to 24 h of exposure to 60 °C. However, it should be noted that no study to date has proven the suitability of any temperature for generating thermally inactivated NNV vaccines that confer immunity and/or prevent disease. For this purpose, it is necessary not only to achieve a total inactivation of the pathogen, but also, generally, not to affect the native structure of the viral proteins and, therefore, their antigenicity under normal conditions, which is a determining factor for the induction of (usually conformational) neutralizing antibodies [8,68]. For all this, along with the fact that many successful thermally inactivated vaccines are generated at 56–60 °C [8,68], treatments at 60 °C have been used here and in the discussed preliminary NNV inactivation studies as a starting point to study the effect of temperature on this virus. Thus, further studies on NNV combining different genotypes/isolates, temperatures, and treatment times, together with the analysis of the protection conferred, are necessary in this regard for this virus, and even more so considering that NNV epitopes for generating neutralizing antibodies (in rabbit and mouse) have been found to present conformational structures sensitive to heat denaturation [69].

#### 3.2.2. UV-C Irradiation

**Figure 4:**
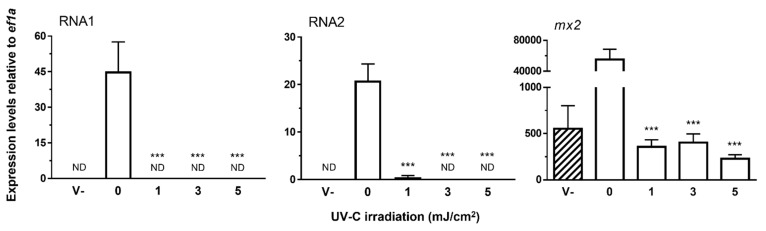
Effect of UV-C irradiation on the infectivity of NNV. After irradiation with 0 (untreated samples), 1, 3 or 5 mJ/cm^2^ of UV light at a wavelength of 254 nm, virus samples were inoculated to SAF-1 cells in order to determine their infectivity levels. Infectivity was measured according to the expression levels of their genome fragments by qPCR. The transcription levels of the IFN reporter gene *mx2* were also analyzed in these samples. *ef1a* mRNA was used as an endogenous control to normalize data (×10^6^), which are represented as the mean relative expression level ± SD of three different experiments performed in triplicate. Significant differences were determined by one-way ANOVA and Tukey’s multiple comparison test and indicated excluding the comparisons with the “V-” group with respect to the untreated group as: ***, *p* < 0.001. V- (striped bars), control sample consisting of non-irradiated, clarified supernatants from non-infected E11 cells; ND, not detected.

The effect of irradiating NNV with UV-C light was determined as for the heat treatments, but the treatment of virus-containing supernatants consisted of irradiation with 1, 3, and 5 J/cm^2^ of UV light at a wavelength of 254 nm. The results obtained (Figure 4) showed that the UV-C irradiation of 3 and 5 mJ/cm^2^ totally inhibited the viral replication—i.e., no amplification of either RNA1 or RNA2 was detected. Likewise, no amplification of RNA1 was observed after irradiation at 1 J/cm^2^, although negligible levels of RNA2 expression were detected. Regarding *mx2*, the irradiated NNV samples could not induce its expression, and thus their transcript levels were similar to the basal ones (V-).

The existing literature on this particular experimental system—i.e., UV-C inactivation of NNV particles—is scarce. In the only study that includes a dose–response assay, the irradiation required to completely inactivate the viral particles is much higher than that used in the present study, probably due to the higher titers of virus used. Specifically, Frerichs et al. (2000) [55] reported a linear decrease in NNV infection capacity with UV-C irradiation, reaching a total inhibition at 264 mJ/cm^2^. Likewise, Valero et al. (2018) [16] recently followed this procedure and exposed NNV particles to 800 mJ/cm^2^ UV-C irradiation to ensure their complete inactivation for vaccination purposes. Therein, notable protection to infection in European seabass juveniles intraperitoneally vaccinated with UV-inactivated NNV particles was demonstrated. Furthermore, it has also been shown that, among the broad list of immune-relevant genes analyzed, those closely involved in the type I IFN pathway (and particularly, *ifn*, *mx*, *stat1* and *isg15*) were either unaltered or induced in response to this vaccine. The latter was dependent upon the time point or tissue tested. Thus, in the present study it was not surprising to observe that in the SAF-1 cell line inoculated with UV-inactivated NNV particles, the levels of *mx2* expression were unaffected.

### 3.3. NNV Chemical Inactivation: Formalin Treatment

**Figure 5:**
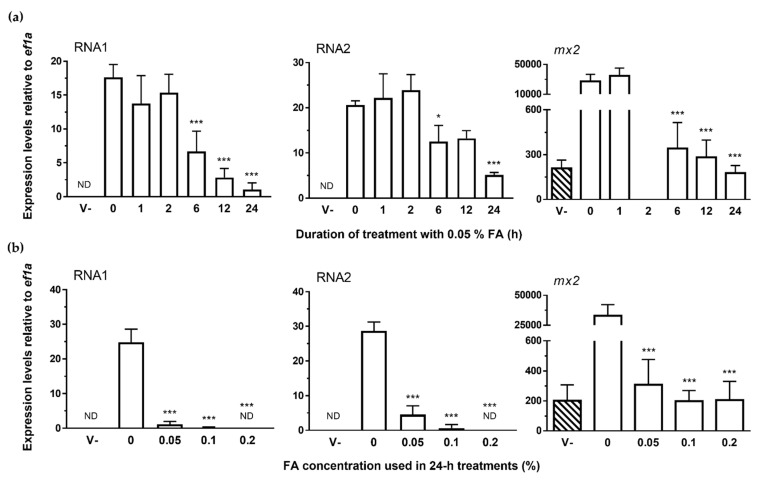
Effect of formalin treatment on the infectivity of NNV. NNV samples were treated with 0.05% formalin over different time points (**a**) and at different concentrations for up to 24 h (**b**). Then, the treated samples were inoculated to SAF-1 cells in order to determine their infectivity levels. Infectivity was measured as the expression levels of their genome fragments by qPCR. The transcription levels of the IFN reporter gene *mx2* were also analyzed in these samples. *ef1a* mRNA was used as an endogenous control to normalize data (×10^6^), which are represented as the mean relative expression level ± SD of three different experiments performed in triplicate. Significant differences were determined by one-way ANOVA and Tukey’s multiple comparison test and indicated, excluding the comparisons with the “V-” group, with respect to the untreated group as: *, *p* < 0.05, ***, *p* < 0.001. V- (striped bars), control sample consisting of non-treated, clarified supernatants from non-infected E11 cells; ND, not detected.

In order to assess the ability of formalin to inactivate NNV particles, NNV-containing supernatants were treated with 0.05% formalin for 1, 2, 6, 12, and 24 h at 25 °C and then inoculated to confluent SAF-1 monolayers to determine their viral loads and *mx2* expression by qRT-PCR at 48 h post-infection. As shown in Figure 5a, the treatment with formalin inhibited the infective ability of NNV particles in a time-dependent manner. Such an effect was only observable after the 6 h incubation period, which included the analysis of the replication of both NNV genome segments, but mostly RNA1. As occurred in previous treatments, when the viral infectivity was substantially reduced *mx2* showed equivalent expression levels to basal conditions.

Given that the incubations with 0.05% formalin for up to 24 h did not achieve the complete inactivation of the NNV particles, these were then tested post-24 h incubations under the same experimental conditions with increasing concentrations of formalin (0.05, 0.1, and 0.2%) (Figure 5b). These experiments displayed that NNV particles were also inactivated in a concentration-dependent manner by formalin. Complete virus inactivation was only achieved using 0.2% formalin (no detection of either NNV RNA1 or RNA2). It is of note that no characteristic toxic effects on cells, which are potentially associated with the treatments used, were observed. Furthermore, this same inactivation protocol with higher virus titers was also found to completely inactivate up to 10^8^ CCID_50_/mL of NNV particles (data not shown).

Despite being the most popular pathogen-inactivating agent used for antigen production applications, only a few studies have reported the effect of formalin on NNV in dose–response assays. Short incubations performed by Frerichs et al. (2000) [55] showed that 2 and 0.025% formalin treatments for up to 6 h at 15 °C resulted in falls in several logs in virus titers but were still far from achieving complete inactivation, as reported here. Based on the literature [9,44] and in line with our results, extended incubation periods with formalin are required to achieve the complete inactivation of NNV, and temperature is a crucial factor in this regard. For instance, in the work of Yamashita et al. (2005) [44], incubations of NNV with different concentrations of formalin at 4 °C reduced its titers below quantifiable levels after 1 and 3 days using 1% and 0.5% formalin, respectively. In contrast, NNV titers were only reduced a few logs after up to 10 days of incubation with 0.2% formalin at 4 °C in another study [9]. In this latest study [9], titers below quantifiable levels were reached in incubations with 0.1% formalin after 5 days at 24 °C or 2 days at 30 °C, and with 0.2% after 3 and 1 days at 24 and 30 °C, respectively. However, observable CPE after 3 successive passages was only abolished in incubations with 0.2% formalin after 7 and 3 days at 24 and 30 °C, respectively. All these differences might be due to the virus strain used, and thus authors using this method for vaccination trials usually opt for higher doses varying in formalin percentage, incubation time, and temperature in order to ensure full NNV inactivation and the safety of the final antigen product [46,70,71,72].

The results on virus inactivation shown here are closely related to those obtained by Rivas-Aravena et al. (2015) [10]. Therein, equivalent assays performed on infectious salmon anemia virus (ISAV) particles found them to be resistant to heat treatments of up to 70 °C for 2 h. However, the complete inactivation of ISAV particles was achieved with 9 mJ/cm^2^ UV-C irradiation or by the use of 0.2% formalin treatments for 3 h. In the present study, treatments consisting of 3 mJ/cm^2^ UV-C irradiation or incubation with 0.2% formalin for 24 h achieved the complete inactivation of the NNV particles for the strain isolated in this work. The efficiency of such inactivations was further confirmed by the total absence of any typical NNV CPE observed in SAF-1 cells after three successive blind-passages of 7 days duration each.

Nevertheless, the ease of scaling up the formalin inactivation method for use in industrial production; the extensive research on the use of this method for developing viral vaccines, including NNV ones [9,44,46,70,71]; and the widely extended legal laxity for their implementation mean that the majority of commercial vaccines are formalin-inactivated [3,4,5,6,7]. In fact, preliminary in vivo assays carried out to test the efficacy of a formalin-inactivated vaccine based on this study achieved a 100% relative percentage of survival (RPS) in a pilot experiment with a reduced number of European seabass (*Dicentrarchus labrax*) juveniles, confirming the results of previous analogous studies [9,44,46,70,71]. Furthermore, the viral load detected in survivors and the number of persistent infection carriers were notably lower in the immunized groups. This is important for limiting the virus’ further vertical and horizontal spread, which is driven by viral persistence. Persistence was identified as it is a significant problem in the case of NNV [73]. Whether the vaccine-induced protection from developing the persistent infection is related to antibody-based virus opsonization or blocking the induction of the Programmed Death-ligand 1 (PD-L1) and lymphocyte activation gene 3 (LAG3) negative regulatory factors of T-cells. The latter are most likely to be responsible for the development of NNV persistence [73], necessitating its exploration in further studies.

## 4. Conclusions

Herein, we have demonstrated three inactivation procedures for a new highly pathogenic NNV isolate with a high sequence homology to the RGNNV 283.2009 isolate using heat inactivation, UV-C irradiation, or formalin treatment to generate viral antigens for use in aquaculture vaccination regimes. The widespread pathological effects of the NNV strain isolated in this work was observed as a massive vacuolization in E11 and SAF-1 cell monolayers. Owing to the in vitro detection of NNV RNA1 and RNA2 in rescued isolates via RT-PCR, this confirmed NNV infections as the causative symptomatic and visceral agents in vitro. The virus particles of this isolate showed a marked resistance to heat (for at least 60 °C for 120 min with and without 1% BSA), but they were fully inactivated with 3 mJ/cm^2^ UV-C irradiation and 24 h 0.2% formalin treatments. Furthermore, notable reductions in the infectivity of NNV in SAF-1 monolayers were not observed until 6 h post-treatment with 0.05% formalin. In fact, formalin incubation periods exceeding 24 h were required before significant reductions in NNV RNA1 and RNA2 were detected. Future studies in this area should now aim to address these variable effects of NNV inactivation in vivo in order to develop rapid response strategies for VNN control in aquaculture.

## Figures and Tables

**Table 1:** Primers used for gene expression analysis by qPCR.

Gene	Species	GB ID	Sequence (5′-3′)	Length	Ref.
*rdrp*	NNV	-	F: CCGATATCACGATGAGTTCAC R: GAAAGCGTAGGTGTAGCTGG	139	This study
*cp*	NNV	-	F: CGCTGCTAGAATCTTCCA R: CTTTCCCACCATTTGGC	183	This study
*mx2*	Sea bream	FJ490555	F: AAGAGGAGGACGAGGAGGAG R: TTCAGGTGCAGCATCAACTC	148	[58,59]
*ef1a*	Sea bream	AF184170	F: CTGTCAAGGAAATCCGTCGT R: TGACCTGAGCGTTGAAGTTG	87	[60]

GB ID: GenBank accession number; length: amplicon length/size in base pairs; F: forward primer; R: reverse primer.

## Data Availability

The data presented in this study are available on request from the corresponding author.

## Supplementary Materials

The following are available online at https://www.mdpi.com/article/10.3390/vaccines9121441/s1: Table S1: Representative genotype sequences for designing broad-spectrum NNV RNA1 amplification primers; Table S2: Representative genotype sequences for designing broad-spectrum NNV RNA2 amplification primers; Table S3: BLASTn top resulting records similar to the RNA1 sequence from the NNV isolated in this work; Table S4: BLASTn top resulting records similar to the RNA2 sequence from the NNV isolated in this work; Figure S1: Multiple sequence alignment of RNA1 sequences from the selected genotype representatives; Figure S2: Multiple sequence alignment of RNA2 sequences from the selected genotype representatives; Figure S3: Sequences of the RNA1 and RNA2 fragments of the NNV isolate from this study.

## References

[1] De Clercq E., Li G. (2016). Approved antiviral drugs over the past 50 years. Clin. Microbiol. Rev..

[2] Clem A.S. (2011). Fundamentals of vaccine immunology. J. Glob. Infect. Dis..

[3] Salgado-Miranda C., Loza-Rubio E., Rojas-Anaya E., García-Espinosa G. (2013). Viral vaccines for bony fish: Past, present and future. Expert Rev. Vaccines.

[4] Dhar A.K., Manna S.K., Allnutt F.T. (2014). Viral vaccines for farmed finfish. Virusdisease.

[5] Chambers M.A., Graham S.P., La Ragione R.M. (2016). Challenges in veterinary vaccine development and immunization. Vaccine Des..

[6] Ma J., Bruce T.J., Jones E.M., Cain K.D. (2019). A review of fish vaccine development strategies: Conventional methods and modern biotechnological approaches. Microorganisms.

[7] Ike A.C., Ononugbo C.M., Obi O.J., Onu C.J., Olovo C.V., Muo S.O., Chukwu O.S., Reward E.E., Omeke O.P. (2021). Towards Improved Use of Vaccination in the Control of Infectious Bronchitis and Newcastle Disease in Poultry: Understanding the Immunological Mechanisms. Vaccines.

[8] Sanders B., Koldijk M., Schuitemaker H. (2015). Inactivated viral vaccines. Vaccine Analysis: Strategies, Principles, and Control.

[9] Kai Y.-H., Chi S.-C. (2008). Efficacies of inactivated vaccines against betanodavirus in grouper larvae (*Epinephelus coioides*) by bath immunization. Vaccine.

[10] Rivas-Aravena A., Fuentes Y., Cartagena J., Brito T., Poggio V., La Torre J., Mendoza H., Gonzalez-Nilo F., Sandino A.M., Spencer E. (2015). Development of a nanoparticle-based oral vaccine for Atlantic salmon against ISAV using an alphavirus replicon as adjuvant. Fish Shellfish Immunol..

[11] Zeng W., Wang Y., Hu H., Wang Q., Bergmann S.M., Wang Y., Li B., Lv Y., Li H., Yin J. (2021). Cell Culture-Derived Tilapia Lake Virus-Inactivated Vaccine Containing Montanide Adjuvant Provides High Protection against Viral Challenge for Tilapia. Vaccines.

[12] Zhang L., Ma J., Fan Y., Zhou Y., Xu J., Liu W., Gu Z., Zeng L. (2016). Immune response and protection in gibel carp, *Carassius gibelio*, after vaccination with β-propiolactone inactivated cyprinid herpesvirus 2. Fish Shellfish Immunol..

[13] Lopez-Doriga M., Smail D., Smith R., Domenech A., Castric J., Smith P., Ellis A. (2001). Isolation of salmon pancreas disease virus (SPDV) in cell culture and its ability to protect against infection by the ‘wild-type’agent. Fish Shellfish Immunol..

[14] Hwang J.Y., Kwon M.G., Jung S.-H., Park M.A., Kim D.-W., Cho W.S., Park J.W., Son M.-H. (2017). RNA-Seq transcriptome analysis of the olive flounder (*Paralichthys olivaceus*) kidney response to vaccination with heat-inactivated viral hemorrhagic septicemia virus. Fish Shellfish Immunol..

[15] Hwang J.Y., Kwon M.-G., Kim Y.J., Jung S.-H., Park M.-A., Son M.-H. (2017). Montanide IMS 1312 VG adjuvant enhances the efficacy of immersion vaccine of inactivated viral hemorrhagic septicemia virus (VHSV) in olive flounder, *Paralichthys olivaceus*. Fish Shellfish Immunol..

[16] Valero Y., Mokrani D., Chaves-Pozo E., Arizcun M., Oumouna M., Meseguer J., Esteban M.Á., Cuesta A. (2018). Vaccination with UV-inactivated nodavirus partly protects European sea bass against infection, while inducing few changes in immunity. Dev. Comp. Immunol..

[17] Brudeseth B.E., Wiulsrød R., Fredriksen B.N., Lindmo K., Løkling K.-E., Bordevik M., Steine N., Klevan A., Gravningen K. (2013). Status and future perspectives of vaccines for industrialised fin-fish farming. Fish Shellfish Immunol..

[18] Schulz P., Terech-Majewska E., Siwicki A.K., Kazuń B., Demska-Zakęś K., Rożyński M., Zakęś Z. (2020). Effect of Different Routes of Vaccination against Aeromonas salmonicida on Rearing Indicators and Survival after an Experimental Challenge of Pikeperch (*Sander lucioperca*) in Controlled Rearing. Vaccines.

[19] Costa J.Z., Thompson K.D. (2016). Understanding the interaction between Betanodavirus and its host for the development of prophylactic measures for viral encephalopathy and retinopathy. Fish Shellfish Immunol..

[20] Yong C.Y., Yeap S.K., Omar A.R., Tan W.S. (2017). Advances in the study of nodavirus. PeerJ.

[21] Iwamoto T., Mise K., Takeda A., Okinaka Y., Mori K.-I., Arimoto M., Okuno T., Nakai T. (2005). Characterization of Striped jack nervous necrosis virus subgenomic RNA3 and biological activities of its encoded protein B2. J. Gen. Virol..

[22] Fenner B.J., Goh W., Kwang J. (2006). Sequestration and protection of double-stranded RNA by the betanodavirus B2 protein. J. Virol..

[23] Chen N.-C., Yoshimura M., Guan H.-H., Wang T.-Y., Misumi Y., Lin C.-C., Chuankhayan P., Nakagawa A., Chan S.I., Tsukihara T. (2015). Crystal structures of a piscine betanodavirus: Mechanisms of capsid assembly and viral infection. PLoS Pathog..

[24] NaveenKumar S., Shekar M., Karunasagar I., Karunasagar I. (2013). Genetic analysis of RNA1 and RNA2 of *Macrobrachium rosenbergii* nodavirus (MrNV) isolated from India. Virus Res..

[25] Yoshikoshi K., Inoue K. (1990). Viral nervous necrosis in hatchery-reared larvae and juveniles of Japanese parrotfish, *Oplegnathus fasciatus* (Temminck & Schlegel). J. Fish Dis..

[26] Munday B., Kwang J., Moody N. (2002). Betanodavirus infections of teleost fish: A review. J. Fish Dis..

[27] Shetty M., Maiti B., Santhosh K.S., Venugopal M.N., Karunasagar I. (2012). Betanodavirus of marine and freshwater fish: Distribution, genomic organization, diagnosis and control measures. Indian J. Virol..

[28] Arimoto M., Mushiake K., Mizuta Y., Nakai T., Muroge K., Furusawa I. (1992). Detection of striped jack nervous necrosis virus (SJNNV) by enzyme-linked immunosorbent assay (ELISA). Fish Pathol..

[29] Grotmol S., Totland G.K. (2000). Surface disinfection of Atlantic halibut Hippoglossus hippoglossus eggs with ozonated sea-water inactivates nodavirus and increases survival of the larvae. Dis. Aquat. Org..

[30] Skliris G.P., Krondiris J.V., Sideris D.C., Shinn A.P., Starkey W.G., Richards R.H. (2001). Phylogenetic and antigenic characterization of new fish nodavirus isolates from Europe and Asia. Virus Res..

[31] Fukuda Y., Furuhashi M., Nakai T. (1996). Mass mortality of cultured sevenband grouper, *Epinephelus septemfasciatus*, associated with viral nervous necrosis. Fish Pathol..

[32] Munday B., Nakai T. (1997). Nodaviruses as pathogens in larval and juvenile marine finfish. World J. Microbiol. Biotechnol..

[33] Le Breton A., Grisez L., Sweetman J., Ollevier F. (1997). Viral nervous necrosis (VNN) associated with mass mortalities in cage-reared sea bass, *Dicentrarchus labrax* (L.). J. Fish Dis..

[34] Glazebrook J., Campbell R. (1987). Diseases of barramundi (*Lates calcarifer*) in Australia: A review. Management of Wild and Cultured Sea Bass/Barramundi.

[35] Panzarin V., Fusaro A., Monne I., Cappellozza E., Patarnello P., Bovo G., Capua I., Holmes E.C., Cattoli G. (2012). Molecular epidemiology and evolutionary dynamics of betanodavirus in southern Europe. Infect. Genet. Evol..

[36] Wu M.S., Chen C.W., Lin C.H., Tzeng C.S., Chang C.Y. (2012). Differential expression profiling of orange-spotted grouper larvae, *Epinephelus coioides* (Hamilton), that survived a betanodavirus outbreak. J. Fish Dis..

[37] Tanaka S., Mori K., Arimoto M., Iwamoto T., Nakai T. (2001). Protective immunity of sevenband grouper, *Epinephelus septemfasciatus* Thunberg, against experimental viral nervous necrosis. J. Fish Dis..

[38] Nishizawa T., Mori K.-i., Furuhashi M., Nakai T., Furusawa I., Muroga K. (1995). Comparison of the coat protein genes of five fish nodaviruses, the causative agents of viral nervous necrosis in marine fish. J. Gen. Virol..

[39] Nishizawa T., Furuhashi M., Nagai T., Nakai T., Muroga K. (1997). Genomic classification of fish nodaviruses by molecular phylogenetic analysis of the coat protein gene. Appl. Environ. Microbiol..

[40] Toffolo V., Negrisolo E., Maltese C., Bovo G., Belvedere P., Colombo L., Dalla Valle L. (2007). Phylogeny of betanodaviruses and molecular evolution of their RNA polymerase and coat proteins. Mol. Phylogenetics Evol..

[41] Olveira J.G., Souto S., Dopazo C.P., Thiéry R., Barja J.L., Bandín I. (2009). Comparative analysis of both genomic segments of betanodaviruses isolated from epizootic outbreaks in farmed fish species provides evidence for genetic reassortment. J. Gen. Virol..

[42] Mori K.-i., Mangyoku T., Iwamoto T., Arimoto M., Tanaka S., Nakai T. (2003). Serological relationships among genotypic variants of betanodavirus. Dis. Aquat. Org..

[43] Costa J.Z. (2005). B Cell Epitopes in Fish Nodavirus. https://dspace.stir.ac.uk/bitstream/1893/13240/1/COSTA%202005.pdf.

[44] Yamashita H., Fujita Y., Kawakami H., Nakai T. (2005). The efficacy of inactivated virus vaccine against viral nervous necrosis (VNN). Fish Pathol..

[45] Pakingking R., Seron R., Dela Peña L., Mori K., Yamashita H., Nakai T. (2009). Immune responses of Asian sea bass, *Lates calcarifer* Bloch, against an inactivated betanodavirus vaccine. J. Fish Dis..

[46] Pakingking R., Bautista N.B., de Jesus-Ayson E.G., Reyes O. (2010). Protective immunity against viral nervous necrosis (VNN) in brown-marbled grouper (*Epinephelus fuscogutattus*) following vaccination with inactivated betanodavirus. Fish Shellfish Immunol..

[47] Bejar J., Borrego J.J., Alvarez M.C. (1997). A continuous cell line from the cultured marine fish gilt-head seabream (*Sparus aurata* L.). Aquaculture.

[48] Iwamoto T., Nakai T., Mori K.-i., Arimoto M., Furusawa I. (2000). Cloning of the fish cell line SSN-1 for piscine nodaviruses. Dis. Aquat. Org..

[49] Frerichs G., Morgan D., Hart D., Skerrow C., Roberts R., Onions D. (1991). Spontaneously productive C-type retrovirus infection of fish cell lines. J. Gen. Virol..

[50] Reed L.J., Muench H. (1938). A simple method of estimating fifty per cent endpoints. Am. J. Epidemiol..

[51] Cattoli G. (2019). Viral encephalopathy and retinopathy. Manual of Diagnostic Tests for Aquatic Animals.

[52] Falco A., Chico V., Marroqui L., Perez L., Coll J., Estepa A. (2008). Expression and antiviral activity of a β-defensin-like peptide identified in the rainbow trout (*Oncorhynchus mykiss*) EST sequences. Mol. Immunol..

[53] Madeira F., Park Y.M., Lee J., Buso N., Gur T., Madhusoodanan N., Basutkar P., Tivey A.R., Potter S.C., Finn R.D. (2019). The EMBL-EBI search and sequence analysis tools APIs in 2019. Nucleic Acids Res..

[54] Kumar S., Stecher G., Li M., Knyaz C., Tamura K. (2018). MEGA X: Molecular evolutionary genetics analysis across computing platforms. Mol. Biol. Evol..

[55] Frerichs G., Tweedie A., Starkey W., Richards R. (2000). Temperature, pH and electrolyte sensitivity, and heat, UV and disinfectant inactivation of sea bass (*Dicentrarchus labrax*) neuropathy nodavirus. Aquaculture.

[56] Bello-Perez M., Pereiro P., Coll J., Novoa B., Perez L., Falco A. (2020). Zebrafish C-reactive protein isoforms inhibit SVCV replication by blocking autophagy through interactions with cell membrane cholesterol. Sci. Rep..

[57] Livak K.J., Schmittgen T.D. (2001). Analysis of relative gene expression data using real-time quantitative PCR and the 2^−ΔΔCT^ method. Methods.

[58] Chaves-Pozo E., Guardiola F.A., Meseguer J., Esteban M.A., Cuesta A. (2012). Nodavirus infection induces a great innate cell-mediated cytotoxic activity in resistant, gilthead seabream, and susceptible, European sea bass, teleost fish. Fish Shellfish Immunol..

[59] Leiva-Rebollo R., Labella A., Borrego J., Castro D. (2020). Immune gene expression in gilthead seabream (*Sparus aurata*) after Lymphocystis disease virus (LCDV-Sa) challenge resulting in asymptomatic infection. J. Appl. Microbiol..

[60] Cerezuela R., Meseguer J., Esteban M.Á. (2013). Effects of dietary inulin, Bacillus subtilis and microalgae on intestinal gene expression in gilthead seabream (*Sparus aurata* L.). Fish Shellfish Immunol..

[61] Vendramin N., Toffan A., Mancin M., Cappellozza E., Panzarin V., Bovo G., Cattoli G., Capua I., Terregino C. (2014). Comparative pathogenicity study of ten different betanodavirus strains in experimentally infected E uropean sea bass, *Dicentrarchus labrax* (L.). J. Fish Dis..

[62] Toffan A., Panzarin V., Toson M., Cecchettin K., Pascoli F. (2016). Water temperature affects pathogenicity of different betanodavirus genotypes in experimentally challenged *Dicentrarchus labrax*. Dis. Aquat. Org..

[63] Panzarin V., Cappellozza E., Mancin M., Milani A., Toffan A., Terregino C., Cattoli G. (2014). In vitro study of the replication capacity of the RGNNV and the SJNNV betanodavirus genotypes and their natural reassortants in response to temperature. Vet. Res..

[64] Barsøe S., Allal F., Vergnet A., Vandeputte M., Olesen N.J., Schmidt J.G., Larsen C.A., Cuenca A., Vendramin N. (2021). Different survival of three populations of European sea bass (*Dicentrarchus labrax*) following challenge with two variants of nervous necrosis virus (NNV). Aquac. Rep..

[65] Dixon P., Smail D., Algoët M., Hastings T., Bayley A., Byrne H., Dodge M., Garden A., Joiner C., Roberts E. (2012). Studies on the effect of temperature and pH on the inactivation of fish viral and bacterial pathogens. J. Fish Dis..

[66] Dalla Valle L., Toffolo V., Lamprecht M., Maltese C., Bovo G., Belvedere P., Colombo L. (2005). Development of a sensitive and quantitative diagnostic assay for fish nervous necrosis virus based on two-target real-time PCR. Vet. Microbiol..

[67] Panzarin V., Patarnello P., Mori A., Rampazzo E., Cappellozza E., Bovo G., Cattoli G. (2010). Development and validation of a real-time TaqMan PCR assay for the detection of betanodavirus in clinical specimens. Arch. Virol..

[68] Delrue I., Verzele D., Madder A., Nauwynck H.J. (2012). Inactivated virus vaccines from chemistry to prophylaxis: Merits, risks and challenges. Expert Rev. Vaccines.

[69] Gye H.J., Park M.-J., Kim W.-S., Oh M.-J., Nishizawa T. (2018). Heat-denaturation of conformational structures on nervous necrosis virus for generating neutralization antibodies. Aquaculture.

[70] Yamashita H., Mori K., Kuroda A., Nakai T. (2009). Neutralizing antibody levels for protection against betanodavirus infection in sevenband grouper, *Epinephelus septemfasciatus* (Thunberg), immunized with an inactivated virus vaccine. J. Fish Dis..

[71] Nuñez-Ortiz N., Pascoli F., Picchietti S., Buonocore F., Bernini C., Toson M., Scapigliati G., Toffan A. (2016). A formalin-inactivated immunogen against viral encephalopathy and retinopathy (VER) disease in European sea bass (*Dicentrarchus labrax*): Immunological and protection effects. Vet. Res..

[72] Valero Y., Olveira J.G., López-Vázquez C., Dopazo C.P., Bandín I. (2021). BEI Inactivated Vaccine Induces Innate and Adaptive Responses and Elicits Partial Protection upon Reassortant Betanodavirus Infection in Senegalese Sole. Vaccines.

[73] Tso C.-H., Lu M.-W. (2018). Transcriptome profiling analysis of grouper during nervous necrosis virus persistent infection. Fish Shellfish Immunol..

