# Supplementary material for: Update on the Inactivation Procedures for the Vaccine Development Prospects of a New Highly Virulent RGNNV Isolate
Source: Vaccines (Basel). 2021 Dec 7;9(12):1441. doi: 10.3390/vaccines9121441 (PMC8705346; doi:10.3390/vaccines9121441)
Supplement: Supplementary file 1 [file vaccines-09-01441-s001.zip › vaccines-1461102-supplementary.pdf]

Supplementary Materials

Update on the inactivation procedures for the vaccine development prospects of a new highly virulent RGNNV isolate

Alberto Falco [1,*], Melissa Bello-Perez [1], Rocío Díaz-Puertas [1], Matthew Mold [2] and Mikolaj Adamek [3]

[1] Institute of Research, Development, and Innovation in Healthcare Biotechnology in Elche (IDiBE), Miguel Hernández University (UMH), 03202 Elche, Spain; alber.falco@umh.es (A.F.) melissa.bello@alu.umh.es (M.B.-P.); rocio.diaz01@goumh.umh.es (R.D.P.)
[2] The Birchall Centre, Lennard-Jones Laboratories, Keele University, Keele, Staffordshire, ST5 5BG, United Kingdom; matthew.j.mold@gmail.com (M.M.)
[3] Fish Disease Research Unit, Institute for Parasitology, University of Veterinary Medicine, 30559 Hannover, Germany; mikolaj.adamek@tiho-hannover.de (M.A.)
* Correspondence: alber.falco@umh.es (A.F.)

Table S1. Representative genotype sequences for designing broad-spectrum NNV RNA1 amplification primers.						
**Acc. no.**	**Isolate ref.**	**Genotype**	**Species host**		**Country**	**Ref.**
			**Common name**	**Scientific name**		
NC_013458.1	JFIwa98	BFNNV	Barfin flounder	*Verasper moseri*	Japan	[1]
NC_008040.1	SGWak97	RGNNV	Red-spotted grouper	*Epinephelus akaara*		[2]
NC_003448.1		SJNNV	Striped Jack	*Pseudocaranx dentex*		[3]
NC_013460.1	TPKag93	TPNNV	Tiger puffer	*Takifugu rubripes*	Japan	[1]
KF386163.1	389/I96	SJNNV	European seabass	*Dicentrarchus labrax*	Italy	[4]
JN189814.2	484.2.2009	SJNNV	Senegalese sole	*Solea senegalensis*	Spain	[5]
FJ789783.1	1	RGNNV	European seabass	*Dicentrarchus labrax*	Tunisia	[6]
JN189909.2	367.2.2005	RGNNV	European seabass	*Dicentrarchus labrax*	Italy	[5]
AF319555.1	Singapore	RGNNV	Greasy grouper	*Epinephelus tauvina*	Singapore	[7]
AY369136.1		RGNNV	Orange-spotted grouper	*Epinephelus coioides*	China	[1,8]
AY721616.1		RGNNV	Dragon grouper	*Epinephelus lanceolatus*	China	[1]
EF558368.1	China 1	RGNNV	Red-spotted grouper	*Epinephelus akaara*	China	[9]
JN189865.2	283.2009	RGNNV	European seabass	*Dicentrarchus labrax*	Italy	[5]
KP455643.1	SBN147	RGNNV	Sea Perch	*Lateolabrax japonicus*	China	[10]
MN309751.1	RGNNV201	RGNNV	Hybrid tiger grouper	*Epinephelus fuscoguttatus* x *Epinephelus lanceolatus*	China	[11]
MT157513.1	CNPgg2018	RGNNV	Pearl gentian grouper	*Epinephelus lanceolatus* x *Epinephelus fuscoguttatus*	China	[12]
AJ401165.2	AH99NorA	BFNNV	Atlantic halibut	*Hippoglossus hippoglossus*	Norway	[13]
MF144242.1	KS1	RGNNV	Orange-spotted grouper	*Epinephelus coioides*	Taiwan	[14]
KY315688.1	MENNV1310	RGNNV	Marbled eel	*Anguilla marmorata*	Taiwan	
KY354681.1	VNNV/S.aurata/I/425-10/Sep2008	RGNNV	Gilthead seabream	*Sparus aurata*	Italy	[15]
MN245301.1	SK-07 1324	BFNNV	Atlantic cod	*Gadus morhua*	Norway	[16]
Reference sequences are shaded.						

Table S2. Representative genotype sequences for designing broad-spectrum NNV RNA2 amplification primers.

Acc. no.	Isolate ref.	Genotype	Species host		Country	Ref.
			**Common name**	**Scientific name**		
NC_013459.1	JFIwa98	BFNNV	Barfin flounder	*Verasper moseri*	Japan	[1]
NC_008041.1	SGWak97	RGNNV	Red-spotted grouper	*Epinephelus akaara*		[2]
NC_003449.1		SJNNV	Striped Jack	*Pseudocaranx dentex*		[3]
NC_013461.1	TPKag93	TPNNV	Tiger puffer	*Takifugu rubripes*	Japan	[1]
KY315689.1	MENNV1310	RGNNV	Marbled eel	*Anguilla marmorata*	Taiwan	
AY744705.1	RGNNV-CN	RGNNV	Red-spotted grouper	*Epinephelus akaara*	China	[17]
JF412263.1	BVN-CIBA10	RGNNV	Barramundi	*Lates calcarifer*	India	
KF386164.1	389/I96	RGNNV	European seabass	*Dicentrarchus labrax*	Italy	[4]
MN309752.1	RGNNV201	RGNNV	Hybrid tiger grouper	*Epinephelus fuscoguttatus* x *Epinephelus lanceolatus*	China	[11]
EF617327.1	Ac06NorPp	BFNNV	Atlantic cod	*Gadus morhua*	Norway	
FJ789784.1	1	RGNNV	European seabass	*Dicentrarchus labrax*	Tunisia	[6]
JN189992.2	283.2009	RGNNV	European seabass	*Dicentrarchus labrax*	Italy	[5]
MG637439.1	ZN521	RGNNV	Pearl gentian grouper	*Epinephelus lanceolatus* x *Epinephelus fuscoguttatus*	China	
KM588181.1	GGNNV20131122	RGNNV	Giant grouper	*Epinephelus lanceolatus*	Taiwan	[18]
JN189919.2	484.2.2009	SJNNV	Senegalese sole	*Solea senegalensis*	Spain	[5]
JN189936.2	367.2.2005	SJNNV	European seabass	*Dicentrarchus labrax*	Italy	[5]
KY354694.1	VNNV/S.aurata/I/425-10/Sep2008	RGNNV	Gilthead seabream	*Sparus aurata*	Italy	
GU592791.1	LcNNV	RGNNV	Barramundi	*Lates calcarifer*	India	
KY354702.2	VNNV/S.aurata/Farm1/461-1/Nov2014	SJNNV	Gilthead seabream	*Sparus aurata*	Mediterranean Sea	[19]
AF245003.1		RGNNV	Malabar grouper	*Epinephelus malabaricus*	Taiwan	[20]
AF245004.1		RGNNV	Giant grouper	*Epinephelus lanceolatus*	Taiwan	[20]
KM576685.1	PCNNV	BFNNV	Pacific cod	*Gadus macrocephalus*	China	[21]
AF499774.1		RGNNV	Guppy	*Poicelia reticulata*	Singapore	[22]
KU705815.1	VI37	RGNNV	Mouse grouper	*Cromileptes altivelis*	Vietnam	[23]
JX402858.1	Tilapia-CU-2011	RGNNV	Nile tilapia	*Oreochromis niloticus*	Thailand	[24]

Reference sequences are shaded.

Table S3. BLASTn top resulting records similar to the RNA1 sequence from the NNV isolated in this work.								
**Accession**	**Coverage**	**Scientific Name**	**Max Score**	**Total Score**	**Query Cover**	**E value**	**Per. ident**	**Acc. Len**
JN189865.2	complete cds	Dicentrarchus labrax betanodavirus	1415	1415	100%	0.0	99.49	3103
MF144242.1	complete sequence	Epinephelus coioides nervous necrosis virus	1349	1349	100%	0.0	97.95	3100
KY930893.1	complete cds	Betanodavirus sp.	1349	1349	100%	0.0	97.95	2946
MH758753.1	complete cds	Redspotted grouper nervous necrosis virus	1343	1343	100%	0.0	97.82	3024
KC696563.1	complete cds	Redspotted grouper nervous necrosis virus	1343	1343	100%	0.0	97.82	2991
AY324869.1	complete cds	Sevenband grouper nervous necrosis virus	1343	1343	100%	0.0	97.82	3105
GQ402010.1	complete cds	Redspotted grouper nervous necrosis virus	1338	1338	100%	0.0	97.69	2998
KY354681.1	complete cds	Sparus aurata betanodavirus	1332	1332	100%	0.0	97.56	3051
FJ809938.1	complete cds	Redspotted grouper nervous necrosis virus	1332	1332	100%	0.0	97.56	2949
FJ803915.1	complete cds	Sea bass Iberian betanodavirus	1327	1327	100%	0.0	97.43	3089
AY690597.1	partial cds	Redspotted grouper nervous necrosis virus	1327	1327	100%	0.0	97.43	2968
KT390713.1	partial cds	Grouper betanodavirus	1321	1321	100%	0.0	97.30	2871
FJ748760.1	complete sequence	Japanese flounder nervous necrosis virus	1321	1321	100%	0.0	97.30	3104
AB373028.1	complete sequence	Sevenband grouper nervous necrosis virus	1321	1321	100%	0.0	97.30	3105
AF319555.1	complete cds	Epinephelus tauvina nervous necrosis virus	1321	1321	100%	0.0	97.30	3103
AF326776.1	complete sequence	Epinephelus tauvina nervous necrosis virus	1321	1321	100%	0.0	97.30	3007
MH181161.1	complete cds	Redspotted grouper nervous necrosis virus	1315	1315	100%	0.0	97.18	3090
HQ859913.1	complete cds	Tiger grouper Nervous Necrosis Virus	1315	1315	100%	0.0	97.18	3024
HQ859908.1	complete cds	Tiger grouper Nervous Necrosis Virus	1315	1315	100%	0.0	97.18	3024
HQ859892.1	complete cds	Mouse grouper Nervous Necrosis Virus	1315	1315	100%	0.0	97.18	3024
AY721616.1	complete cds	Dragon grouper nervous necrosis virus	1315	1315	100%	0.0	97.18	3103
HQ859895.1	complete cds	Tiger grouper Nervous Necrosis Virus	1310	1310	100%	0.0	97.05	3024
HQ859890.1	complete cds	Asian seabass Nervous Necrosis Virus	1310	1310	100%	0.0	97.05	3024
HQ859887.1	complete cds	Asian seabass Nervous Necrosis Virus	1310	1310	100%	0.0	97.05	3024
EF558368.1	complete cds	Redspotted grouper nervous necrosis virus	1310	1310	100%	0.0	97.05	3103
KM095958.1	complete sequence	Sevenband grouper nervous necrosis virus	1304	1304	100%	0.0	96.92	3103

MN097800.1	partial cds	Betanodavirus sp.	1299	1299	100%	0.0	96.79	2727
MN097798.1	complete cds	Betanodavirus sp.	1299	1299	100%	0.0	96.79	2961
HQ859916.1	complete cds	Asian seabass Nervous Necrosis Virus	1299	1299	100%	0.0	96.79	3024
HQ859911.1	complete cds	Asian seabass Nervous Necrosis Virus	1299	1299	100%	0.0	96.79	3024
FJ803913.1	complete cds	Gilthead sea bream Iberian betanodavirus	1299	1299	100%	0.0	96.79	3101
GQ904198.1	complete cds	Golden pompano nervous necrosis virus	1299	1299	100%	0.0	96.79	3024
FJ789783.1	complete sequence	Redspotted grouper nervous necrosis virus	1299	1299	100%	0.0	96.79	3104
KU705814.1	complete sequence	Mouse grouper Nervous Necrosis Virus	1293	1293	100%	0.0	96.66	3017
FJ803914.1	complete cds	Senegalese sole Iberian betanodavirus	1293	1293	100%	0.0	96.66	3042
FJ803912.1	complete cds	Gilthead sea bream Iberian betanodavirus	1293	1293	100%	0.0	96.66	3065
MG387122.1	complete cds	Betanodavirus sp.	1288	1288	100%	0.0	96.53	3105
KP455643.1	complete sequence	Sevenband grouper nervous necrosis virus	1288	1288	100%	0.0	96.53	3103
FJ803911.2	complete cds	Senegalese sole Iberian betanodavirus	1288	1288	100%	0.0	96.53	3105
KJ541747.1	complete cds	Redspotted grouper nervous necrosis virus	1288	1288	100%	0.0	96.53	3103
JN189909.2	complete cds	Dicentrarchus labrax betanodavirus	1288	1288	100%	0.0	96.53	3104
KY354688.2	complete sequence	Sparus aurata betanodavirus	1288	1288	100%	0.0	96.53	2984
FJ803916.1	complete cds	Gilthead sea bream Iberian betanodavirus	1288	1288	100%	0.0	96.53	3051
MG874757.1	complete sequence	Epinephelus coioides nervous necrosis virus	1282	1282	100%	0.0	96.41	3103
KF668184.1	complete cds	Redspotted grouper nervous necrosis virus	1277	1277	100%	0.0	96.28	3024
GQ402012.1	complete cds	Redspotted grouper nervous necrosis virus	1277	1277	100%	0.0	96.28	2998
MN309751.1	complete sequence	Hulong grouper nervous necrosis virus	1271	1271	100%	0.0	96.15	3103
KY785170.1	partial cds	Sparus aurata betanodavirus	1269	1269	100%	0.0	96.02	2826
MT157513.1	complete cds	Pearl gentian grouper nervous necrosis virus	1249	1249	100%	0.0	95.64	3103
AY369136.1	complete sequence	Redspotted grouper nervous necrosis virus	1249	1249	100%	0.0	95.64	3103
MG600032.1	complete cds	Betanodavirus sp.	1238	1238	100%	0.0	95.38	3109
MN709776.1	complete cds	Redspotted grouper nervous necrosis virus	1232	1232	100%	0.0	95.25	3024
KY315688.1	complete sequence	Redspotted grouper nervous necrosis virus	1232	1232	100%	0.0	95.25	3095
HQ859906.1	complete cds	Tiger grouper Nervous Necrosis Virus	1232	1232	100%	0.0	95.25	3024
HQ859903.1	complete cds	Asian seabass Nervous Necrosis Virus	1232	1232	100%	0.0	95.25	3024

HQ859900.1	complete cds	Golden pompano nervous necrosis virus	1232	1232	100%	0.0	95.25	3024
HQ859898.1	complete cds	Asian seabass Nervous Necrosis Virus	1227	1227	100%	0.0	95.12	3024

**Table S4. BLASTn top resulting records similar to the RNA2 sequence from the NNV isolated in this work.**								
**Accession**	**Coverage**	**Scientific Name**	**Max Score**	**Total Score**	**Query Cover**	**E value**	**Per. ident**	**Acc. Len**
JN189992.2	complete cds	Dicentrarchus labrax betanodavirus	1799	1799	100%	0.0	99.59	1433
MN709777.1	complete cds	Redspotted grouper nervous necrosis virus	1600	1600	100%	0.0	95.95	1362
HQ859938.1	complete cds	Tiger grouper Nervous Necrosis Virus	1594	1594	100%	0.0	95.85	1363
HQ859935.1	complete cds	Asian seabass Nervous Necrosis Virus	1589	1589	100%	0.0	95.74	1363
HQ859945.1	complete cds	Tiger grouper Nervous Necrosis Virus	1583	1583	100%	0.0	95.64	1363
HQ859924.1	complete cds	Mouse grouper Nervous Necrosis Virus	1583	1583	100%	0.0	95.64	1363
HQ859930.1	complete cds	Asian seabass Nervous Necrosis Virus	1583	1583	100%	0.0	95.65	1363
AY721615.1	complete cds	Dragon grouper nervous necrosis virus	1578	1578	100%	0.0	95.54	1433
MH758754.1	complete cds	Redspotted grouper nervous necrosis virus	1572	1572	100%	0.0	95.44	1358
GU826692.1	complete sequence	Lates calcarifer nervous necrosis virus	1572	1572	100%	0.0	95.44	1434
KC696562.1	complete cds	Redspotted grouper nervous necrosis virus	1567	1567	100%	0.0	95.34	1366
HQ859948.1	complete cds	Asian seabass Nervous Necrosis Virus	1567	1567	100%	0.0	95.34	1363
HM485328.1	complete sequence	Lates calcarifer nervous necrosis virus	1567	1567	100%	0.0	95.34	1434
AY324870.1	complete cds	Sevenband grouper nervous necrosis virus	1567	1567	100%	0.0	95.34	1434
AF281657.1	complete cds	Epinephelus tauvina nervous necrosis virus	1567	1567	100%	0.0	95.34	1368
MG637439.1	complete cds	Pearl gentian grouper nervous necrosis virus	1561	1561	100%	0.0	95.24	1432
HQ859927.1	complete cds	Tiger grouper Nervous Necrosis Virus	1561	1561	100%	0.0	95.24	1363
AB373029.1	complete sequence	Sevenband grouper nervous necrosis virus	1561	1561	100%	0.0	95.24	1433
AY690596.1	complete cds	Redspotted grouper nervous necrosis virus	1561	1561	100%	0.0	95.24	1430
MF510920.1	complete cds	Epinephelus coioides nervous necrosis virus	1555	1555	100%	0.0	95.14	1433
KY354694.1	complete cds	Sparus aurata betanodavirus	1555	1555	100%	0.0	95.14	1416
MW590702.1	complete sequence	Redspotted grouper nervous necrosis virus	1555	1555	100%	0.0	95.14	1316
GU826693.1	complete cds	Lates calcarifer nervous necrosis virus	1555	1555	100%	0.0	95.14	1089
MT157514.1	complete cds	Pearl gentian grouper nervous necrosis virus	1550	1550	100%	0.0	95.04	1434

Figure S1. Multiple sequence alignment of RNA1 sequences from the selected genotype representatives.

```
GB acc. no                                                                      bp

AY369136.1        TAACATCACCTTCTTGCTCTGTTGAGTCATCACTTACGCAAGGTTACCGTTCAGCTTAGA    60
KY315688.1        ---------CTTCTTGCTCTGTTGAGTAATCACTTACGCAAGGTTACCGTTCCGCTTAGA    51
JN189909.2        TAACATCACCTTCTTGCTTTGTTGAGTAATCACTTACGCAAGGTTACCGTTTAGCTTAGA    60
MT157513.1        TTACATCACCTTCTTGCTCTGTTGAGTAATCACTTACGCAAGGTTACCGTTCAGCTTAGA    60
JN189865.2        TAACATCACCTTCTTGCTCTGTTGAGTAATCACTTACGCAAGGTTACCGTTCAGCTTAGA    60
MF144242.1        TAACATCACCTTCTTGCTCTGTTGAGTAATCACTTACGCAAGGTTACCGTTCAGCTTAGA    60
AF319555.1        TAACATCACCTTCTTGCTCTGTTGAGTAATCACTTACGCAAGGTTACCGTACAGCTTAGA    60
FJ789783.1        TAACATCACCTTCTTGCTCTGTTGAGTAATCACTTACGCAAGGTTACCGTTCAGCTTAGA    60
NC_008040.1       TAACATCACCTTCTTGCTCTGTTGAGTAATCACTTACGCAAGGTTACCGTTTAGCTTAGA    60
EF558368.1        TAACATCACCTTCTTGCTCTGTTGAGTCATCACTTACGCAAGGTTACCGTTCAGCTTAGA    60
AY721616.1        TAACATCACCTTCTTGCTCTGTTGAGTAATCACTTACGCAAGGTTACCGTTCAGCTTAGA    60
KY354681.1        ---CATCACCTTCTTGCTCTGTTGAGTAATCACTTACGCAAGGTTACCGTTCAGCTTAGA    57
KP455643.1        TAACATCACCTTCTTGCTCTGTTGAGTAATCACTTACGCAAGGTTACCGTTCAGCTTAGA    60
MN309751.1        TAACATCACCTTCTTGCTCTGTTGAGTAATCACTTACGCAAGGTTACCGTTCAGCTTAGA    60
                           ********* ******** **********************   *******

AY369136.1        CAACGACAAGTCTACGCCATGCGTCGCTTTGAGTTCGCACTCGCACGCATGTCTGGAGCA    120
KY315688.1        CAACGACAAGTCTAGGCCATGCGTCGCATTGAGTCCGCACTCGCACGCATGACTGGAGCA    111
JN189909.2        CAACGACAAGTCTACGCCATGCGTCGCTTTGAGTTCGCACTCGCACGCATGTCTGGAGCA    120
MT157513.1        CA-AGACAAGTCTACGCCATGCGTCGCTTTGAGTTTGCACTCGCACGCATGTCTGGAGCA    119
JN189865.2        CAACGACAAGTCTACGCCATGCGTCGCTTTGAGTTTGCACTCGCACGCATGTCTGGAGCA    120
MF144242.1        CAACGACAAGTCTACGCCATGCGTCGCTTTGAGTTCGCACTCGCACGCATGTCTGGAGCA    120
AF319555.1        CAACGACAAGTCTACGCCATGCGTCGCTTTGAGTTTGCACTCGCACGCATGTCTGGAGCA    120
FJ789783.1        CAACGACAAGTCTACGCCATGCGTCGCTTTGAGTTTGCACTCGCACGCATGTCTGGAGCA    120
NC_008040.1       CAACGACAAGTCTACGCCATGCGTCGCTTTGAGTTTGCACTCGCACGCATGTCTGGAGCA    120
EF558368.1        CAACGACAAGTCTACGCCATGCGTCGCTTTGAGTTTGCACTCGCACGCATGTCTGGAGCA    120
AY721616.1        CAACGACAAGTCTACGCCATGCGTCGCTTTGAGTTTGCACTCGCACGCATGTCTGGAGCA    120
KY354681.1        CAACGACAAGTCTACGCCATGCGTCGCTTTGAGTTTGCACTCGCACGCATGTCTGGAGCA    117
KP455643.1        CAACGACAAGTCTACGCCATGCGTCGCTTTGAGTTTGCACTCGCACGCATGTCTGGAGCA    120
MN309751.1        CAACGACAAGTCTACGCCATGCGTCGCTTTGAGTTTGCACTCGCACGCATGTCTGGAGCA    120
                  **  ********** ************ ******  *************** ********

AY369136.1        GCATTTTGTGTGTACACAGGCTACCGCCTGTTGACCTCAAAATGGCTCGCGGACCGTGTT    180
KY315688.1        GCATTTTGTGTGTACACAGGCTACCGCCTGTTGACCTCAAAATGGCTCGCGGACCGTGTT    171
JN189909.2        GCATTTTGTGTGTACACAGGCTACCGCCTGTTGACCTCAAAATGGCTCGCGGACCGTGTT    180
MT157513.1        GCATTTTGTGTGTACACAGGCTACCGCCTGTTGACCTCAAAATGGCTCGCGGACCGTGTT    179
JN189865.2        GCATTTTGTGTGTACACAGGCTATCGCCTGTTGACCTCAAAATGGCTCGCGGACCGTGTC    180
MF144242.1        GCATTTTGTGTGTACACAGGCTACCGCCTGTTGACCTCAAAATGGCTCGCGGACCGTGTT    180
AF319555.1        GCATTTTGTGTGTACACAGGCTACCGCCTGTTGACCTCAAAATGGCTCGCGGACCGTGTT    180
FJ789783.1        GCATTTTGTGTGTACACAGGCTATCGCCTGTTGACCTCAAAATGGCTCGCGGACCGTGTC    180
NC_008040.1       GCATTTTGTGTGTACACAGGCTACCGCCTGTTGACCTCAAAATGGCTCGCGGACCGTGTT    180
EF558368.1        GCATTTTGTGTGTACACAGGCTACCGCCTGTTGACCTCAAAATGGCTCGCGGACCGTGTT    180
AY721616.1        GCATTTTGTGTGTACACAGGCTACCGCCTGTTGACCTCAAAATGGCTCGCGGACCGTGTT    180
KY354681.1        GCATTTTGTGTGTACACAGGCTACCGCCTGTTGACCTCAAAATGGCTCGCGGACCGTGTT    177
KP455643.1        GCATTTTGTGTGTACACAGGCTACCGCCTGTTGACCTCAAAATGGCTCGCGGACCGTGTT    180
MN309751.1        GCATTTTGTGTGTACACAGGCTACCGCCTGTTGACCTCAAAATGGCTCGCGGACCGTGTT    180
                  *********************** ***********************************

AY369136.1        GAGGATTATCGCCAACGCATCATCGCTGAGAAGAAACAAGTTCTCCGTGATGCGGCCATG    240
KY315688.1        GAGGATTATCGCCATCGCATCATCGCTGAGAAGAAACAAGTTCTCCGTGATGCGGCCATG    231
JN189909.2        GAGGATTATCGCCAACGCATCATCGCTGAGAAGAAACAAGTTCTCCGTGATGCGGCCATG    240
MT157513.1        GAGGATTATCGCCAACGCATCATCGCTGAGAAGAAACAAGTTCTCCGTGATGCGGCCATG    239
JN189865.2        GAGGATTATCGCCAACGCGTCATTGCTGAGAAGAAACAAATTCTCCGTGATGCGGCCATG    240
MF144242.1        GAGGATTATCGCCAACGCGTCATCGCTGAGAAGAAACAAATTCTCCGTGACGCGGCCATG    240
AF319555.1        GAGGATTATCGCCAACGCATCATCGCTGAGAAGAAACAAATTCTCCGTGATGCGGCCATG    240
FJ789783.1        GAGGATTATCGCCAACGCGTCATTGCTGAGAAGAAACAAATTCTCCGTGATGCGGCCATG    240
NC_008040.1       GAGGATTATCGCCAACGCGTCATCGCTGAGAAGAAACAAATTCTCCGTGATGCGGCCATG    240
EF558368.1        GAGGATTATCGCCAACGCGTCACCGCTGAGAAGAAACAAATTCTCCGTGATGCGGCCATG    240
AY721616.1        GAGGATTATCGCCAACGCGTCATCGCTGAGAAGAAACAAATTCTCCGTGACGCGGCCATG    240
KY354681.1        GAGGATTATCGCCAACGCGTCATCGCTGAGAAGAAACAAATTCTCCGTGACGCGGCCATG    237
KP455643.1        GAGGATTATCGCCAACGCGTCATCGCTGAGAAGAAACAAATTCTCCGTGACGCGGCCATG    240
MN309751.1        GAGGATTATCGCCAACGCGTCATCGCTGAGAAGAAACAAATTCTCCGTGACGCGGCCATG    240
                  ************** *** ***  *************** ********** *********

AY369136.1        ATCCGGACTCAGATCCAGCGGGAAATGGAATTGGTGCGCATCTCGGTGCGCAAAGGCCAT    300
KY315688.1        ATCCGGACTCAGATCCAGCGGGAAATGGAATTGGTGCGCATTTCGGTGCGCAAAGGCCAT    291
JN189909.2        ATCCGGACTCAGATCCAGCGGGAAATGGAATTGGTGCGCATCTCGGTGCGCAAAGGCCAT    300
MT157513.1        ATCCGGACTCAGATCCAGCGGGAAATGGAGCTGGTGCGCATCTCGGTGCGCAAAGGCCAT    299
JN189865.2        ATCCGGACTCAGATCCAGCGGGAAATGGAACTGGTACGCATTTCGGTGCGTAAAGGCCAT    300
MF144242.1        ATCCGGACTCAGATCCAGCGGGAAATGGAGCTAGTGCGCATTTCGGTGCGCAAAGGCCAT    300
AF319555.1        ATCCGTACTCAGATCCAGCGGGAAATGGAGCTGGTGCGCATCTCGGTGCGCAAAGGCCAT    300
FJ789783.1        ATCCGGACTCAGATCCAGCGGGAAATGGAACTGGTGCGCATTTCGGTGCGCAAAGGCCAT    300
```

```
NC_008040.1      ATCCGGACTCAGATCCAGCGGGAAATGGAACTGGTGCGTATCTCGGTGCGCAAAGGCCAT     300
EF558368.1       ATCCGGACTCAGATCCAGCGGGAAATGGAACTGGTGCGTATCTCGGTGCGCAAGGGCCAT     300
AY721616.1       ATCCGGACTCAGATCCAGCGGGAAATGGAGCTAGTGCGTATCTCGGTGCGCAAAGGCCAT     300
KY354681.1       ATCCGGACTCAGATCCAGCGGGAAATGGAGCTAGTGCGCATTTCGGTGCGCAAAGGCCAT     297
KP455643.1       ATCCGGACTCAGATCCAGCGGGAAATGGAGCTAGTGCGCATTTCGGTGCGCAAAGGCCAT     300
MN309751.1       ATCCGGACCCAGATCCAGCGGGAAATGGAGCTAGTGCGCATCTCGGTGCGCAAAGGCCAT     300
                 ***** ** ********************  * ** ** ** ******** ** ******

AY369136.1       TCCCATCAGGAAGCTGCTACTGAGCGTAACAGCGCCACTGAGACCATGCTCGGTGTGGTG     360
KY315688.1       TCCCATCAGGAAGCTGCTACTGAGCGTAACAGCGCCACTGAGACCATGCTCGGTGTGGTG     351
JN189909.2       TCCCATCAGGAAGCTGCTACTGAGCGTAACAGCGCCACTGAGACCATGCTCGGTGTGGTG     360
MT157513.1       TCCCACCAGGAAGCTGCTACTGAGCGTAACAGCGCCACTGAAACCATGCTCGGTGTGGTG     359
JN189865.2       TCCCACCAGGAAGCTGCTACTGAGCGTAACAGCGCCACTGAGACCATGCTCGGTGTGGTG     360
MF144242.1       TCCCATCAGGAAGCCGCTACTGAGCGTAACAGCGCTACTGAGACCATGCTCGGTGTGGTG     360
AF319555.1       TCCCACCAGGAAGCTGCTACTGAGCGTAACAGCGCCACTGAGACCATGCTCGGTGTGGTG     360
FJ789783.1       TCCCATCAGGAAGCCGCTACTGAGCGTAACAGCGCTACTGAGACCATGCTCGGTGTGGTG     360
NC_008040.1      TCCCATCAGGAAGCCGCTACTGAGCGCAACAGCGCTACTGAGACCATGCTCGGTGTGGTG     360
EF558368.1       TCCCATCAGGAAGCCGCTACTGAGCGTAACAGCGCTACTGAGACCATGCTCGGTGTGGTG     360
AY721616.1       TCCCATCAGGAAGCCGCTACTGAGCGTAACAGCGCCACTGAGACCATGCTCGGTGTGGTG     360
KY354681.1       TCCCATCAGGAAGCCGCTACTGAGCGTAACAGCGCTACTGAGACCATGCTCGGTGTGGTG     357
KP455643.1       TCCCATCAGGAAGCCGCTACTGAGCGTAACAGCGCTACTGAGACCATGCTCGGTGTGGTG     360
MN309751.1       TCCCATCAGGAAGCCGCTACTGAGCGTAACAGCGCTACTGAGACCATGCTCGGTGTGGTG     360
                 ***** ******** *********** ******** ***** ******************

AY369136.1       GAGAAATGTGGCTATGAGCCATATGTCATTTCACCATCACCCCGTGAGGTTGGATACCAC     420
KY315688.1       GAGAAATGTGGCTATGAGCCATATGTCATTTCACCATCAACCCGTGAGGTTGGATACCAC     411
JN189909.2       GAGAAATGTGGCTATGAGCCATATGTCATTTCACCATCACCCCGTGAGGTTGGATACCAC     420
MT157513.1       GAGAAATGTGGCTACGAGCCATATGTTATCTCTCCATCACCCCGTGAGGTTGGATACCAC     419
JN189865.2       GAGAAATGCGGCTACGAGCCATATGTCATTTCACCATCACCCCGTGAGGTTGGATACCAC     420
MF144242.1       GAGAAATGTGGCTACGAGCCATATGTCATTTCACCATCACCCCGTGAGGTTGGATACCAC     420
AF319555.1       GAGAAATGTGGCTACGAGCCATATGTCATTTCTCCATCACCCCGTGAGGTTGGATACCAC     420
FJ789783.1       GAGAAATGTGGCTACGAGCCATATGTCATTTCACCATCACCCCGTGAGGTTGGATACCAC     420
NC_008040.1      GAGAAATGTGGCTACGAGCCATATGTCATTTCACCATCACCCCGTGAGGTTGGATACCAC     420
EF558368.1       GAGAAATGTGGCTACGAGCCATATGTCATTTCACCATCACCCCGTGAGGTTGGATACCAC     420
AY721616.1       GAGAAATGTGGCTACGAGCCATATGTCATTTCACCATCACCCCGTGAGGTTGGATACCAC     420
KY354681.1       GAGAAATGTGGCTACGAGCCATATGTCATTTCACCATCACCCCGTGAGGTTGGATATCAC     417
KP455643.1       GAGAAATGTGGCTACGAGCCATACGTCATTTCACCATCACCCCGTGAGGTTGGATACCAC     420
MN309751.1       GAGAAATGTGGCTACGAGCCATATGTCATTTCACCATCACCCCGTGAGGTTGGATACCAC     420
                 ******** ***** ******** ** ** ** ****** **************** ***

AY369136.1       GGGTCACGTCAGTTCTACAGTCTGGCGGATTTCCGCCAAGACTACCGTCGCGATGACATC     480
KY315688.1       GGGTCACGTCAGTTCTACAGTCTGGCGGATTTCCGCCAAGACTACCGTCGTGATGACATC     471
JN189909.2       GGGTCACGTCAGTTCTATAGTCTGGCAGATTTTCGCCAAGACTACCGTCGTGATGACATC     480
MT157513.1       GGGTCACGTCAGTTCTATAGTCTGGCAGATTTTCGCCAAGACTACCGTCGTGATGACATC     479
JN189865.2       GGGTCACGTCAATTCTATAGTCTGGCAGACTTTCGCCAAGACTACCGTCGTGATGACATC     480
MF144242.1       GGGTCACGTCAGTTCTATAGTCTGGCAGACTTTCGCCAAGACTACCGTCGTGATGACATC     480
AF319555.1       GGGTCACGTCAGTTCTATAGTCTGGCAGATTTTCGCCAAGACTACCGTCGTGATGACATC     480
FJ789783.1       GGGTCACGTCAGTTCTATAGTCTGGCGGATTTTCGCCAAGACTATCGTCGTGACGACATC     480
NC_008040.1      GGGTCACGTCAGTTCTATAGTCTGGCAGATTTTCGCCAAGACTACCGTCGTGATGACATC     480
EF558368.1       GGGTCACGTCAGTTTTATAGTCTGGCGGATTTTCGCCAAGACTACCGTCGTGATGACATC     480
AY721616.1       GGGTCACGTCAGTTCTATAGTCTGGCAGATTTTCGCCAAGACTACCGTCGTGATGACATC     480
KY354681.1       GGGTCACGTCAGTTCTATAGTCTGGCAGATTTTCGCCAAGACTACCGTCGTGATGACATC     477
KP455643.1       GGGTCACGTCAGTTCTATAGTCTGGCAGACTTTCGCCAAGACTACCGTCGTGATGACATC     480
MN309751.1       GGGTCACGTCAGTTCTATAGTCTGGCAGATTTTCGCCAAGACTACCGTCGTGATGACATC     480
                 *********** ** ** ******** ** ** *********** ***** ** ******

AY369136.1       ACCGACCGTCACATCATTGTGATGACTGATGTTGACTACTACGTGGACATGCATGAGTTG     540
KY315688.1       ACCGACCGTCACATCATTGTGATGACTGATGTTGACTATTACGTGGACATGCATGAGTTG     531
JN189909.2       ACCGACCGTCACATCATTGTGATGACTGATGTTGACTACTACGTGGACATGCATGAGTTG     540
MT157513.1       ACCGACCGCCACATCATTGTGATGACTGACGTTGACTACTACGTGGACATGCATGAGTTG     539
JN189865.2       ACCGACCGTCACATCATTGTGATGACTGATGTTGACTACTACGTGGACATGCATGAGTTG     540
MF144242.1       ACCGACCGTCACATCATTGTGATGACTGATGTTGACTACTACGTGGACATGCATGAGTTG     540
AF319555.1       ACCGACCGTCACATCATTGTGATGACTGATGTTGACTACTACGTGGACATGCATGAGTTG     540
FJ789783.1       ACCGATCGTCACATCATTGTGATGACTGATGTTGACTACTACGTGGACATGCATGAGTTA     540
NC_008040.1      ACCGACCGTCACATCATTGTGATGACTGATGTTGACTACTACGTGGACATGCATGAGTTG     540
EF558368.1       ACCGACCGTCACATCATTGTGATGACTGATGTTGACTACTACGTGGACATGCATGAGTTG     540
AY721616.1       ACCGACCGTCACATCATTGTGATGACTGATGTTGACTACTACGTGGACATGCATGAGTTG     540
KY354681.1       ACCGACCGTCACATCATTGTGATGACTGATGTTGACTACTACGTGGACATGCATGAGTTG     537
KP455643.1       ACCGACCGTCACATTATTGTGATGACTGATGTTGACTACTACGTGGACATGCATGAGTTG     540
MN309751.1       ACCGACCGTCACATCATTGTGATGACTGATGTTGACTACTACGTGGACATGCATGAGTTG     540
                 ***** ** ***** ************** ******** ******************** 

AY369136.1       ATTGGTTTGGGTGTTCCGATATTGTTGTACACCTTCCAGCCAAGTACTGTGTCCGGAGAA     600
KY315688.1       ATTGGATTGGGTGTTCCGATATTGTTGTACACCTTCCAGCCCAGTACTGTGTCTGGAGAA     591
JN189909.2       ATTGGTCTGGGTGTTCCGATATTACTGTACACCTTCCAGCCAAGTACTGTGTCCGGAGAG     600
MT157513.1       ATTGGTTTGGGTGTTCCGATATTGCTGTACACCTTCCAGCCAAGTACTGTGTCCGGAGAG     599
JN189865.2       ATTGGTTTGGGCGTTCCGATATTGCTGTACACCTTCCAGCCAAGTACTGTGTCCGGGGAG     600
```

```
MF144242.1        ATTGGTTTGGGTGTTCCGATACTGCTATACACCTTCCAGCCAAGTACTGTGTCCGGAGAG    600
AF319555.1        ATTGGTTTGGGTGTTCCGATATTGTTGTACACCTTCCAGCCAAGTACTGTGTCCGGAGAG    600
FJ789783.1        ATTGGTTTGGGTGTTCCAATATTGCTGTACACCTTCCAGCCAAGTACTGTGTCCGGAGAG    600
NC_008040.1       ATTGGTTTGGGTGTTCCGATATTGCTGTACACCTTCCAGCCAAGTACTGTGTCCGGAGAG    600
EF558368.1        ATTGGTTTGGGTGTTCCGATATTGCTGTACACCTTCCAGCCAAGTACTGTGTCCGGAGAG    600
AY721616.1        ATTGGTTTGGGTGTTCCGATACTGCTGTACACCTTCCAGCCAAGTACTGTGTCCGGAGAG    600
KY354681.1        ATTGGTTTGGGTGTTCCGATACTGCTATACACCTTCCAGCCAAGTACTGTGTCCGGAGAG    597
KP455643.1        ATTGGTTTGGGTGTTCCGATACTGCTATACACCTTCCAGCCAAGTACTGTGTCCGGAGAG    600
MN309751.1        ATTGGTTTGGGTGTTCCGATACTGCTATACACCTTCCAGCCAAGTACTGTGTCCGGAGAG    600
                  *****  **** ***** *** *  * ************** *********** ** **

AY369136.1        GTTAAGGATGGTTACTTCACCATCACTGACGACTCCGTTCACTACCGTGTTGCTGGTGGG    660
KY315688.1        GTTAAGGATGGTTATTTCACCATCACTGACGACTCCGTTCACTACCGTGTTGCTGGTGGG    651
JN189909.2        GTTAAGGATGGTTACTTTACCATCACTGACGACTCCGTTCACTACCGTGTTGCTGGCGGG    660
MT157513.1        GTTAAGGATGGTTACTTTACCATCACTGACGACTCCGTTCACTACCGTGTTGCTGGCGGG    659
JN189865.2        GTCAAGGATGGTTATTTTACCATCACTGACGACTCCGTTCACTACCGTGTTGCTGGCGGG    660
MF144242.1        GTTAAGGATGGTTACTTTACCATCACTGACGACTCCGTTCACTACCGTGTTGCTGGCGGG    660
AF319555.1        GTTAAGGATGGTTACTTTACCATCACTGACGACTCCGTTCACTACCGTGTTGCTGGCGGG    660
FJ789783.1        GTTAAGGATGGTTACTTTACCATCACTGACGACTCCGTTCACTACCGTGTTGCTGGCGGG    660
NC_008040.1       GTTAAGGATGGTTACTTTACCATCACTGACGACTCCGTTCACTACCGTGTTGCTGGCGGG    660
EF558368.1        GTTAAGGATGGTTACTTCACCATCACTGACGACTCCGTTCACTACCGTGTTGCTGGCGGG    660
AY721616.1        GTTAAGGATGGTTACTTTACCATCACTGACGACTCCGTTCACTACCGTGTTGCTGGCGGG    660
KY354681.1        GTTAAGGATGGTTACTTTACCATCACTGACGACTCCGTTCACTACCGTGTTGCTGGCGGG    657
KP455643.1        GTTAAGGATGGTTACTTTACCATCACTGACGACTCCGTTCACTACCGTGTTGCTGGCGGG    660
MN309751.1        GTTAAGGATGGTTACTTTACCATCACTGACGACTCCGTTCACTACCGTGTTGCTGGCGGG    660
                  ** *********** ** ************************************** ***

AY369136.1        AAGGATGTGCGCCACCGCATTTGGAACTACAACCAAGATACCATGTATGTGTGCTCCCGA    720
KY315688.1        AAGGATGTGCGCCACCGCATTTGGAACTACAACCAAGATACCATGTATGTGTGCTCCCGA    711
JN189909.2        AAGGATGTGCGCCATCGCATCTGGAACTACAACCAGGATACCATGTATGTGTGCTCCCGA    720
MT157513.1        AAGGATGTGCGTCATCGCATCTGGAACTACAACCAAGACACCATGTATGTCTGCTCCCGA    719
JN189865.2        AAGGATGTGCGCCATCGCATTTGGAACTACAACCAGGATACCATGTATGTGTGCTCCCGA    720
MF144242.1        AAGGATGTGCGCCATCGCATCTGGAACTACAACCAGGATACCATGTATGTGTGCTCCCGA    720
AF319555.1        AAGGATGTGCGCCACCGCATCTGGAACTACAACCATGATACCATGTATGTGTGCTCCCGA    720
FJ789783.1        AAGGATGTGCGCCATCGCATCTGGAACTACAACCAGGATACCATGTATGTGTGCTCCCGA    720
NC_008040.1       AAGGATGTGCGCCATCGCATCTGGAACTACAACCAGGATACCATGTATGTGTGCTCCCGA    720
EF558368.1        AAGGATGTGCGCCATCGCATCTGGAACTACAACCAGGATACCATGTATGTGTGCTCCCGA    720
AY721616.1        AAGGATGTGCGCCATCGCATCTGGAACTACAACCAGGATACCATGTATGTGTGCTCCCGA    720
KY354681.1        AAGGATGTGCGCCATCGCATCTGGAACTACAACCAGGATACCATGTATGTGTGCTCCCGA    717
KP455643.1        AAGGATGTGCGCCATCGCATCTGGAACTACAACCAGGATACCATGTATGTGTGCTCCCGA    720
MN309751.1        AAGGATGTGCGCCATCGCATCTGGAACTACAACCAGGATACCATGTATGTGTGCTCCCGA    720
                  *********** ** ***** ************** ** *********** *********

AY369136.1        CCTCGTGGTTTCTGGGCGAATCTGGTGCAGATTCTACGTGACATCACTGGTGTCACCGCG    780
KY315688.1        CCTCGTGGTTTCTGGGCGAATCTGGCGCGGATCCTGCGTGACATCACTGGTGTCACCGCG    771
JN189909.2        CCTCGCGGTCTCTGGGCGAATCTGTTGCAGATCCTGCGTGACATCACTGGTGTCACCGCG    780
MT157513.1        CCTCGTGGTTTCTGGGCGAATCTGATGCAGATCCTGCGCGACATCACTGGTGTCACTGCA    779
JN189865.2        CCTCGTGGTTTCTGGGCGAATCTGATGCAGATCTTGCGTGACATCACTGGTGTCACCGCG    780
MF144242.1        CCTCGTGGTTTCTGGGCGAATCTGATGCAGATCTTGCGTGACATCACTGGTGTCACCGCG    780
AF319555.1        CCTCGTGGTTTCTGGGCGAATCTGATGCAGATTCTGCGTGACATCACTGGTGTCACCGCG    780
FJ789783.1        CCTCGTGGTTTCTGGGCGAATCTGATGCAGATCCTGCGTGATATCACTGGTGTCACCGCG    780
NC_008040.1       CCTCGTGGTTTCTGGGCGAATTTGATGCAGATCCTGCGTGACATCACTGGTGTCACCGCG    780
EF558368.1        CCTCGTGGTTTCTGGGCGAATCTGATGCAGATCCTGCGTGATATCACTGGTGTCACCGCG    780
AY721616.1        CCTCGTGGTTTCTGGGCGAATCTGATGCAGATCCTGCGTGATATCACTGGTATCACCGCG    780
KY354681.1        CCTCGTGGTTTCTGGGCGAATCTGATGCAGATCTTGCGTGACATCACTGGTGTCACCGCG    777
KP455643.1        CCTCGTGGTTTCTGGGCGAATCTGATGCAGATCTTGCGTGACATCACTGGTGTCACCGCG    780
MN309751.1        CCTCGTGGTTTCTGGGCGAATCTGATGCAGATCTTGCGTGACATCACTGGTGTCACTGCG    780
                  ***** *** *********** **  ** ***  * ** ** ********* **** **

AY369136.1        ATCTGTGGTTTCCTTTACGCCAAGCTCGGTATTGCGCCCTTCGGCGACCCTGTTACCATG    840
KY315688.1        ATCTGTGGTTTCCTTTACGCCAAGCTCGGTATTGCGCCCTTCGGCGACCCTGTTACCATG    831
JN189909.2        ATTTGTAGTTATCTTTACACCAAGCTCGGTATTGCGCCCTTCGGCGACCCTGTTACCATG    840
MT157513.1        ATCTGCAGCTTTCTTTACACCAAGCTCGGTATTGCGCCCTTTGGCGACCCTGTTACCATG    839
JN189865.2        ATCTGTAGCTTTCTTTACACCAAGCTCGGTATTGCGCCCTTCGGCGACCCTGTTACCATG    840
MF144242.1        ATCTGTAGCTTTCTTTACGCCAAGCTCGGTATTGCGCCCTTCGGCGACCCTGTTACCATG    840
AF319555.1        ATCTGTAGCTTTCTTTACACCAAGCTCGGTATTGCGCCCTTTGGCGACCCTGTTACCATG    840
FJ789783.1        ATCTGTAGCTTTCTCTACGCCAAGCTCGGTATTGCGCCCTTCGGCAACCCTGTCACCATG    840
NC_008040.1       ATCTGTAGCTTTCTTTACACCAAGCTCGGTATTGCGCCTTTCGGCGACCCTGTTACCATG    840
EF558368.1        ATCTGTAGTTTTCTTTATACCAAGCTCGGTATTGCGCCCTTCGGCGACCCTGTTACCATG    840
AY721616.1        ATCTGTAGCTTTCTTTACACCAAGCTCGGTATTGCGCCCTTCGGCGACCCTGTTACCATG    840
KY354681.1        ATCTGTAGCTTTCTATACGCCAAGCTCGGTATTGCGCCCTTCGGCGACCCTGTTACCATG    837
KP455643.1        ATCTGTAGCTTTCTTTACGCCAAGCTCGGTATTGCGCCCTTCGGCGACCCTGTTACCATG    840
MN309751.1        ATCTGTAGCTTTCTTTACGCCAAGCTCGGTATTGCGCCCTTCGGCGACCCTGTCACCATG    840
                  ** **  * *  ** **  ******************* ** *** ******* ******

AY369136.1        TTCACCGTCGATCAATTCAAGATGGGTGAGCACCGTAACATCGTGTCGATTGTGCCCTTT    900
KY315688.1        TTCACCGTCGATCAATTTAGGATGGGTGAGCACCGTAACATCGTGTCGATTGTGCCCTTT    891
```

```
JN189909.2      TTCACCGTTGATCAATTCAAGATGGGTGAGCACCGTAATATCGTGTCGATTGTGCCCTTT    900
MT157513.1      TTCACTGTTGATCAATTCAAGATGGGTGAGCATCGTAACATCGTGTCAATTGTGCCCTTT    899
JN189865.2      TTCACCGTTGATCAATTCAAGATGGGTGAGCATCGTAACATCGTGTCAATTGTGCCCTTT    900
MF144242.1      TTCACCGTTGATCAATTCAAGATCGGTGAGCATCGTAACATCGTCTCGATTGTGCCCTTT    900
AF319555.1      TTCACCGTTGATCAATTCAAGATGGGTGAGCATCGTAACATCGTGTCAATTGTGCCCTTT    900
FJ789783.1      TTCACCGTTGATCAATTCAAGATGGGTGAGCATCGTAACATCGTGTCGATTGTGCCCTTT    900
NC_008040.1     TTCACCGTTGATCAATTCAAGATGGGTGAGCATCGTAACATCGTGTCGATTGTGCCCTTT    900
EF558368.1      TTCACCGTTGATCAATTCAAGATGGGTGAGCATCGTAACATCGTGTCGATTGTGCCCTTT    900
AY721616.1      TTCACCGTTGATCAATTCAAGATGGGTGAGCATCGTAACATCGTGTCGATTGTGCCCTTT    900
KY354681.1      TTCACCGTCGATCAATTCAAGATGGGTGAGCATCGTAACATCGTGTCGATTGTGCCCTTT    897
KP455643.1      TTCACCGTTGATCAGTTCAAGATGGGTGAGCATCGTAACATCGTCTCGATTGTGCCCTTT    900
MN309751.1      TTCACCGTTGATCAATTCAAGATGGGTGAGCATCGTAACATCGTCTCGATTGTGCCCTTT    900
                ***** ** ***** ** * *** ******** ***** ***** ** ************

AY369136.1      GCAACTTGCCGTTCAAACCTGCTCAAGATCAGTGAGTATGGTGCTGAGTTGGAGTACATG    960
KY315688.1      GCAACTTGCCGTTCAAACCTGCTCAAGATCAGTGAGTATGGTGCTGAGTTGGAGTACATG    951
JN189909.2      GCAACTTGCCGTTCGAACCTGCTCAAGATCAGTGAGTATGGTACTGAGTTGGAGTATATG    960
MT157513.1      GCAACTTGCCGTTCAAACCTGCTCAAGATCAGTGAGTATGGTGCTGAGTTGGAGTATATG    959
JN189865.2      GCAACTTGCCGTTCAAACCTGCTCAAGATCAGTGAGTACGGTGCTGAGTTGGAGTATATG    960
MF144242.1      GCAACTTGCCGTTCAAACCTGCTCAAGATCAGTGAGTACGGTGCTGAGTTGGAGTATATG    960
AF319555.1      GCAACTTGCCGTTCAAACCTGCTCAAGATCAGTGAGTATGGTGCTGAGTTGGAGTATATG    960
FJ789783.1      GCAACTTGCCGTTCAAACCTGCTCAAGATCAGTGAGTACGGTGCTGAGTTGGAGTATATG    960
NC_008040.1     GCAACTTGCCGTTCAAATCTGCTCAAGATCAGTGAGTACGGTGCTGAGTTGGAGTATATG    960
EF558368.1      GCAACTTGCCGTTCAAACCTGCTCAAGATCAGTGAGTACGGTGCTGAGTTGGAGTATATG    960
AY721616.1      GCAACTTGCCGTTCAAACCTGCTCAAGATCAGTGAGTACGGTGCTGAGTTGGAGTACATG    960
KY354681.1      GCAACTTGCCGTTCAAACCTGCTCAAGATCAGTGAGTACGGTGCTGAGTTGGAGTATATG    957
KP455643.1      GCAACTTGCCGTTCAAACCTGCTCAAGATCAGTGAGTACGGTGCTGAGTTGGAGTATATG    960
MN309751.1      GCAACTTGCCGTTCAAACCTGCTCAAAATCAGTGAGTACGGTGCTGAGTTGGAGTACATG    960
                ************** ** ******** *********** *** ************* ***

AY369136.1      CGCTACCAGCAGCGCAACAACATTGCCAACTTCAACGCAGTCACTTACATCTCTGAGAAT    1020
KY315688.1      CGCTATCAACAGCGCAACAACATTGCCAACTTCAACGCAGTCACTTACATCTCTGAGAAT    1011
JN189909.2      CGCTACCAACAGCGCAACAACATTGCCAACTTCAACGCAGTCACTTACATCTCTGAGAAT    1020
MT157513.1      CGCTATCAACAGCGCAACAACATTGCCAACTTCAACGCAGTCACTTATATCTCTGAGAAT    1019
JN189865.2      CGCTACCAGCAGCGCAACAACATTGCCAACTTCAACGCAGTCACCTACATCTCTGAGAAT    1020
MF144242.1      CGCTACCAACAGCGCAACAACATTGCCAACTTCAACGCAGTCACTTATATCTCTGAGAAT    1020
AF319555.1      CGCTACCAACAGCGCAACAACATTGCCAACTTCAACGCAGTCACTTACATCTCTGAGAAT    1020
FJ789783.1      CGCTACCAACAGCGCAACAACATTGCCAACTTCAACGCAGTCACTTACATCTCTGAGAAT    1020
NC_008040.1     CGCTACCAACAGCGCAACAACATTGCCAACTTCAACGCAGTCACTTACATCTCTGAGAAT    1020
EF558368.1      CGCTACCAACAGCGCAACAACATTGCCAACTTCAACGCAGTCACTTACATCTCTGAGAAT    1020
AY721616.1      CGCTATCAACAGCGCAACAACATTGCCAACTTCAACGCAGTCACTTACATCTCTGAGAAT    1020
KY354681.1      CGCTACCAACAGCGCAACAACATTGCCAACTTCAACGCAGTCACTTATATCTCTGAGAAT    1017
KP455643.1      CGCTACCAACAGCGCAACAACATTGCCAACTTCAACGCAGTCACTTATATCTCTGAGAAT    1020
MN309751.1      CGCTACCAACAGCGCAACAATATTGCCAACTTCAACGCAGTCACTTATATCTCTGAGAAT    1020
                ***** ** *********** *********************** ** ************

AY369136.1      GGGCCACTCATCAGTCTGGGTTTGGAAGGCAATTTTGCAAGCGTCCAGCTTCCCCTGCAA    1080
KY315688.1      GGGCCACTCATCAGTTTGGGTTTGGAAGGCAATTTTGCAAGCGTCCAGCTTCCCCTGCAG    1071
JN189909.2      GGGCCGCTCATCAGTCTGGGTTTGGAAGGCAATTTTGCAAGTGTCCAGCTTCCCCTGCAG    1080
MT157513.1      GGGCCACTCATCAGTCTGGGTTTGGAAGGCAATTTTGCAAGCGTCCAGCTTCCTCTGCAG    1079
JN189865.2      GGGCCACTCATCAGTCTGGGTTTGGAAGGCAACTTTGCAAGCGTCCAGCTTCCCCTGCAG    1080
MF144242.1      GGGCCCCTCATCAGCTTGGGTTTGGAAGGCAACTTTGCAAGCGTCCAGCTTCCCCTGCAG    1080
AF319555.1      GGGCCACTCATCAGTCTGGGTTTGGAAGGCAATTTTGCAAGCGTCCAACTTCCTCTGCAG    1080
FJ789783.1      GGGCCACTTATCAGCCTGGGTTTGGAAGGCAACTTTGCAAGCGTCCAGCTTCCCCTGCAG    1080
NC_008040.1     GGGCCACTCATCAGCTTGGGTTTGGAAGGCAACTTTGCAAGCGTCCAACTTCCTTTGCAG    1080
EF558368.1      GGGCCACTCATCAGCTTGGGTTTGGAAGGCAACTTTGCAAGCGTCCAGCTTCCCCTGCAG    1080
AY721616.1      GGGCCCCTCATCAGCTTGGGTTTGGAAGGCAACTTTGCAAGCGTCCAGCTTCCCCTGCAG    1080
KY354681.1      GGGCCCCTCATCAGCTTGGGTTTGGAAGGCAACTTTGCAAGTGTCCAGCTTCCCCTGCAG    1077
KP455643.1      GGGCCCCACATCAGCTTGGGTTTGGAAGGCAACTTCGCAAGCGTCCAGCTTCCCCTGCAG    1080
MN309751.1      GGGCCCCTCATCAGCTTGGGTTTGGAAGGCAACTTTGCAAGCGTCCAGCTTCCCCTGCAG    1080
                ***** *  *****  **************** ** ***** ***** *****  ****

AY369136.1      GATTTCGAGAACATCCGCACTGCATACGAACTGTCCAAGACTAACAACTTGTCAGACACT    1140
KY315688.1      GATTTCGAGAACATCCGCACTGCATACGAACTGTCCAAGACTAACAACTTGTCAGATACT    1131
JN189909.2      GATTTCGAGAACATCCGCACTGCATATGAACTGTCCAAAACTAACAACTTGTCAGATACT    1140
MT157513.1      GATTTCGAGAACATCCGCACTGCATACGAACTGTCCAAAACTAACAACTTGTCAGATACT    1139
JN189865.2      GATTTCGAGAACATCCGCACTGCATACGAACTGTCCAAAACTAACAACTTGTCAGATACT    1140
MF144242.1      GATTTTGAGAACATCCGCACTGCATACGAACTGTCCAAAACTAACAACTTGTCAGATACT    1140
AF319555.1      GATTTCGAGAACATCCGCACTGCATACGAACTGTCCAAAACTAACAACTTGTCAGATACT    1140
FJ789783.1      GATTTCGAGAACATCCGCACTGCATACGAACTGTCCAAAACTAACAACTTGTCAGATACT    1140
NC_008040.1     GATTTCGAGAACATCCGCACTGCATACGAACTGTCCAAAACTAACAACTTGTCAGATACT    1140
EF558368.1      GATTTCGAGAACATCCGCACTGCATACGAACTGTCCAAAACTAACAACTTGTCAGATACT    1140
AY721616.1      GATTTTGAGAACATCCGCACTGCATACGAACTGTCCAAAACTAACAACTTGTCAGATACT    1140
KY354681.1      GATTTTGAGAACATCCGCACTGCATACGAACTGTCCAAGACTAACAACTTGTCAGATACT    1137
KP455643.1      GATTTTGAGAACATCCGTACTGCATACGAACTGTCCAAAACTAACAACTTGTCAGATACT    1140
MN309751.1      GATTTTGAGAACATCCGCACTGCATACGAACTGTCCAAAACTAACAACTTGTCAGATACT    1140
                ***** *********** ******** *********** ***************** ***
```

```
AY369136.1      GTTCGCCGGTCAGGGCGCCCGTGCAAGGAGGCAGCTATCATACATAAGTGTCTCCAAGCC    1200
KY315688.1      GTTCGCCGGTCAGGGCGCCCGTGCAAGGAGGCAGCTATCATACATAAGTGTCTCCAAGCC    1191
JN189909.2      GTTCGCCGGTCAGGACGCCCGTGCAAGGAGGCAGCTATCATCCACAAATGTCTCCAAGCT    1200
MT157513.1      GTTCGCCGGTCAGGGCGCCCGTGCAAGGAGGCTGCTATCATCCATAAATGTCTCCAGGCC    1199
JN189865.2      GTTCGCCGGTCAGGGCGCCCGTGCAAGGAGGCAGCTATCATCCATAAATGTCTCCAAGCC    1200
MF144242.1      GTTCGCCGGTCAGGGCGCCCGTGCAAGGAGGCAGCTATCATCCATAAATGTCTCCAAGCC    1200
AF319555.1      GTTCGCCGGTCAGGGCGCCCGTGCAAGGAGGCAGCTATCATCCATAAATGTCTCCAAGCC    1200
FJ789783.1      GTTCGCCGGTCAGGGCGCCCGTGCAAGGAGGCAGCTATCATCCATAAATGTCTCCAAGCC    1200
NC_008040.1     GTTCGCCGGTCAGGGCGCCCGTGCAAGGAGGCAGCTATCATCCATAAATGTCTCCAAGCC    1200
EF558368.1      GTTCGCCGGTCAGGGCGCCCGTGCAAGGAGGCAGCTATCATCCATAAATGTCTCCAAGCC    1200
AY721616.1      GTTCGCCGGTCAGGGCGCCCGTGCAAGGAGGCAGCTATCATCCACAAATGTCTCCAAGCC    1200
KY354681.1      GTTCGCCGGTCAGGGCGCCCGTGCAAGGAGGCAGCTATCATCCATAAATGTCTCCAAGCC    1197
KP455643.1      GTTCGCCGGTCAGGGCGCCCGTGCAAGGAGGCAGCTATCATCCATAAATGTCTCCAAGCC    1200
MN309751.1      GTTCGCCGGTCAGGGCGCCCGTGCAAGGAGGCAGCCATCATCCATAAATGTCTCCAAGCC    1200
                ************** ***************** ** ***** ** ** ******** **

AY369136.1      GAGTGTGCCGTCGTCAGCGAGGTCGTGCATAAACCTGGCGATCTCGCTCGCCATTACCAA    1260
KY315688.1      GAGTGTGCCGTCGTCAGCGAGGTCGTGCATAAACCTGGCGATCTCGCTCGCCATTACCAA    1251
JN189909.2      GAGTGTGTCGTCGTCAGCGAGGTCGTACATAAACCTGGCGATCTTGCTCGTCATTACCAA    1260
MT157513.1      GAGTGTGCCGTCGTCAGCGAGGTCGTGCATAAACCCGGCGATCTCGCTCGTCATTACCAA    1259
JN189865.2      GAGTGCGCCGTCGTCAGCGAGGTCGTGCATAAACCTGGCGATCTTGCTCGTCATTACCAA    1260
MF144242.1      GAGTGTGCCGTCGTCAGCGAGGTCGTGCATAAACCTGGCGATCTTGCTCGTCATTACCAA    1260
AF319555.1      GAGTGTGCCGTCGTCAGCGAGGTCGTGCATAAACCTGGCGATCTCGCTCGTCATTACCAA    1260
FJ789783.1      GAGTGTGCCGTCGTCAGCGAGGTCGTGCATAAACCTGGCGATCTTGCTCGTCACTACCAA    1260
NC_008040.1     GAGTGTGCCGTCGTCAGCGAGGTCGTGCATAAACCTGGCGATCTTGCTCGTCATTACCAA    1260
EF558368.1      GAGTGTGCCGTCGTCAGCGAGGTCGTACATAAACCTGGCGATCTTGCTCGTCATTACCAA    1260
AY721616.1      GAGTGTGCCGTCGTCAGCGAGGTCGTGCATAAACCTGGCGATCTTGCTCGTCATTACCAA    1260
KY354681.1      GAGTGTGCCGTCGTCAGCGAGGTCGTGCATAAACCTGGCGATCTTGCTCGTCACTACCAA    1257
KP455643.1      GAGTGTGCCGTCGTCAGCGAGGTCGTGCATAAACCTGGCGATCTTGCTCGTCATTACCAA    1260
MN309751.1      GAGTGTGCCGTCGTCAGCGAGGTCGTGCATAAACCTGGCGATCTTGCTCGTCATTACCAA    1260
                ***** * ****************** ******** ******** ***** ** ******

AY369136.1      GCAGTTGGTAGTGCCTACGACACTGATCCGGCCGAGCAGGGCAAGTGTTATGCTCGTGAG    1320
KY315688.1      GCAGTTGGTAGTGTCTACGACACTGATCCGGCCGAGCAGGGCAAGTGTTATGCTCGTGAG    1311
JN189909.2      GCAGTTGGTAGTGCCTACGACACTGATCCGGCCGAGCAGGGCAAGTGTTACGCTCGTGAG    1320
MT157513.1      GCAGTTGGTAGTGCCTACGACACTGACCCAGCCGAACAGGGCAAGTGTTACGCCCGTGAG    1319
JN189865.2      GCTGTTGGTAGTGCCTACGACACTGATCCAGCCGAGCAGGGCAAGTGTTATGCTCGTGAG    1320
MF144242.1      GCAGTTGGTAGTGCCTACGACACTGATCCAGCTGAGCAGGGCAAGTGTTACGCTCGTGAG    1320
AF319555.1      GCAGTTGGTAGTGCCTACGACACTGATCCAGCCGAGCAGGGCAAGTGTTACGCCCGTGAG    1320
FJ789783.1      GCAGTTGGTAGTGCCTACGACACTGATCCAGCTGAGCAGGGCAAGTGTTACGCTCGTGAG    1320
NC_008040.1     GCAGTTGGTAGTGCCTACGACACTGATCCAGCTGAGCAGGGCAAGTGTTACGCGCGTGAG    1320
EF558368.1      GCAGTTGGTAGTGCCTACGACACTGATCCAGCTGAGCAGGGCAAGTGTTATGCTCGTGAG    1320
AY721616.1      GCAGTTGGTAGTGCCTACGACACTGATCCAGCTGAGCAGGGCAAGTGTTATGCCCGTGAG    1320
KY354681.1      GCAGTTGGTAGTGCCTACGACACTGATCCAGCTGAGCAGGGCAAGTGTTACGCTCGTGAG    1317
KP455643.1      GCAGTTGGTAGTGCCTACGACACTGATCCAGCTGAGCAGGGCAAGTGTTACGCTCGTGAG    1320
MN309751.1      GCAGTTGGTAGTGCCTACGACACTGATCCAGCTGAGCAGGGCAAGTGTTACGCTCGTGAG    1320
                ** ********** ************ ** ** ** ************** ** ******

AY369136.1      TATGCTCCTGGACCGTTGACTCAAACTGCTGTATTTCCAAGTGAGTCACGTTCCAACGAG    1380
KY315688.1      TATGCTCCTGGACCGTTGACTCAAACTGCTGTATTTCCAAGTGAGTCACGTTCCAACGAG    1371
JN189909.2      TATGCTCCTGGACCGTTGACTCAAACTGCTGTATTTCCAAGTGAGTCGCGTTCCAACGAG    1380
MT157513.1      TATGCTCCCGGACCGTTGACTCAAACTGCTGTATTTCCAAGTGAGTCACGCTCCAACGAG    1379
JN189865.2      TATGCTCCTGGACCGTTGACTCAAACTGCTGTATTTCCAAGTGAGTCACGTTCCAACGAG    1380
MF144242.1      TACGCTCCTGGACCGTTGACTCAAACTACTGTATTTCCAAGTGAGTCACGTTCCAACGAG    1380
AF319555.1      TATGCTCCTGGACCGTTGACTCAAACTGCTGTATTTCCAAGTGAGTCACGTTCCAACGAG    1380
FJ789783.1      TATGCTCCTGGACCGTTGACTCAAACTGCTGTATTTCCAAGTGAGTCACGATCCAACGAG    1380
NC_008040.1     TATGCTCCTGGACCGTTGACTCAAACTGCTGTATTTCCAAGTGAGTCACGTTCCAACGAG    1380
EF558368.1      TATGCTCCTGGACCGTTGACTCAAACTGCTGTATTTCCAAGTGAGTCACGTTCCAACGAG    1380
AY721616.1      TACGCTCCTGGACCGTTGACTCAAACTGCTGTATTTCCAAGCGAGTCACGTTCCAACGAG    1380
KY354681.1      TACGCTCCTGGACCGTTGACTCAAACTGCTGTATTTCCAAGTGAGTCACGTTCCAACGAG    1377
KP455643.1      TACGCTCCTGGACCGTTGACTCAAACTGCTGTATTTCCAAGTGAGTCACGTTCCAACGAG    1380
MN309751.1      TACGCTCCTGGACCGTTGACTCAAACTGCTGTATTTCCAAGTGAGTCACGTTCCAACGAG    1380
                ** ***** ****************** ************* ***** ** *********

AY369136.1      CTTGCCACAATCGACGGTCGTATTGCTGGTCCGCAAGCCAAGGCAAAGAGCCGCGAGCAC    1440
KY315688.1      CTTGCCACAATTGACGGTCGTATTGCTGGTCCGCAAGCCAAGGCAAAGAGCCGCGAGCAC    1431
JN189909.2      CTTGCCACAATCGACGGTCGTATTGCTGGTCCGCAAGCTAAGGCAAAGAGCCGCGAGCAC    1440
MT157513.1      CTTGCCACGATTGACGGTCGTATTGCTGGTCCGCAAGCTAAGGCAAAGAGCCGCGAGCAC    1439
JN189865.2      CTTGCCACAATCGACGGTCGTATTGCTGGTCCGCAAGCCAAGGCAAAGAGCCGCGAGCAC    1440
MF144242.1      CTTGCCACAATCGACGGTCGTATTGCTGGTCCGCAAGCCAAGGCAAAGAGCCGCGAGCAC    1440
AF319555.1      CTTGCCACAATCGACGGTCGTATTGCTGGTCCGCAAGCCAAGGCAAAGAGCCGCGAGCAC    1440
FJ789783.1      CTTGCCACGATCGACGGTCGCATTGCTGGTCCGCAAGCCAAGGCAAAGAGCCGCGAGCAC    1440
NC_008040.1     CTTGCCACAATCGACGGTCGTATTGCTGGTCCGCAAGCCAAGGCAAAGAGCCGCGAGCAC    1440
EF558368.1      CTTGCCACAATCGACGGTCGTATTGCTGGTCCGCAAGCCAAGGCAAAGAGCCGCGAGCAC    1440
AY721616.1      CTTGCCACAATCGACGGTCGTATTGCTGGTCCGCAAGCCAAGGCAAAGAGCCGCGAGCAT    1440
KY354681.1      CTTGCCACAATCGACGGTCGTATTGCTGGTCCGCAAGCCAAGGCAAAGAGCCGCGAGCAC    1437
```

```
KP455643.1       CTTGCCACAATCGACGGTCGTATTGCTGGTCCGCAAGCCAAGGCAAAGAGCCGCGAGCAC      1440
MN309751.1       CTTGCCACAATCGACGGTCGTATTGCTGGTCCGCAAGCCAAGGCAAAGAGCCGCGAGCAC      1440
                 ******** ** ******** ***************** ********************

AY369136.1       ATAACACCTAAGATGCGCAAAGTGGCTAGGGACTTCGTGCACCATTTGGTGCCGGTTGCC      1500
KY315688.1       ATAACACCTAAGATGCGCAAAGTGGCTAGGGACTTCGTGCACCATTTGGTGCCGGTTGCC      1491
JN189909.2       ATAACACCTAAGATGCGCAAAGTGGCTAGGGACTTCGTGCACCATTTGGTGCCGATTGCC      1500
MT157513.1       ATAACACCTAAGATGCGCAAAGTGGCTAGGGATTTCGTGCACCATCTGGTGCCGATTGCC      1499
JN189865.2       ATAACACCTAAAATGCGCAAAGTGGCTAGGGATTTCGTGCACCATTTGGTGCCGGTTGCC      1500
MF144242.1       ATAACACCTAAGATGCGCAAAGTGGCTAGGGACTTCGTGCACCATCTGGTGCCGATTGCC      1500
AF319555.1       ATAACACCTAAGATGCGCAAAGTGGCTAGGGACTTCGTGCACCATCTGGTGCCGATTGCC      1500
FJ789783.1       ATAACACCTAAGATGCGCAAAGTGGCTAGGGACTTCGTGCACCATCTGGTGCCGATTGCC      1500
NC_008040.1      ATAACACCTAAGATGCGCAAAGTGGCTAGGGACTTCGTGCACCATCTGGTGCCGATTGCC      1500
EF558368.1       ATAACACCTAAGATGCGCAAAGTGGCTAGGGACTTCGTGCACCATCTGGTGCCGATTGCC      1500
AY721616.1       ATAACACCTAAGATGCGCAAAGTGGCTAGGGACTTCGTGCACCATCTGGTGCCGATTGCC      1500
KY354681.1       ATAACACCTAAGATGCGCAAAGTGGCTAGGGACTTCGTGCACCATCTGGTGCCGATTGCC      1497
KP455643.1       ATAACACCTAAGATGCGCAAAGTGGCTAGGGACTTCGTGCACCATCTGGTGCCGATTGCC      1500
MN309751.1       ATAACACCTAAGATGCGCAAAGTGGCTAGGGACTTCGTGCACCATCTGGTGCCGACTGCC      1500
                 *********** ******************** ************ ********  ****

AY369136.1       GGTACTGGCCGTCCCTACCCCCTCACGTATGTCGAGGAGCAGCAGACCAAGCCGTTACAG      1560
KY315688.1       GGTACTGGCCGTCCCTACCCCCTCACGTATGTCGAGGAGCAGCAGACCAAGCCGTTACAG      1551
JN189909.2       GGTACTGGCCGTCCCTACCCCCTCACGTATGTCGAGGAGCAGCAGACCAAGCCGTTACAG      1560
MT157513.1       GGTACTGGCCGTCCCTACCCCCTCACGTATGTCGAGGAGCAACAGACCAAGCCGTTACAG      1559
JN189865.2       GGCACTGGCCGTCCCTACCCCCTCACGTATGTCGAGGAGCAGCAGACCAAGCCGTTACAG      1560
MF144242.1       GGTACGGGCCGTCCCTACCCCCTCACGTATGTCGAGGAGCAGCAGACCAAGCCGTTACAG      1560
AF319555.1       GGCACTGGCCGTCCCTACCCCCTCACGTATGTCGAGGAGCAGCAGACCAAGCCGTTACAG      1560
FJ789783.1       GGCACTGGCCGTCCCTACCCCCTCACGTATGTCGAGGAGCAGCAGACCAAGCCGTTACAG      1560
NC_008040.1      GGTACTGGCCGTCCCTACCCCCTCACGTATGTCGAGGAGCAGCAGACCAAGCCGTTACAG      1560
EF558368.1       GGCACTGGCCGTCCCTACCCCCTCACGTATGTCGAGGAGCAGCAGACCAAGCCGTTACAG      1560
AY721616.1       GGCACTGGCCGTCCCTATCCCCTCACGTATGTCGAGGAGCAGCAGACCAAGCCGTTACAG      1560
KY354681.1       GGTACTGGCCGTCCCTACCCCCTCACGTATGTCGAGGAGCAGCAGACCAAGCCGTTACAG      1557
KP455643.1       GGTACGGGCCGTCCCTACCCCCTCACGTATGTCGAGGAGCAGCAGACCAAGCCGTTACAG      1560
MN309751.1       GGTACGGGCCGTCCCTACCCCCTCACGTATGTCGAGGAGCAGCAGACCAAGCCGTTACAG      1560
                 ** ** *********** *********************** ******************
Conventional PCR/qPCR forward primer:CCGATATCACGATGAGTTCAC
AY369136.1       CGGGCTCGGAATGATGCCAACCGATATCACGATGAGTTCACTATGATGGTTAAAGCGTTC      1620
KY315688.1       CGGGCTCGGAATGATGCTAACCGATATCACGATGAGTTCACTATGATGGTCAAAGCGTTC      1611
JN189909.2       CGGGCTCGGAATGATGCTAACCGATATCACGATGAGTTCACTATGATGGTCAAAGCGTTC      1620
MT157513.1       CGGGCTCGGAATGATGCTAACCGATATCACGATGAGTTCACTATGATGGTCAAAGCGTTC      1619
JN189865.2       CGGGCTCGGAATGATGCTAACCGATACCACGATGAGTTCACTATGATGGTCAAAGCGTTC      1620
MF144242.1       CGGGCTCGGAATGATGCTAACCGATATCACGATGAGTTTACTATGAAGGGCAAAGCGTTC      1620
AF319555.1       CGGGCTCGGAATGATGCTAACCGATATCACGATGAGTTCACTATGATGGTCAAAGCGTTC      1620
FJ789783.1       CGGGCTCGGAATGATGCTAACCGATATCACGATGAGTTCACTATGATGGTCAAAGCGTTC      1620
NC_008040.1      CGGGCTCGGAATGATGCTAACCGATATCACGATGAGTTCACTATGATGGTCAAAGCGTTC      1620
EF558368.1       CGGGCTCGGAATGATGCTAACCGATATCACGATGAGTTCACTATGATGGTCAAAGCGTTC      1620
AY721616.1       CGGGCTCGGAATGATGCTAACCGATATCACGATGAGTTCACTATGATGGTCAAAGCGTTC      1620
KY354681.1       CGGGCTCGGAATGATGCTAACCGATATCACGATGAGTTCACTATGATGGTCAAAGCGTTC      1617
KP455643.1       CGGGCTCGGAATGATGCTAATCGATATCACGATGAGTTCACAATGATGGTCAAGGCGTTC      1620
MN309751.1       CGGGCTCGGAATGATGCTAACCGATATCACGATGAGTTCACAATGATGGTCAAAGCCTTC      1620
                 ***************** ** ***** *********** ** **** **  ** ** ***

AY369136.1       CAAAAGAAAGAAGCATACAACGCCCCAAATTACCCCAGGAACATCTCAACCGTTCCGCAC      1680
KY315688.1       CAAAAGAAAGAAGCATACAACGCCCCAAATTACCCCAGGAACATCTCAACCGTTCCACAC      1671
JN189909.2       CAAAAGAAAGAAGCATACAACGCCCCAAATTATCCCAGGAACATTTCAACCGTTCCGCAC      1680
MT157513.1       CAAAAGAAAGAAGCATACAACGCCCCAAATTACCCCAGGAACATTTCAACCGTTCCGCAT      1679
JN189865.2       CAAAAGAAAGAAGCATACAACGCCCCAAATTACCCCAGGAACATTTCAACCGTTCCGCAC      1680
MF144242.1       CAAAAGAAAGAAGCATACAACGCCCCAAACTACCCCAGGAACATTTCAACCGTTCCGCAC      1680
AF319555.1       CAAAAGAAAGAAGCATACAACGCCCCAAATTATCCCAGGAACATTTCAACCGTTCCGCAT      1680
FJ789783.1       CAAAAGAAAGAAGCATACAACGCCCCAAATTACCCCAGGAACATTTCAACCGTTCCGCAT      1680
NC_008040.1      CAAAAGAAAGAAGCATACAACGCCCCAAATTACCCCAGGAACATTTCAACCGTTCCGCAT      1680
EF558368.1       CAAAAGAAAGAAGCATACAACGCCCCAAATTACCCCAGGAACATTTCAACCGTTCCGCAT      1680
AY721616.1       CAAAAGAAAGAAGCATACAACGCCCCAAATTACCCCAGGAACATTTCAACTGTTCCGCAT      1680
KY354681.1       CAAAAGAAAGAAGCATACAACGCCCCAAATTACCCCAGGAACATTTCAACCGTTCCGCAC      1677
KP455643.1       CAAAAGAAAGAAGCATACAACGCCCCAAATTACCCCAGGAACATTTCAACCGTTCCGCAT      1680
MN309751.1       CAAAAGAAAGAAGCATACAACGCCCCAAATTACCCCAGGAACATCTCAACCGTTCCGCAT      1680
                 ***************************** ** *********** ***** ***** **
                qPCR reverse primer:CCAGCTACACCTACGCTTTC
AY369136.1       ACCCAAAACGTCAAGTTGTCCAGCTACACCTACGCTTTCAAAGCCAGTGTTCTCCAGCAT      1740
KY315688.1       ACCCAAAACGTCAAGTTGTCCAGCTACACCTACGCTTTCAAAGCCAGTGTTCTCCAGCAT      1731
JN189909.2       ACCCAGAACGTCAAGTTGTCCAGCTACACCTACGCTTTCAAAGCCAGTGTTCTCCAGCAT      1740
MT157513.1       ACCCAGAACGTCAAGTTGTCCAGCTACACCTACGCTTTCAAGGCCAGTGTTCTCCAGCAT      1739
JN189865.2       ACCCAAAACGTCAAGTTGTCCAGCTACACCTACGCTTTCAAAGCCAGTGTTCTCCAGCAT      1740
MF144242.1       ACCCAAAACGTCAAGTTGTCCAGCTACACCTACGCTTTCAAAGCCAGTGTTCTCCAGCAT      1740
AF319555.1       ACCCAAAACGTCAAGTTATCCAGCTACACCTACGCTTTCAAAGCCAGTGTTCTCCAGCAT      1740
FJ789783.1       ACCCAAAACGTGAAGTTGTCCAGCTACACCTACGCTTTCAAAGCTAGTGTTCTCCAGCAT      1740
NC_008040.1      ACCCAAAACGTCAAGTTGTCCAGCTACACCTACGCTTTCAAAGCCAGTGTTCTCCAGCAT      1740
```

```
EF558368.1        ACCCAAAACGTTAAGTTGTCCAGCTACACCTATGCTTTCAAAGCCAGTGTTCTCCAGCAT    1740
AY721616.1        ACCCAAAACGTCAAGTTGTCCAGCTACACCTACGCTTTCAAAGCCAGTGTTCTCCAGCAT    1740
KY354681.1        ACCCAAAACGTCAAGTTGTCCAGCTACACCTACGCTTTCAAAGCCAGTGTTCTCCAGCAT    1737
KP455643.1        ACCCAGAACGTCAAGTTGTCCAGCTACACCTACGCTTTCAAAGCCAGTGTTCTCCAGCAT    1740
MN309751.1        ACCCAAAACGTCAAGTTGTCCAGCTACACCTACGCTTTCAAAGCCAGTGTTCTCCAGCAT    1740
                  ***** ***** ***** ************** ******** ** ***************

AY369136.1        GTTCCGTGGTACATGCCAACGCACACACCAGCTGAAATTGCTGACGCAGTGCAAAACTTG    1800
KY315688.1        GTTCCGTGGTACATGCCAACGCACACACCAGCTGAAATTGCTGACGCAGTGCAAAACTTG    1791
JN189909.2        GTTCCGTGGTACATGCCAACGCACACACCAGCTGAAATCGCTGACGCAGTGCAAAACTTG    1800
MT157513.1        GTTCCGTGGTACATGCCAACGCACACACCAGCTGAAATTGCTGACGCAGTGCAAAACTTG    1799
JN189865.2        GTTCCGTGGTACATGCCAACGCACACACCAGCGGAAATCGCTGACGCAGTGCAAAACTTG    1800
MF144242.1        GTTCCGTGGTACATGCCAACGCACACACCAGCGGAAATCGCTGACGCAGTGCAAAACTTG    1800
AF319555.1        GTTCCGTGGTACATGCCAACGCACACACCAGCTGAAATCGCTGACGCAGTGCAAAACTTG    1800
FJ789783.1        GTTCCGTGGTACATGCCAACGCACACACCAGCTCAAATCGCTGACGCAGTGCAAAACTTG    1800
NC_008040.1       GTTCCGTGGTACATGCCAACGCACACACCAGCTGAAATCGCTGACGCAGTGCAAAACTTG    1800
EF558368.1        GTTCCGTGGTACATGCCAACGCACACACCAGCTGAAATCGCTGACGCAGTGCAAAACTTG    1800
AY721616.1        GTTCCGTGGTACATGCCAACGCACACACCAGCTGAAATCGCTGACGCAGTGCAAAACTTG    1800
KY354681.1        GTTCCGTGGTACATGCCAACGCACACACCAGCTGAAATCGCTGATGCAGTGCAAAACTTG    1797
KP455643.1        GTTCCGTGGTACATGCCAACGCACACACCAGCTGAAATTGCTGACGCAGTGCAAAATTTG    1800
MN309751.1        GTTCCGTGGTACATGCCAACGCACACACCAGCTGAAATTGCTGACGCAGTGCAAAACTTG    1800
                  ********************************  **** ***** *********** ***

AY369136.1        GCCGCAAGTTCCACTGAGCTGGTTGAAACTGACTACAGCAAGTTCGATGGCACATTCTTG    1860
KY315688.1        GCCGCAAGTTCCACTGAGCTGGTTGAAACTGACTACAGCAAGTTCGATGGCACATTCTTG    1851
JN189909.2        GCCGCAAGTTCCACTGAGCTGGTCGAAACTGACTACAGCAAGTTCGATGGCACATTTTTG    1860
MT157513.1        GCTGCAAGTTCCACTGAGCTGGTTGAAACTGACTACAGCAAGTTCGATGGCACATTCTTG    1859
JN189865.2        GCCGCAAGTTCCACTGAGCTGGTTGAAACTGACTACAGCAAGTTCGATGGCACATTCTTG    1860
MF144242.1        GCCGCAAGTTCCACTGAGCTGGTCGAAACTGACTACAGCAAGTTCGATGGCACATTCTTG    1860
AF319555.1        GCTGCAAGTTCCACTGAGCTGGTTGAAACCGACTACAGCAAGTTCGATGGCACATTCTTG    1860
FJ789783.1        GCCGCAAGTTCCACTGAGCTGGTTGAAACTGACTACAGCAAGTTCGATGGCACATTCTTG    1860
NC_008040.1       GCCGCAAGTTCCACTGAGCTGGTTGAAACTGACTACAGCAAGTTCGATGGCACATTCTTG    1860
EF558368.1        GCCGCAAGTTCCACTGAGCTGGTTGAGACTGACTACAGCAAGTTCGATGGCACATTCTTG    1860
AY721616.1        GCCGCAAGTTCCACTGAGCTGGTTGAAACTGACTACAGCAAGTTCGATGGCACATTCTTG    1860
KY354681.1        GCCGCAAGTTCCACTGAGCTGGTTGAAACTGACTACAGCAAGTTCGATGGCACATTCTTG    1857
KP455643.1        GCCGCAAGTTCCACTGAGCTGGTTGAAACTGACTACAGCAAGTTCGATGGCACATTCTTG    1860
MN309751.1        GCCGCAAGTTCCACTGAGCTGGTTGAAACTGACTACAGCAAGTTCGATGGCACATTCTTG    1860
                  ** ******************** ** ** ************************** ***

AY369136.1        CGCTTTATGCGTGAGTGCGTCGAGTTTGCTATTTATAAGCGCTGGGTTCACCTGGACCAC    1920
KY315688.1        CGCTTCATGCGTGAGCGCGTCGAATTTGCTATTTATAAGCGCTGGGTTCACCTGGACCAC    1911
JN189909.2        CGCTTTATGCGTGAGTGCGTCGAATTTGCCATCTATAAGCGCTGGGTTCACCTGGACCAC    1920
MT157513.1        CGCTTCATGCGTGAGTGCGTCGAGTTTGCGATCTATAAGCGCTGGGTTCACCTGGACCAC    1919
JN189865.2        CGCTTTATGCGTGAGTGCGTCGAATTTGCCATCTATAAGCGCTGGGTTCACCTGGACCAC    1920
MF144242.1        CGCTTCATGCGTGAGTGCGTCGAATTTGCCATTTATAAGCGCTGGGTCCACCTGGACCAC    1920
AF319555.1        CGCTTTATGCGTGAGTGCGTCGAATTTGCTATCTATAAGCGCTGGGTTCACCTGGACCAC    1920
FJ789783.1        CGCTTTATGCGTGAGTGCGTTGAATTTGCTATCTATAAGCGCTGGGTTCACCTGGACCAC    1920
NC_008040.1       CGCTTCATGCGTGAGTGCGTCGAATTTGCTATCTATAAGCGCTGGGTTCACCTGGACCAC    1920
EF558368.1        CGCTTCATGCGTGAGTGCGTCGAATTTGCTATCTATAAGCGCTGGGTTCACCTGGACCAC    1920
AY721616.1        CGCTTCATGCGTGAGTGCGTCGAATTTGCTATCTATAAGCGCTGGGTTCACCTGGACCAC    1920
KY354681.1        CGCTTTATGCGTGAGTGCGTTGAATTTGCTATCTATAAGCGCTGGGTTCACCTGGACCAC    1917
KP455643.1        CGCTTTATGCGTGAGTGCGTCGAATTTGCCATCTATAAGCGCTGGGTTCACCTGGACCAC    1920
MN309751.1        CGCTTTATGCGTGAGTGCGTCGAATTTGCTATTTATAAGCGCTGGGTTCACCTGGACCAC    1920
                  ***** ********* **** ** ***** ** ************** ************

AY369136.1        TTGCCAGAATTAACAACTTTATTGGCTAATGAGATCCAAGCACCTGCTGTTACCCGATTG    1980
KY315688.1        TTGCCGGAGTTAACAACTTTATTGGCTAATGAGATCCAAGCACCTGCTGTTACCCGACTG    1971
JN189909.2        TTGCCAGAGTTAACAACTTTATTGGCTAATGAGATCCAAGCACCTGCTGTTACACGATTG    1980
MT157513.1        TTGCCAGAATTAACAACTTTATTGGCTAATGAGATTCAAGCACCTGCTGTTACACGATTG    1979
JN189865.2        TTGCCAGAGTTAACAACCTTATTGGCTAATGAGATCCAAGCACCTGCTGTTACACGACTG    1980
MF144242.1        TTGCCGGAGTTAACAACCTTATTGGCTAATGAGATCCAAGCACCTGCTGTTACACGACTG    1980
AF319555.1        TTGCCAGAGTTAACAACTTTATTGGCTAATGAGATCCAAGCACCTGCTGTTACACGACTG    1980
FJ789783.1        TTGCCAGAGTTAACAACTTTATTGGCTAATGAGATCCAAGCACCTGCTGTTACACGATTG    1980
NC_008040.1       TTGCCAGAGTTAACAACTTTATTGGCTAATGAGATCCAAGCACCTGCTGTTACACGATTG    1980
EF558368.1        TTGCCAGAACTAACAACTTTATTGGCTAATGAGATCCAAGCACCTGCTGTTACACGATTG    1980
AY721616.1        TTGCCAGAGTTAACAACTTTATTGGCTAATGAGATCCAAGCACCTGCTGTTACACGATTG    1980
KY354681.1        TTGCCAGAGTTAACAACTTTATTGGCTAATGAGATCCAAGCACCTGCTGTTACACGATTG    1977
KP455643.1        TTGCCAGAGTTAACAACTTTATTGGCTAATGAGATCCAAGCACCTGCTGTTACACGATTG    1980
MN309751.1        TTGCCAGAGTTAACAACTTTATTGGCTAATGAGATCCAAGCACCTGCTGTTACACGATTG    1980
                  ***** **  ******* ***************** ***************** *** **

AY369136.1        GGCATTAAATATGACCCTGATTGCAGTCGCCTCAGCGGTTCTGCTCTCACGACAGACGGA    2040
KY315688.1        GGCATCAAATACGACCCTGATTGCAGCCGCCTCAGCGGTTCTGCTCTCACAACAGACGGA    2031
JN189909.2        GGCATCAAGTATGACCCTGACTGCAGTCGCCTTAGTGGTTCTGCTCTCACTACAGACGGA    2040
MT157513.1        GGCATCAAGTATGATCCTGATTGCAGCCGCCTCAGTGGCTCTGCTCTCACGACCGACGGA    2039
JN189865.2        GGCATCAAGTATGATCCTGATTGCAGTCGCCTCAGTGGTTCTGCTCTCACGACAGACGGA    2040
MF144242.1        GGCATCAAGTATGATCCTGATTGCAGTCGCCTCAGTGGTTCTGCTCTCACGACAGACGGA    2040
```

```
AF319555.1      GGCATCAAGTATGACCCTGATTGCAGTCGCCTCAGTGGTTCTGCTCTCACAACCGACGGA    2040
FJ789783.1      GGCATCAAGTATGACCCTGATTGCAGCCGCCTCAGCGGTTCTGCTCTCACGACGGACGGA    2040
NC_008040.1     GGCATCAAGTATGACCCTGATTGCAGTCGCCTCAGTGGTTCTGCTCTCACGACAGACGGA    2040
EF558368.1      GGCATCAAGTATGAACCTGATTGCAGTCGCCTCAGTGGTTCTGCTCTCACGACAGACGGA    2040
AY721616.1      GGCATTAAGTATGACCCTGATTGCAGTCGCCTCAGTGGTTCTGCTCTCACAACAGACGGA    2040
KY354681.1      GGCATCAAGTATGACCCTGATTGCAGTCGCCTCAGTGGTTCTGCACTCACGACAGACGGA    2037
KP455643.1      GGCATCAAGTATGACCCTGATTGCAGTCGCCTCAGCGGCTCTGCTCTCACAACAGACGGA    2040
MN309751.1      GGCATCAAGTATGACCCTGATTGCAGTCGCCTCAGTGGCTCTGCTCTCACAACAGACGGA    2040
                ***** ** ** ** ***** ***** ***** ** ** ***** ***** ** ******

AY369136.1      AACAGCATTGCCAATGCTTTCGTCTCATACCTTGCTGGTCGTATGGCTGGCATGGATGAT    2100
KY315688.1      AACAGCATTGCCAATGCATTCGTCTCGTACCTTGCTGGTCGCATGGCTGGCATGGATGAT    2091
JN189909.2      AACAGCATTGCCAATGCTTTCGTCTCATACCTTGCTGGTCGTATGGCTGGCATGGATGAT    2100
MT157513.1      AACAGCATTGCCAATGCATTCGTCTCGTACCTTGCTGGTCGCATGGCTGGTATGGATGAC    2099
JN189865.2      AACAGCATTGCTAATGCGTTCGTCTCATACCTTGCTGGTCGCATGGCTGGCATGGATGAT    2100
MF144242.1      AACAGCATTGCTAATGCTTTCGTCTCATACCTTGCCGGTCGCATGGCTGGCATGGATGAT    2100
AF319555.1      AACAGCATTGCTAATGCTTTCGTCTCATACCTTGCTGGTCGCATGGCTGGCATGGATGAT    2100
FJ789783.1      AACAGCATTGCCAATGCTTTCGTCTCATACCTTGCTGGTCGCATGGCTGGCATGGATGAT    2100
NC_008040.1     AACAGCATTGCCAACGCTTTCGTCTCATACCTTGCTGGTCGCATGGCTGGCATGGATGAT    2100
EF558368.1      AACAGCATTGCCAATGCTTTCGTCTCATACCTTGCTGGTCGCATGGCTGGCATGGATGAT    2100
AY721616.1      AACAGCATTGCCAATGCCTTCGTCTCATACCTTGCTGGTCGCATGGCTGGCATGGATGAC    2100
KY354681.1      AACAGCATTGCCAATGCTTTCGTCTCATACCTTGCTGGTCGCATGGCTGGCATGGATGAC    2097
KP455643.1      AACAGCATTGCCAATGCTTTCGTCTCATACCTTGCTGGTCGTATGGCTGGCATGGATGAC    2100
MN309751.1      AACAGCATTGCCAATGCTTTCGTCTCATACCTTGCTGGTCGTATGGCTGGCATGGATGAC    2100
                *********** ** ** ******** ******** ***** ******** ******** 

AY369136.1      GACGAAGCTTGGTCTTGGATCGGCATTGTATACGGTGATGACGGGCTCAGATCTGGTAAT    2160
KY315688.1      GACGAAGCTTGGTCTTGGATCGGCATTGTATACGGTGATGATGGGCTCAGATCTGGTAAC    2151
JN189909.2      GATGAAGCTTGGTCTTGGATCGGCATTGTGTACGGTGATGATGGGCTCCGATCTGGTAAT    2160
MT157513.1      GATGAAGCTTGGTCTTGGATCGGCATTGTGTACGGTGATGACGGGCTCCGATCTGGTAAT    2159
JN189865.2      GATGAAGCTTGGTCTTGGATCGGCATTGTCTACGGTGATGATGGGCTCCGATCTGGTAAT    2160
MF144242.1      GATGAAGCTTGGTCTTGGATCGGCATTGTCTACGGTGATGATGGGCTCCGATCTGGTAAT    2160
AF319555.1      GATGAAGCTTGGTCTTGGATCGGCATTGTGTACGGTGATGATGGGCTCCGATCTGGTAAT    2160
FJ789783.1      GATGAAGCTTGGTCTTGGATCGGCATTGTTTACGGTGATGACGGGCTCCGATCTGGTAAT    2160
NC_008040.1     GATGAAGCTTGGTCTTGGATCGGCATTGTTTACGGTGATGATGGGCTCCGATCTGGTAAT    2160
EF558368.1      GATGAAGCTTGGTCTTGGATCGGCATTGTTTACGGTGATGATGGGCTCCGATCTGGTAAT    2160
AY721616.1      GATGAAGCTTGGTCTTGGATCGGCATTGTTTACGGTGATGACGGGCTCCGATCTGGTAAT    2160
KY354681.1      GATGAAGCTTGGTCTTGGATCGGCATTGTTTACGGTGATGATGGGCTCCGATCTGGTAAT    2157
KP455643.1      GATGAGGCTTGGTCTTGGATCGGCATTGTTTACGGTGATGATGGGCTCCGATCTGGTAAT    2160
MN309751.1      GATGAGGCTTGGTCTTGGATCGGCATTGTTTACGGTGATGACGGGCTCCGATCTGGTAAT    2160
                ** ** ********************** *********** ****** *********** 

AY369136.1      GTTTCAAATGAGCTCCTCACCAACACTGCTTCTTCCCTTGGCTTTGACTTGAAGATAGTG    2220
KY315688.1      GTTTCAAATGAGCTCCTCACCAACACTGCTTCTTCCCTTGGCTTTGACTTGAAGATAGTG    2211
JN189909.2      GTTTCAAATGAGCTCCTCACCAACACTGCTTCTTCCCTCGGCTTTGACTTGAAGATCGTG    2220
MT157513.1      GTTTCAAATGAGCTCCTCACCAACACTGCTTCTTCCCTTGGCTTTGACTTGAAGATTGTG    2219
JN189865.2      GTTTCAAATGAGCTCCTCACCAACACTGCTTCTTCCCTCGGCTTTGACTTGAAAATAGTG    2220
MF144242.1      GTTTCAAATGAGCTCCTCACCAACACTGCTTCTTCCCTCGGCTTTGACTTGAAAATAGTG    2220
AF319555.1      GTTTCAAACGAGCTCCTCACCAACACTGCTTCTTCCCTCGGCTTTGACTTGAAGATAGTG    2220
FJ789783.1      GTTTCAAACGAGCTCCTCACCAACACTGCTTCTTCCCTCGGCTTTGACTTGAAGATAGTG    2220
NC_008040.1     GTTTCAAATGAGCTCCTCACCAACACTGCTTCTTCCCTCGGCTTTGACTTGAAGATAGTG    2220
EF558368.1      GTTTCAAATGAGCTCCTCACCAACACTGCTTCTTCCCTCGGCTTTGACTTGAAGATAGTG    2220
AY721616.1      GTTTCAAATGAGCTCCTCACCAACACTGCTTCTTCCCTTGGCTTTGACTTGAAGATAGTG    2220
KY354681.1      GTTTCAAATGAGCTCCTCACCAACACTGCTTCTTCCCTCGGCTTTGACTTGAAGATAGTG    2217
KP455643.1      GTTTCAAATGAGCTCCTCACCAACACTGCTTCTTCCCTCGGCTTTGACTTGAAGATAGTG    2220
MN309751.1      ATTTCAAATGAGCTCCTCACCAACACTGCTTCTTCCCTCGGCTTTGACTTGAAGATAGTG    2220
                 ******* **************************** ************** ** ***

AY369136.1      AACCGCGCGCCACGTGGCTCCCCAGTGACATTCCTGTCTCGAGTATACCTCGATCCTTGG    2280
KY315688.1      AACCGCGCGCCACGTGGCTCCCCAGTGACATTCCTGTCTCGAGTATACCTCGATCCTTGG    2271
JN189909.2      AACCGCGCGCCACGTGGCTCTCCAGTGACATTCCTGTCTCGAGTATACCTCGATCCTTGG    2280
MT157513.1      AATCGCGCACCACGCGGCTCTCCAGTGACATTTCTGTCCCGAGTATACCTCGATCCTTGG    2279
JN189865.2      AATCGCGCGCCACGTGGCTCTCCAGTGACATTTCTGTCTCGAGTATACCTCGATCCTTGG    2280
MF144242.1      AATCGCGCGCCACGTGGCTCTCCAGTGACATTCCTGTCTCGAGTATACCTCGATCCTTGG    2280
AF319555.1      AATCGCGCGCCACGCGGCTCTCCAGTGACATTTCTGTCTCGAGTATACCTCGATCCTTGG    2280
FJ789783.1      AATCGCGCGCCACGTGGCTCTCCAGTGACATTTCTGTCTCGAGTATACCTCGATCCTTGG    2280
NC_008040.1     AATCGCGCGCCACGTGGCTCTCCAGTGACATTTCTGTCTCGAGTATACCTCGATCCTTGG    2280
EF558368.1      AATCGCGCGCCACGTGGCTCTCCAGTGACATTTCTGTCTCGAGTATACCTCGATCCTTGG    2280
AY721616.1      AATCGCGCGCCACGTGGCTCTCCAGTGACATTTCTGTCTCGAGTATACCTCGATCCTTGG    2280
KY354681.1      AATCGCGCGCCACGTGGCTCTCCAGTGACATTTCTGTCTCGAGTATACCTCGATCCTTGG    2277
KP455643.1      AATCGCGCGCCACGTGGCTCTCCAGTGACATTTCTGTCTCGAGTATACCTCGATCCTTGG    2280
MN309751.1      AATCGCGCGCCGCGTGGCTCTCCCGTGACATTTCTGTCTCGAGTATACCTCGATCCTTGG    2280
                ** ***** ** ** ***** ** ******** ***** ********************

AY369136.1      TCCTCACCGGCTTCCGTGCAGTCGCCATTAAGAACATTGTTGAAATTGCATACCACCTGT    2340
KY315688.1      TCCTCACCGGCCTCCGTGCAGTCGCCATTAAGAACATTGTTGAAATTGCATACCACCTGT    2331
JN189909.2      TCCTCACCGGCTTCCGTGCAGTCGCCATTAAGAACATTGTTGAAATTGCACACCACTTGT    2340
```

```
MT157513.1      TCCTCACCGGCTTCCGTGCAGTCGCCATTAAGAACATTGTTGAAATTGCACACCACCTGT   2339
JN189865.2      TCCTCACCGGCTTCCGTGCAGTCGCCATTAAGAACATTATTGAAATTGCACACCACCTGT   2340
MF144242.1      TCCTCACCGGCTTCCGTGCAGTCGCCATTAAGAACATTATTGAAATTGCACACCACCTGT   2340
AF319555.1      TCCTCACCGGCTTCCGTGCAGTCGCCATTAAGAACATTGTTGAAATTGCACACCACCTGT   2340
FJ789783.1      TCCTCACCGGCTTCCGTGCAGTCGCCATTAAGAACATTGTTGAAATTACACACCACCTGT   2340
NC_008040.1     TCCTCACCGGCTTCCGTGCAGTCGCCATTAAGAACATTGTTGAAATTGCACACCACCTGT   2340
EF558368.1      TCCTCACCGGCCTCCGTGCAGTCGCCATTAAGAACATTGTTGAAATTACACACCACCTGT   2340
AY721616.1      TCCTCACCGGCTTCCGTGCAGTCGCCATTAAGAACATTGTTGAAATTGCACACCACCTGT   2340
KY354681.1      TCCTCACCGGCTTCCGTGCAGTCGCCATTAAGAACATTGTTGAAATTGCACACCACCTGT   2337
KP455643.1      TCCTCACCGGCTTCCGTGCAGTCGCCATTAAGAACATTGTTGAAATTGCACACCACCTGC   2340
MN309751.1      TCCTCACCGGCTTCCGTGCAGTCGCCATTAAGAACATTGTTGAAATTGCACACCACCTGT   2340
                *********** ************************** ******** ** ***** **

      Conventional PCR reverse primer:GACATTGGCTGGGCTAAG
AY369136.1      GACACCCAGTCAGAGATTGACGACATTGGCTGGGCTAAGACACAGGCGTATTTGGTCACT   2400
KY315688.1      GACACCCAGTCAGAGATTGACGACATTGGCTGGGCTAAGACACAGGCGTATTTGGTCACT   2391
JN189909.2      GACACCCAGTCAGAGATTGAAGACATTGGCTGGGCTAAGACACAGGCGTATTTGGTCACT   2400
MT157513.1      GATACCCAGTCAGAGATTGACGACATTGGCTGGGCCAAGACACAGGCATATTTGGTCACT   2399
JN189865.2      GATACCCAGTCAGAGATTGACGACATTGGCTGGGCTAAGACACAGGCGTATTTGGTCACT   2400
MF144242.1      GATACCCAGTCAGAGATTGACGACATTGGCTGGGCTAAGACACAGGCGTATTTGGTTACT   2400
AF319555.1      GATACCCAGTCAGAGATTGACGACATTGGCTGGGCTAAGACACAGGCATATTTGGTCACT   2400
FJ789783.1      GATACCCAGTCAGAGATTGACGACATTGGCTGGGCTAAGACACAGGCGTATTTGGTCACT   2400
NC_008040.1     GATACCCAGTCAGAGATTGACGACATTGGCTGGGCTAAGACACAGGCGTACTTGGTCACT   2400
EF558368.1      GATACCCAGTCAGAGATTGACGACATTGGCTGGGCTAAGACACAGGCGTACTTGGTCACT   2400
AY721616.1      GACACCCAGTCAGAGATTGACGACATTGGCTGGGCTAAGACGCAGGCGTATTTGGTCACT   2400
KY354681.1      GACACCCAGTCAGAGATTGACGACATTGGCTGGGCTAAGACGCAGGCGTATTTGGTCACT   2397
KP455643.1      GACACCCAGTCAGAGATTGACGACATTGGCTGGGCTAAGACGCAGGCGTATTTGGTTACT   2400
MN309751.1      GACACCCAGTCAGAGATTGACGACATTGGCTGGGCTAAGACGCAGGCGTATTTGGTCACT   2400
                ** ***************** ************** ***** ***** ** ***** ***

AY369136.1      GATAGCAAGACACCCTTTATTGGTCATTGGTGCCGGGCTTATCAGAGAAATTGCACTGCA   2460
KY315688.1      GATAGCAAGACACCTTTTATTGGTCATTGGTGCCGGGCTTATCAGAGAAATTGCACTGCA   2451
JN189909.2      GATGGCAAGACACCTTTTATTGGTCATTGGTGCCGGGCTTATCAGAGAAATTGCACTGCA   2460
MT157513.1      GATAGCAAGACACCTTTTATTGGTCACTGGTGCCGGGCTTATCAGAGAAATTGCACTGCA   2459
JN189865.2      GATAGCAAGACACCTTTTATTGGTCACTGGTGCCGGGCTTATCAGAGAAATTGCACTGCA   2460
MF144242.1      GATAGCAAGACACCTTTTTT-GGTCATTGGTGCCGGGCTTATCAGAGAAATTCC-CTGCA   2458
AF319555.1      GATAGCAAGACACCTTTTATTGGTCATTGGTGCCGGGCTTATCAGAGAAATTGCACTGCA   2460
FJ789783.1      GATAGCAAGACACCTTTTATTGGTCATTGGTGCCGAGCTTATCAGAGAAATTGCACTGCA   2460
NC_008040.1     GATAGCAAGACACCTTTTATTGGTCATTGGTGCCGGGCTTATCAGAGAAATTGCACTGCA   2460
EF558368.1      GATAGCAAGACACCTTTTATTGGTCATTGGTGCCGGGCTTATCAGAGGAATTGCACTGCA   2460
AY721616.1      GATAGCAAGACACCTTTTATTGGTCATTGGTGCCGGGCTTATCAGAGAAATTGCACTGCA   2460
KY354681.1      GATAGCAAGACACCTTTTATTGGTCATTGGTGCCGGGCTTATCAGAGAAATTGCACTGCA   2457
KP455643.1      GATAGCAAGACACCTTTTATTGGTCATTGGTGCCGGGCTTATCAGAGAAATTGCACTGCA   2460
MN309751.1      GATAGCAAGACACCTTTTATTGGTCATTGGTGCCGGGCCTATCAGAGAAATTGCACTGCA   2460
                *** ********** *** * ***** ******** ** ******** **** * *****

AY369136.1      CGTGTGGTCCAGTATGCAGACTACACTGACATTCCATTCTGGGTGAAGAATGACGACCAC   2520
KY315688.1      CGTGTGGTCCAGTATGCAGACTACACTGATATTCCATTCTGGGTGAAGAATGACGACCAC   2511
JN189909.2      CGTGTGGTCCAGTATGCAGACTACACTGACATTCCATTTTGGGTGAAGAACGACGACCAC   2520
MT157513.1      CGTGTGGTCCAGTATGCAGACTACGCTGACATTCCATTCTGGGTGAAGAACGACGACCAC   2519
JN189865.2      CGTGTGGTTCAGTATGCAGACTACACTGACATTCCATTCTGGGTGAAGAATGACGACCAC   2520
MF144242.1      CGTGTGGTTCAGT-ATGCGACTACACTGACATTCCATTCTGGGTGAAGAATGACGACCAC   2517
AF319555.1      CGTGTGGTCCAGTATGCAGACTACGCTGACATTCCATTCTGGGTGAAGAACGACGACCAC   2520
FJ789783.1      CGTGTGGTTCAGTATGCAGACTACACTGACATTCCATTCTGGGTGAAGAATGACGACCAC   2520
NC_008040.1     CGTGTAGTTCAGTATGCAGACTACACTGACATTCCATTCTGGGTGAAGAATGACGACCAC   2520
EF558368.1      CGTGTGGTTCAGTATGCAGACTACACTGACATTCCATTCTGGGTGAAGAATGACGACCAC   2520
AY721616.1      CGTGTGGTTCAGTATGCAGACTACACTGACATTCCATTCTGGGTGAAGAATGACGACCAC   2520
KY354681.1      CGTGTGGTTCAGTATGCAGACTACACTGACATTCCATTCTGGGTGAAGAATGACGACCAC   2517
KP455643.1      CGTGTGGTTCAGTATGCAGACTACAGTGACATTCCATTCTGGGTGAAGAATGACGACCAC   2520
MN309751.1      CGTGTGGTTCAGTATGCAGACTACAGTGACATTCCATTCTGGGTGAAGAATGACGACCAC   2520
                ***** ** ****     ******  *** ******** *********** *********

AY369136.1      GTTGGCAACTCGTGGCCGCAGTCTGATTCCGACGACTGGAATGACATTGTAGCCAATGAG   2580
KY315688.1      GTTGGCAACTCGTGGCCGCAGTCTGAATCCGATGACTGGAACGACATTGTAGCCAATGAG   2571
JN189909.2      GCTGGCAAGTCGTGGCCGCAGTCTGAGTCCGATGACTGGAATGACGTTGTAGCCAACGAG   2580
MT157513.1      GTTGGCAACTCGTGGCCGCAGTCTGAATCTGATGACTGGAATGACATTGTAGCCAACGAG   2579
JN189865.2      GTTGGCAACTCGTGGCCGCAGTCTGAATCCGATGACTGGAATGACGTTGTAGCCAACGAG   2580
MF144242.1      GTTGGCAACTCGTGGCCGCAGTCTGAATCCGATGACTGGAATGACGTTGTAGCCAACGAG   2577
AF319555.1      GTTGGCAACTCGTGGCCGCAGTCTGAATCCGATGACTGGAATGACATTGTAGCCAACGAG   2580
FJ789783.1      GTTGGCAACTCGTGGCCGCAGTCTGAATCCGATGACTGGAATGACGTTGTAGCCAACGAG   2580
NC_008040.1     GTCGGCAACTCGTGGCCGCAGTCTGAATCCGATGACTGGAATGACGTTGTAGCCAACGAG   2580
EF558368.1      GTTGGCAACTCGTGGCCGCAGTCTGAATCTGATGACTGGAATGACGTTGTAGCCAACGAG   2580
AY721616.1      GTTGGCAACTCGTGGCCGCAGTCTGAATCCGATGACTGGAATGACGTTGTAGCCAACGAG   2580
KY354681.1      GTTGGCAACTCGTGGCCGCAGTCTGAATCCGATGACTGGAATGACGTTGTAGCCAACGAG   2577
KP455643.1      GTTGGCAACTCGTGGCCGCAGTCTGAATCCGATGACTGGAATGACGTTGTAGCCAACGAG   2580
MN309751.1      GTTGGCAACTCGTGGCCGCAGTCTGAATCCGATGACTGGAATGACGTTGTAGCCAACGAG   2580
                *  ***** ***************** ** ** ******** *** ********** ***
```

```
AY369136.1        CTTGGCGTCACCACCGCCGAGCTGTTGAAGCATCTTGCACTTCTAGATGCTTATACTGGT    2640
KY315688.1        CTTGGCGTCACCACCGCCGAGCTGTTGAAGCATCTTGCACTTCTAGATGCTTATACTGGT    2631
JN189909.2        CTTGGCGTCACCACCGCCGAGCTGTTGAAGCATCTTGCACTTCTGGATGCTTATGCTGGT    2640
MT157513.1        CTTGGCGTCACCACCGCTGAGCTGTTGAAGCATCTTGCACTTCTGGATGCTTATGCTGGT    2639
JN189865.2        CTTGGCGTCACCACCGCTGAGCTGTTGAAGCATCTTGCACTCCTGGATGCTTATACTGGT    2640
MF144242.1        CTTGGCGTCACCACCGCTGAGCTGTTGAAGCATCTTGCACTCCTGGATGCTTATACTGGT    2637
AF319555.1        CTTGGCGTCACCACCGCTGAGCTGTTGAAGCATCTTGCACTTCTGGATGCTTATGCTGGT    2640
FJ789783.1        CTTGGCGTCACCACCGCTGAGCTGTTGAAGCATCTTGCACTCCTGGATGCTTATACTGGT    2640
NC_008040.1       CTCGGCGTCACCACCGCTGAGCTGTTGAAGCATCTTGCACTTCTGGATGCTTATACTGGT    2640
EF558368.1        CTTGGCGTCACCACCGCTGAGCTGTTGAAGCATCTCGCACTTCTGGATGCTTATACTGGT    2640
AY721616.1        CTTGGCGTCACCACCGCTGAGCTGTTGAAGCATCTTGCACTTCTGGATGCTTATACTGGT    2640
KY354681.1        CTTGGCGTCACCACCGCTGAGCTGTTGAAGCATCTTGCACTTCTGGATGCTTATACTGGT    2637
KP455643.1        CTTGGCGTCACCACCGCTGAGCTGTTGAAGCATCTTGCACTTCTGGATGCTTATACTGGT    2640
MN309751.1        CTTGGCGTCACCACCGCTGAGCTGTTGAAGCATCTTGCACTTCTGGATGCTTATACTGGT    2640
                  ** ************** **************** ***** ** ********* *****

AY369136.1        CCCATTAATGGCCTCCCACGTCTGACGACATCAATCGATTTGGAACCAAAGATGTCTGTC    2700
KY315688.1        CCCATTAATGGCCTCCCACGTCTGACAACATCAATCGATTTGGAACCAAAGATGTCTGTC    2691
JN189909.2        CCCATCAGTGGCCTCCCACGTTTGACAACATCAATCGATTTGGAACCAAAGATGTCTGTC    2700
MT157513.1        CCCATCAGTGGCCTCCCGCGTCTGACAACATCAATTGATTTGGAACCAAAGATGTCTGTC    2699
JN189865.2        CCCATTAGTGGCCTCCCACGTCTGACAACATCAATCGATTTGGAACCAAAGATGTCTGTC    2700
MF144242.1        CCCATTAGTGGCCTCCCACGTCTGACAACATCAATCGATTTGGAACCAAAGATGTCTGTC    2697
AF319555.1        CCCATCAGTGGCCTCCCACGTCTGACAACATCAATCGATTTGGAACCAAAGATGTCTGTC    2700
FJ789783.1        CCCATCAGTGGCCTCCCACGTCTGACAACATCAATCGATTTGGAACCAAAGATGTCTGTC    2700
NC_008040.1       CCCATCAGTGGCCTCCCACGTCTGACAACATCAATCGATTTGGAACCAAAGATGTCTGTC    2700
EF558368.1        CCCATCAGTGGCCTCCCACGTCTGACAACATCAATCGATTTGGAACCAAAGATGTCTGTC    2700
AY721616.1        CCCATTAGTGGCCTCCCACGTCTGACAACATCAATCGATTTGGAACCAAAGATGTCTGTC    2700
KY354681.1        CCCATTAGTGGCCTCCCACGTCTGACAACATCAATCGATTTGGAACCAAAGATGTCTGTC    2697
KP455643.1        CCCATTAGCGGCCTCCCACGTCTGACAACATCAATCGATTTGGAACCAAAGATGTCTGTC    2700
MN309751.1        CCCATTAGCGGCCTCCCGCGTCTGACAACATCAATTGATTTGGAACCAAAGATGTCTGTC    2700
                  ***** *  ******** *** **** ******** ***********************

AY369136.1        GCATTAGACGGGGAGATCCAAGCCGGTCCTAGTCAAAACAAAACTAGCAAGGATGGAACA    2760
KY315688.1        GCATTAGACGGGGAGATCCAAGCCGGTCCTAGTCAAAACAACACTAGCAAGGATGGAACA    2751
JN189909.2        GCATTAGACGGGGAGATCCAAGCCGGTCCTAGTCAAAACAAAACTAGCAAGGATGGAACA    2760
MT157513.1        GCATTAGACGGGGAGATCCAAGCCGGTCCTAGTCAAAACAAAACTAGCAAGGATGGAACA    2759
JN189865.2        GCATTAGACGGGGAGATCCAAGCCGGTCCTAGTCAAAACAAAACTAGCAAGGATGGAACA    2760
MF144242.1        GCATTAGACGGGGAGATCCAAGCCGGTCCTAGTCAAAACAAAACTAGCAAGGATGGAACA    2757
AF319555.1        GCATTAGATGGGGAGATCCAAGCCGGTCCTAGTCAAAACAAAACTAGCAAGGATGGAACA    2760
FJ789783.1        GCATTAGACGGGGAGATCCAAGCCGGTCCTAGTCAAAACAAAACTAGCAAGGATGGAACA    2760
NC_008040.1       GCATTGGACGGGGAGATCCAAGCCGGTCCTAGTCAAAACAAAACTAGCAAGGATGGAACA    2760
EF558368.1        GCATTAGACGGGGAGATCCAAGCCGGTCCTAGTCAAAACAAAACTAGTAAGGATGGAACA    2760
AY721616.1        GCATTAGACGGGGAGATCCAAGCCGGTCCTAGTCAAAACAAAACTAGTAAGGATGGAACA    2760
KY354681.1        GCATTGGACGGGGAGATCCAAGCCGGTCCTAGTCAAAACAAAACTAGTAAGGATGGAACA    2757
KP455643.1        GCATTAGACGGGGAGATCCAAGCCGGTCCTAGTCAAAACAAAACTAGTAAGGATGGAACG    2760
MN309751.1        GCATTAGACGGGGAGATCCAAGCCGGTCCTAGTCAAAACAAAACTAGTAAGGATGGAACA    2760
                  ***** ** ******************************** ***** ***********

AY369136.1        AATCCAACAAGCGATCGATCAGCACCTCGTCGAGCTCGAGCAGCTCTTCCAGGTGATGAT    2820
KY315688.1        AATCCAACAAGCGATCGATCAGCACCTCGTCGAGCTCGAGCAGCTCTTCCAGGTGATGAT    2811
JN189909.2        AATCCAACAAGCGATCGATCAGCACCTCGTCGAGCTCGAACAGCTCTTCCAGGTGATGAT    2820
MT157513.1        AATCCAACAAGCGATCGATCAGCACCTCGTCGAGCTCGAGCAGCTCTTCCAGGTGATGAT    2819
JN189865.2        AATCCAACAAGCGATCGATCAGCACCTCGTCGAGCTCGAGCAGCTCTTCCAGGTGATGAT    2820
MF144242.1        AATCCAACAAGCGATCGATCAGCACCTCGTCGAGCTCGAGCAGCTCTTCCAGGTGATGAT    2817
AF319555.1        AATCCAACAAGCGATCGATCAGCACCTCGTCGAGCTCGAGCAGCTCTTCCAGGTGATGAT    2820
FJ789783.1        AATCCAACAAGCGATCGATCAGCACCTCGTCGAGCTCGAGCAGCTCTTCCAGGTGATGAT    2820
NC_008040.1       AATCCAACAAGCGATCGATCAGCACCTCGTCGAGCTCGAGCAGCTCTTCCAGGTGATGAT    2820
EF558368.1        AATCCAACAAGCGATCGATCAGCACCTCGTCGAGCTCGAGCAGCTCTTCCAGGTGATGAT    2820
AY721616.1        AATCCAACAAGCGATCGATCAGCACCTCGTCGAGCTCGAGCAGCTCTTCCAGGTGATGAT    2820
KY354681.1        AATCCAACAAGCGATCGATCAGCACCTCGTCGAGCTCGAGCAGCTCTTCCAGGTGATGAT    2817
KP455643.1        AATCCAACAAGCGATCGATCAGCTCCTCGTCGAGCTCGAGCAGCTCTTCCAGGTGATGAT    2820
MN309751.1        AATCCAACAAGCGATCGATCAGCACCTCGTCGAGCTCGAGCAGCTCTTCCAGGTGATGAT    2820
                  *********************** *************** ********************

AY369136.1        GGACACGCGCGTCGCTCTCGGCGGAGTGACCGCGATCCAGGTAAACGAGATGCGCACGTT    2880
KY315688.1        GGACACGCGCGTCGCTCTCGGCGGAGTGACCGCGATCCAGGTAAACGAGATGCGCACGTT    2871
JN189909.2        GGACACGCGCGTCGCTCTCGGCGGAGTGACCGCGATCCAGGTAAACGAGATGCGCACGTT    2880
MT157513.1        GGACACGCGCGTCGCTCTCGGCGGAGTGACCGCGATCCAGGTAAACGAGATGCGCACGTT    2879
JN189865.2        GGACACGCGCGTCGCTCTCGGCGGAGTGACCGCGATCCAGGTAAACGAGATGCGCACGTT    2880
MF144242.1        GGACACGCGCGTCGCTCTCGGCGGAGTGACCGCGATCCAGGTAAACGAGATGCGCACGTT    2877
AF319555.1        GGACACGCGCGTCGCTCTCGGCGGAGTGACCGCGATCCAGGTAAACGAGATGCGCACGTT    2880
FJ789783.1        GGACACGCGCGTCGCTCTCGGCGGAGTGACCGCGATCCAGGTAAACGAGATGCGCACGTT    2880
NC_008040.1       GGACACGCGCGTTGCTCTCGGCGGAGTGACCGCGATCCAGGTAAACGAGATGCGCACGTT    2880
EF558368.1        GGACACGCGCGTCGCTCTCGGCGGAGTGACCGCGATCCAGGTAAACGAGATGCGCACGTT    2880
AY721616.1        GGACACGCGCGTCGCTCTCGGCGGAGTGACCGCGATCCAGGTAAACGAGATGCGCACGTT    2880
KY354681.1        GGACACGCGCGTCGCTCTCGGCGGAGTGACCGCGATCCAGGTAAACGAGATGCGCACGTT    2877
KP455643.1        GGACACGCGCGTCGCTCTCGGCGGAGTGACCGCGATCCAGGTAAACGAGATGCGCACGTT    2880
```

```
MN309751.1      GGACACGCGCGTCGCTCTCGGCGGAGTGACCGCGATCCAGGTAAACGAGATGCGTACGTT     2880
                ************ ***************************************** *****

AY369136.1      CGTGATAAGCGCTCACGCCGCAGCTCGCCGCCTACACGTCCTGTCACGCCGGTTTCCGCC     2940
KY315688.1      CGTGACAAGTGCTCACGCCGCAGCTCGCCGCCTACACGTCCTGTCTCGCCGGTTTCCGCC     2931
JN189909.2      CGTGATAAGCGCCCACGCCGCAGCCCGTCGCCTACACGTCCTGTCACGCCGGTTCCCGCC     2940
MT157513.1      CGTGATAAGCGCCCACGCCGCAGCTCGCCGCCTGCACGTCCTGTCACGCCGGTTCCCGCC     2939
JN189865.2      CGTGATAAGCGCCCACGCCGCAGCTCGCCGCCTGCACGTCCTGTCACGCCGGTTCCCACC     2940
MF144242.1      CGTGATAAGCGCCCACGCCGCAGCTCGCCGCCTGCACGTCCTGTCGCGCCGGTTCCCACC     2937
AF319555.1      CGTGATAAGCGCCCACGCCGCAGCTCGCCGCCTACACGTCCTGTCACGCCGGTTCCCACC     2940
FJ789783.1      CGTGATAAGCGCCCACGCCGCAGCTCGCCGCCTACACGTCCTGTCACGCCGGTTCCCACC     2940
NC_008040.1     CGTGGTAAGCGCTCACGCCGCAGCTCGCCGCCTGTACGTCCTGTCACGCCGGTTCCCACC     2940
EF558368.1      CGTGATAAGCGCCCACGCCGCGGCTCGCCGCCTACACGTCCTGTCACGCCGGTTCCCACC     2940
AY721616.1      CGTGATAAGCGCCCACGCCGCAGCTCGCCGCCTGCACGTCCTGTCACGCCGGTTCCCACC     2940
KY354681.1      CGTGATAAGCGCCCACGCCGCAGCTCGCCGCCTGCACGTCCTGTCACGCCGGTTCCCACC     2937
KP455643.1      CGTGATAAGCGCCCACGCCGCAGCTCGCCGCCTGCACGTCCTGTCACGCCGGTTCCCACC     2940
MN309751.1      CGTGATAAGCGCCCACGCCGCAGCTCGCCGCCTGCACGTCCTGTCACGCCGGTTCCCACC     2940
                ****  *** ** ******** ** ** *****  ********** ******** ** **

AY369136.1      CGCTCTAGCGGTGATCGAGGAACCGATGGAGACAGACTAGGCCGAGCTGTTGTGCGTCAG     3000
KY315688.1      CGCTTTAGCGGTGATCGAGGAACCGGTGGAGACAGACTAGGCCGAGCTGTTGTGCGTCAG     2991
JN189909.2      CGCTCTAGCGGTGATCGTGGAACCGATGGAGACGGACTAGGCCGAGCTGCTGTGCGTCAG     3000
MT157513.1      TCTTCCAGCAGTGGTCGAGGAACCGATGGAGACGGACTAGGCCGAGCTGCTGTGCGTCAG     2999
JN189865.2      CCTTCCAGCGGTGATCGAGGAACCGATGGAGACGGACTAGGCCGAGCTGCTGTGCGCCAG     3000
MF144242.1      CCTTCCAGTGGTGATCGAGGAACCGATGGAGACGGACTAGGCCGAGCTGCTGTGCGTCAG     2997
AF319555.1      CCTTCCAGCGGTGATCGAGGAACCGATGGAGACGGACTAGGCCGAGCTGCTGTGCGTCAG     3000
FJ789783.1      CCTTCCAGCGGTGATCGAGGAACCGTAGGAGACGGACTAGGCCGAGCTGCTGTGCGTCAG     3000
NC_008040.1     CCTTCCAGCGGTGATCGAGGAACCGATGGAGACGGACTAGGCCGAGCTGCTGTGCGTCAG     3000
EF558368.1      CCTTCCAGCGGTGATCGAGGAGCCGATGGAGACGGACTAGGCCGAGCTGCTGTGCGTCAG     3000
AY721616.1      CCTTCCAGCGGTGATCGAGGAACCGATGGAGACGGACTAGGCCGAGCTGCTGTGCGTCAG     3000
KY354681.1      CCTTCCAACGGTGATCGAGGAACCGATGGAGACGGACTAGGCCGAGCTGCTGTGCGTCAG     2997
KP455643.1      CCTTCCAGCGGTGATCGAGGAACCGATGGAGACGGACTAGGCCGAGCTGCTGTGCGTCGG     3000
MN309751.1      CCTTCCAGCGGTGATCGAGGAACCGATGGAGACGGACTAGGCCGAGCTGCTGTGCGTCAG     3000
                   *  *   *** *** *** ***  ****** *************** ****** * *

AY369136.1      CGTCAACGACGTCGCACTCAAGTGTAGACAGGTCACTTGCCTGCTCCCACCCC-CTGGAC     3059
KY315688.1      CGTCAACGACCTCGCACTCAAGTGTAGACAGGTCACTTGCCTGCTCCCACCCC-CTGGAC     3050
JN189909.2      CGTCAGCGACGTCGCACTCAGGTGTAGACAGGTCACTTGCCTGCTCCCACCCCCCCGGAC     3060
MT157513.1      CGTCAGCGACGTCGCACTCAAGAGTAGACAGGTCACTTGCCTGCTCCCACCCCCT-GGAC     3058
JN189865.2      CGTCAGCGACGTCGCACTCAAGTGTAGACAGGTCACTTGCCTGCTCCCACCCCCT-GGAC     3059
MF144242.1      CGTCAGCGACGTCGCACTCAAGTGTAGACAGGTCACCTGCCTGCTCCCACCCCCT-GGAC     3056
AF319555.1      CGTCAGCGACGTCGCACTCAAGTGTAGACAGGTCACTTGCCTGCTCCCACCCCTT-GGAC     3059
FJ789783.1      CGCCATCGACGTCGCACTCAAGTGTAGACGGGTCACCTGCCTGCTCCCACCCCCCG-GAC     3059
NC_008040.1     CCCCGGCGACGTCGCACTCAAGTGTAGACAGGTCACCTGCCTGCTCCCACCCCCCTGGAC     3060
EF558368.1      CGCCAGGGACGTCGCACTCAAGTGTAGACAGGTCACCTGCCTGCTCCCACCCC-CTGGAC     3059
AY721616.1      CGCCAGCGACGTCGCACTCAAGTGTAGACAGGTCACCTGCCTGCTCCCACCCC-CTGGAC     3059
KY354681.1      CGCCAGCGACGTCGCACTCAAGTGTAGACAGGTCACCTGCCTGCTCCCACCCC-C-----     3051
KP455643.1      CGTCAGCGACGTCGCACTCAAGTGTAGACAGGTCACCTGCCCGCTCCCACCCC-CTGGAC     3059
MN309751.1      CGTCAGCGACGTCGCACTCAAGTGTAGACAGGTCACCTGCCTGCTCCCACCCC-CTGGAC     3059
                *  *   *** ********* * ****** ****** **** ***********

AY369136.1      CGTTTTGGTCCCTTAATCAGCTTTATGCTGTCCTACGCTTCGGC-     3103
KY315688.1      CGTTTTGGTCCCTTAATCAGCTTTATGCTATCTTACGCTTCGGCA     3095
JN189909.2      CGTCTTGGTCCCTTAATCAGCTTTATGCTGTCTTACGCTTCGGC-     3104
MT157513.1      CGTTTTGGTCCCTTAATCAGCTTTATGCTGTCCTACGCTTCGGCG     3103
JN189865.2      CGTTTTGGTCCCTTAATCAGCTTTATGCTGTCTTACGCTTCGGC-     3103
MF144242.1      CGTTTCGGTCCCTTAATCAGCTTTATGCTGTCCTACGCTTCGGC-     3100
AF319555.1      CGTTTTGGTCCCTTAATCAGCTTTATGCTGTCCTACGCTTCGGC-     3103
FJ789783.1      CGGTTTGGTCCCTTAATCAGCTTTATGCTGTCCTACGCTTCGGCG     3104
NC_008040.1     CGTTTTGGTCCCTTAATCAGCTTTATGCTGTCCTACGCTTCGGCG     3105
EF558368.1      CGTTTTGGTCCCTTAATCAGCTTTATGCTGTCCTACGCTTCGGC-     3103
AY721616.1      CGTTTTGGTCCCTTAATCAGCTTTATGCTGTCCTACGCTTCGGC-     3103
KY354681.1      ---------------------------------------------     3051
KP455643.1      CATTTTGGTCCCTTAATCAGCTTTATGCTGTCCTACGCTTCGGC-     3103
MN309751.1      CGTTTCGGTCCCTTGATCAGCTTTATGCTGTCCTACGCTTCGGC-     3103
```

Figure S2. Multiple sequence alignment of RNA2 sequences from the selected genotype representatives.

```
GB acc. no                                                                       bp
           Conventional PCR forward primer:AATGGTACGCAAAGGTGA
KY354702.2      ----------CCGCTTTGCAAATCAAAATGGTACGCAAAGGTGATAAGAAATTGGCAAAA    50
KF386164.1      TAATTCGACATCGCTTTGCATCTCACAATGGTACGCAAGGGTGAGAAGAAATTGGCTAAA    60
JX402858.1      ------------------------------------------------------------    0
KY315689.1      -TAATTCAGCACCGCTTTGCAATCACAATGGTACGCAAAGGTGAGAAGAAATTGGCAAAA    59
JN189992.2      -TAATCCATCACCGCTTTGCAATCACAATGGTACGCAAAGGTGAGAAGAAATTGGCAAAA    59
GU592791.1      ----------------TNNTNCNACNAATGGGTACGCAAGGTGAGAAGAAATTGGCAAAA    44
AF499774.1      ---------------------------ATGGTACGCAAAGGTGATAAGAAATTGGCAAAA    33
MG637439.1      -TAATCCATCACCGCTTTGCAATCACAATGGTACGCAAAGGTGAGAAGAAATTGGCAAAG    59
KU705815.1      ------------------CCCCCTACAATGGTACGCAAAGGTGAGAAGAAATTGGCAAAA    43
AF245004.1      -------------CTTTGCAAGTCAAAATGGTACGCAAAGGTGAAAAGAAATTGGCAAAA    47
JF412263.1      -TAATCCATCACCGCTTTGCAATCACAATGGTACGCAAAGGTGAGAAGAAATTGGCAAAA    59
MN309752.1      -TAATCCATCACCGCTTTGCAATCACAATGGTACGCAAAGGTGAGAAGAAATTGGCAAAA    59
KM588181.1      ----TCCTTCATCGCTTTGCAATCACAATGGTACGCAAAGGTGAGAAGAAATTGGCAAAA    56
FJ789784.1      -TAATCCACCACCGCTTTGCAATCACAATGGTACGCAAAGGTGAGAAGAAATTGGCAAAA    59
AF245003.1      -------------CTTTGCAAGTCAAAATGGTACGCAAAGGTGAAAAGAAATTGGCAAAA    47
NC_008041.1     -TAATCCATCACCGCTTTGCAATCACAATGGTACGCAAAGGTGAGAAGAAATTGGCAAAA    59
AY744705.1      -TAATCCATCACCGCTTTGCAATCACAATGGTACGCAAAGGTGAGAAGAAATTGGCAAAA    59


KY354702.2      CCTGCGACCACAAAGGCCGCCAATCCTCAACCACGTCGACGTGCAACACAGCGCCGTCGC    110
KF386164.1      ACAGCGACCACCAAGGCCGCTAATCCTCAACCCCGTCGACGTGCCGCCAACCGGCGGCGT    120
JX402858.1      -CCGCGACCACCAAGGCCGCCAATCCGCAACCCCGTCGACGTGCTAACAATCGTCGGCGT    59
KY315689.1      CCCGCGACCACCAAGGCCGCGAATCCGCAACCCCGCCGACGTGCTAACAATCGTCGGCGT    119
JN189992.2      CCCGCGACCACCAAGGCCGCGAATCCGCAACCTCGTCGACGTGCTAACAATCGTCGGCGT    119
GU592791.1      CCCGCGACCACCAAGGCCGCGAATCCGCAACCCCGCCGACGTGCTAACAATCGTCGGCGT    104
AF499774.1      CCCGCGACCACCAAGGCCGCGAATCCGCATCCCCGCCGACGTGCTAACAATCGTCGGCGT    93
MG637439.1      CCCGCGACCACCAAGGCCGCGAATCCGCAACCCCGCCGACGTGCTAACAATCGTCGGCGT    119
KU705815.1      CCCGCGACCACCAAGGCCGCGAATCCGCAACCCCGCCGACGTGCTAACAATCGTCGGCGT    103
AF245004.1      CCCCCGACCACCAAGGCCGCGAATCCGCAACCCCGCCGACGTGCTAACAATCGTCGGCGT    107
JF412263.1      CCCGCGACCACCAAGGCCGCGAATCCGCAACCCCGCCGACGAGCTAACAATCGTCGGCGT    119
MN309752.1      CCCGCGACCACCAAGGCCGCGAATCCGCAACCCCGCCGACGTGCTAACAATCGTCGGCGT    119
KM588181.1      CCCGCGACCACCAAGGCCGCGAATCCGCAACCCCGCCGACGTGCTAACAATCGTCGGCGT    116
FJ789784.1      CCCGCGACCACCAAGGCCGCGAATCCGCAACCCCGTCGACGTGCTAACAATCGTCGGCGT    119
AF245003.1      CCCGCGACCACCAAGGCCGCGAATCCGCAACCCCGCCGACGTGCCAACAATCGTCGGCGT    107
NC_008041.1     CCCGCGACCACCAAGGCCGCGAATCCGCAACCCCGCCGACGTGCTAACAATCGTCGGCGT    119
AY744705.1      CCCGCGACCACTAAGGCCGCGAATCCGCAACCCCGCCGACGTGCTAACAATCGTCGGCGT    119
                 *  ******* ******** ***** ** ** ** ***** **     * ** ** **

KY354702.2      AGTGGTAGGGCTGATGCACCCTTAGCTAAGGCATCGACTATCACGGGATTTGGACGTGCG    170
KF386164.1      AGTAATCGACCTGACGCGCCTTTAGCAAAGGCTTCGACTGTCACGGGATTCGGACGTGGG    180
JX402858.1      AGTAACCGCATTGACGCACCCGTGTCTAAGGCCTCGACCGTAACTGGGTTTGGACGTGGG    119
KY315689.1      AGTAATCGCACTGACGCACCTGTGTCTAAAGCCTCGACTGTAACTGGGTTTGGACGTGGG    179
JN189992.2      AGTAATCGCACTGACGCACCTGTGTCCAAGGCCTCGACCGTAACTGGATTTGGACGTGGG    179
GU592791.1      AGTAATCGCACTGACGCACCTGTGTCTAAGGCCTCGACTGTAACTGGATTTGGACGTGGG    164
AF499774.1      AGTAATCGCACTGACGCACCTGTGTCTAAGGCCTCGACTGTAACTGGATTCGGACGTGGG    153
MG637439.1      AGTAACCGCACTGACGCACCTGTGTCTAAGGCCTCGACTGTGACTGGATTTGGACGTGGG    179
KU705815.1      AGTAATCGCACTGACGCACCTGTGTCTAAGGCCTCGACTGTGACTGGATTTGGACGTGGG    163
AF245004.1      AGTAATCGCACTGACGCGCCTGTGTCTAAGGCCTCGACTGTGACTGGATTTGGACGTGGG    167
JF412263.1      AGTAATCGCACTGACGCACCTGTGTCAAGGGCCTCGACTGTGACTGGATTTGGACGTGGG    179
MN309752.1      AGTAACCGCACTGACGCACCTGTGTCAAAGGCCTCGACTGTGACCGGATTTGGACGTGGG    179
KM588181.1      AGTAATCGCACTGACGCACCTGTGTCTAAGGCCTCGACTGTGACTGGATTTGGACGTGGG    176
FJ789784.1      AGTAATCGCACTGACGCACCTGTGTCTAAGGCCTCGACTGTAACTGGATTTGGACGTGGG    179
AF245003.1      AGCAATCGCACTGACGCACCTGTGTCTAAGGCCTCGACTGTAACTGGATTTGGACGTGGG    167
NC_008041.1     AGTAATCGCACTGACGCACCTGTGTCTAAGGCCTCGACTGTAACTGGATTTGGACGTGGG    179
AY744705.1      AGTAATCGCACTGACGCACCTGTGTCTAAGGCCTCGACTGTAACTGGATTTGGACGTGGG    179
                **      *    *** ** **   *   * *   ** *****   * ** ** ** ******* *

KY354702.2      ACCAATGATGTCCACATCTCGGGAATGTCACGGATCGCTCAGGCAGTTGTTCCAGCCGGG    230
KF386164.1      ACCAATGACGTCCATCTCTCGGGTATGTCGAGAATCTCCCAAGCAGTCCTCGCGGCCGGG    240
JX402858.1      ACCAATGACGTCCATCTCTCAGGTATGTCGAGAATCGCCCAAGCCGTCCTCCCAGCCGGG    179
KY315689.1      ACCAATGACGTCCATCTCTCAGGTATGTCGAGAATCTCCCAGGCCGTCCTCCCAGCCGGG    239
JN189992.2      ACCAATGACGTCCATCTCTCAGGTATGTCGAGAATCTCCCAGGCCGTCCTCCCAGCCGGG    239
GU592791.1      ACCAATGACGTCCATCTCTCAGGTATGTCGAGAATCTCCCAGGCCGTCCTCCCAGCCGGG    224
AF499774.1      ACCAATGAAGTCTATCTCTCAGGTATGTCGAGAATCTCCCAGGCCGTCCTCCCAGCCGGG    213
MG637439.1      ACCAATGACGTCCATCTCGCAGGTATGTCGAGAATCTCCCAGGCCGTCCTCCCAGCCGGG    239
KU705815.1      ACCAATGACGTCCATCTCTCAGGTATGTCGAGAATCTCCCAGGCCGTCCTCCCAGCCGGG    223
AF245004.1      ACCAATGACGTCCATCTCTCAGGTATGTCGAGAATCTCCCAGGCCGTCCTCCCAGCCGGG    227
JF412263.1      ACCAATGACGTCCATCTCTCAGGTATGTCGAGAATCCCCCAGGCCGTCCTCCCAGCCGGG    239
MN309752.1      ACCAATGACGTCCATCTCTCAGGTATGTCGAGAATCTCTCCAGGCCGTCCTCCCAGCCGGG    239
KM588181.1      ACCAATGACGTCCATCTCTCAGGTATGTCGAGAATCTCCCAGGCCGTCCTCCCAGCCGGG    236
FJ789784.1      ACCAATGACGTCCATCTCTCAGGTATGTCGAGAATCTCCCAGGCCGTCCTCCCAGCCGGG    239
AF245003.1      ACCAATGACGTCCATCTCTCAGGTATGTCGAGAATCTCCCAGGCCGTCCTCCCAGCCGGG    227
```

```
NC_008041.1      ACCAATGACGTCCATCTCTCAGGTATGTCGAGAATCTCCCAGGCCGTCCTCCCAGCCGGG    239
AY744705.1       ACCAATGACGTCCATCTCTCAGGTATGTCGAGAGTCTCCCAGGCCGTCCTCCCAGCCGGG    239
                 ******** *** *  ** * ** *****  *  ** * ** ** **  *  * ******

KY354702.2       ACAGGAACAGATGGAAAGATTGTCGTCGATTCCACAATCGTTCCAGAACTCCTGCCACGG    290
KF386164.1       GCAGGAACCGACGGATACATCGTCGTTGATTCGACCATCGTCCCCGACATCCTGCCACGA    300
JX402858.1       ACAGGAACAGACGGATACGTTGTTGTTGACGCAACCATCGTCCCCGACCTCCTGCCACGA    239
KY315689.1       ACAGGAACAGACGGATACGTTGTTGTTGACGCAACCATCGTCCCCGACCTCCTGCCACGA    299
JN189992.2       ACAGGAACAGACGGATACGTTGTTGTTGACGCAACCATCGTCCCCGACCTCCTGCCACGA    299
GU592791.1       ACAGGAACAGACGGATACGTTGTTGTTGACGCAACCATCGTCCCCGACCTCCTGCCACGA    284
AF499774.1       ACAGGAACAGACGGATACGTTGTTGTTGACGCAACCATCGTCCCCGACCTCCTGCCACGA    273
MG637439.1       ACAGGAACAGACGGATACGTTGTTGTTGACGCAACCATCGTTCCCGACCTCCTGCCACGA    299
KU705815.1       ACAGGAACTGACGGATACGTCGTTGTTGACGCAACCATCGTCCCCGACCTCCTGCCACGA    283
AF245004.1       ACAGGAACAGACGGATACGTTGTTGTTGACGCAACCATCGTCCCCGACCTCCTGCCACGA    287
JF412263.1       ACAGGAACAGACGGATACGTTGTTGTTGACGCAACCATCGTCCCCGACCTCCTGCCACGA    299
MN309752.1       ACAGGAACAGACGGATACGTTGTTGTTGACGCAACCATCGTCCCCGACCTCCTGCCACGA    299
KM588181.1       ACAGGAACTGACGGATACGTCGTTGTTGACGCAACCATCGTCCCCGACCTCCTGCCACGA    296
FJ789784.1       ACAGGAACGGACGGATACGTTGTTGTTGACGCAACCATCGTCCCCGACCTCCTGCCACGA    299
AF245003.1       ACAGGAACAGACGGATACGTTGTTGTTGATGCAACCATCGTCCCCGACCTCCTGCCACGA    287
NC_008041.1      ACAGGAACAGACGGATACGTTGTTGTTGACGCAACCATCGTCCCCGACCTCCTGCCACGA    299
AY744705.1       ACAGGAACAGACGGATACGTTGTTGTTGATGCAACCATCGTCCCCGACCTCCTGCCACGA    299
                  ******* ** *** *  * ** ** **  * ** ***** ** **  **********

     qPCR forward primer:CGCTGCTAGAATCTTCCA
KY354702.2       CTTGGACACGCTGCTCGAATCTTCCAGCGATACGCTGTTGAAACACTGGAGTTCGAAATT    350
KF386164.1       CTGGGACACGCTGCTAGAATCTTCCAGCGATACACTGTTGAGACATTGGAGTTCGAAGTT    360
JX402858.1       CTGGGACACGCTGCTAGAATCTTCCAGCGATACGCTGTTGAAACACTGGAGTTTGAAATT    299
KY315689.1       CTGGGACACGCTGCTAGAATCTTCCAGCGATACGCTGTTGAAACACTGGAGTTTGAAATT    359
JN189992.2       CTGGGACACGCTGCTAGAATCTTCCAGCGATACGCTGTTGAAACACTGGAGTTTGAAATT    359
GU592791.1       CTGGGACACGCTGCTAGAATCTTCCAGCGATACGCTGTTGAAACACTGGAGTTTGAAATT    344
AF499774.1       CTGGGACACGCTGCTAGAATCTTCCAGCGATACGCTGTTGAAACACTGGAGTTTGAAATT    333
MG637439.1       CTGGGACACGCTGCTAGAATCTTCCAGCGATACGCTGTTGAAACACTGGAGTTTGAAATT    359
KU705815.1       CTGGGACACGCTGCTAGAATCTTCCAGCGATACGCTGTTGAAACACTGGAGTTTGAAATT    343
AF245004.1       CTGGGACACGCTGCTAGAATCTTCCAGCGATACGCTGTTGAAACACTGGAGTTTGAAATT    347
JF412263.1       CTGGGACACGCCGCTAGAATCTTCCAGCGATACGCTGTTGAAACACTGGAGTTTGAAATC    359
MN309752.1       CTGGGACACGCTGCTAGAATCTTCCAGCGATACGCTGTTGAAACACTGGAGTTTGAAATT    359
KM588181.1       CTGGGACACGCTGCTAGAATCTTCCAGCGATACGCTGTTGAAACACTGGAGTTTGAAATT    356
FJ789784.1       CTGGGACACGCTGCTAGAATCTTCCAGCGATACGCTGTTGAAACACTGGAGTTTGAAATT    359
AF245003.1       CTGGGACACGCTGCTAGAATCTTCCAGCGATACGCTGTTGAAGCACTGGAGTTTGAAATT    347
NC_008041.1      CTGGGACACGCTGCTAGAATCTTCCAGCGATACGCTGTTGAAACACTGGAGTTTGAAATT    359
AY744705.1       CTGGGACACGCTGCTAGAATCTTCCAGCGATACGCTGTTGAAACACTGGAGTTTGAAATT    359
                 ** ******** *** ***************** *******  ** ******* *** *

KY354702.2       CAGCCAATGTGCCCCGCAAACACGGGCGGTGGTTACGTTGCTGGCTTCCTGCCTGATCCA    410
KF386164.1       CAGCCAATGTGCCCCGCAAACACGGGCGGTGGTTACGTTGCTGGCTTCCTGCCTAATCCA    420
JX402858.1       CAGCCAATGTGCCCCGCAAACACGGGCGGCGGTTACGTTGCTGGCTTCCTGCCTGATCCA    359
KY315689.1       CAGCCAATGTGCCCCGCAAACACGGGCGGTGGTTACGTTGCTGGCTTCTTGCCTAATCCA    419
JN189992.2       CAGCCAATGTGCCCCGCAAACACGGGCGGTGGTTACGTTGCTGGCTTCCTGCCTGATCCA    419
GU592791.1       CAGCCAATGTGCCCCGCAAACACGGGCGGTGGTTACGTTGCTGGCTTCCTGCCTGATCCA    404
AF499774.1       CAGCCAATGTGCCCCGCAAACACGGGCGGTGGTTACGTTGCTGGCTTCCTGCCTGATCCA    393
MG637439.1       CAGCCAATGTGCCCCGCAAACACGGGCGGTGGTTACGTTGCTGGCTTCCTGCCTGATCCA    419
KU705815.1       CAACCAATGTGCCCCGCAAACACGGGCGGTGGTTACGTTGCTGGCTTCCTGCCTGATCCA    403
AF245004.1       CAGCCAATGTGCCCCGCAAACACGGGCGGTGGTTACGTTGCTGGCTTCCTGCCTGATCCA    407
JF412263.1       CAGCCAATGTGCCCCGCAAACACGGGCGGTGGTTACGTTGCTGGCTTCCTGCCTGATCCA    419
MN309752.1       CAGCCAATGTGCCCCGCAAACACGGGCGGTGGTTACGTTGCTGGCTTCCTGCCTGATCCA    419
KM588181.1       CAGCCAATGTGCCCCGCAAACACGGGCGGTGGTTACGTTGCTGGCTTCCTGCCTGATCCA    416
FJ789784.1       CAGCCAATGTGCCCCGCAAACACGGGCGGTGGTTACGTTGCTGGCTTCCTGCCTGATCCA    419
AF245003.1       CAGCCAATGTGCCCCGCAAACACGGGCGGTGGTTACGTTGCTGGCTTCCTGCCTGATCCA    407
NC_008041.1      CAGCCAATGTGCCCCGCAAACACGGGCGGTGGTTACGTTGCTGGCTTCCTGCCTGATCCA    419
AY744705.1       CAGCCAATGTGCCCCGCAAACACGGGCGGTGGTTACGTTGCTGGCTTCCTGCCTGATCCA    419
                 ** ************************** ****************** ***** *****

                                                      qPCR reverse primer:GCCAAA
KY354702.2       ACTGACAACGACCACACCTTCGATGCGCTTCAAGCAACTCGTGGTGCAGTCGTCGCCAAA    470
KF386164.1       ACTGACAACGACCACACCTTCGACGCGCTTCAAGCGACTCGTGGTGCCGTTGTTGGCAAA    480
JX402858.1       ACTGACAACGATCACACCTTCGACGCGCTTCAAGCAACTCGTGGTGCAGTCGTTGCCAAA    419
KY315689.1       ACTGACAACGATCACACCTTCGACGCGATTCAAGCAACTCGTGGTGCAGTCGTTGCCAAA    479
JN189992.2       ACTGACAACGATCACACCTTCGACGCACTTCAAGCAACTCGTGGTGCAGTCGTTGCCAAA    479
GU592791.1       ACTGACAACGATCACACCTTCGACGCGCTTCAAGCAACTCGTGGTGCAGTCGTTGCCAAA    464
AF499774.1       ACTGACAACGATCACACCTTCGACGCGCTTCAAGCAACTCGTGGTGCAGTCGTTGCCAAA    453
MG637439.1       ACTGACAACGATCACACCTTCGACGCGCTTCAAGCAACTCGTGGTGCAGTCGTTGCCAAA    479
KU705815.1       ACTGACAACGACCACACCTTCGACGCGCTTCAAGCAACTCGTGGTGCAGTCGTTGCCAAA    463
AF245004.1       ACTGACAACGACCACACCTTCGACGCGCTTCAAGCAACTCGTGGTGCAGTCGTTGCCAAA    467
JF412263.1       ACTGACAACGACCACACCTTCGATGCGCTTCAAGCAACTCGTGGTGCAGTCGTTGCCAAA    479
MN309752.1       ACTGACAACGACCACACCTTCGACGCGCTTCAAGCAACTCGTGGTGCAGTCGTTGCCAAA    479
KM588181.1       ACTGACAACGACCACACCTTCGACGCGCTTCAAGCAACTCGTGGTGCAGTCGTTGCCAAA    476
FJ789784.1       ACTGACAACGACCACACCTTCGACGCGCTTCAAGCAACTCGTGGTGCAGTCGTTGCCAAA    479
AF245003.1       ACTGACAACGATCACACCTTCGACGCGCTTCAAGCAACTCGTGGTGCAGTCGTTGCCAAA    467
NC_008041.1      ACTGACAACGATCACACCTTCGACGCGCTTCAAGCAACTCGTGGTGCAGTCGTTGCCAAA    479
```

```
AY744705.1      ACTGACAACGATCACACCTTCGACGCGCTTCAAGCAACTCGTGGTGCAGTCGTTGCCAAA    479
                *********** *********** **  ******* *********** ** ** * ****

                TGGTGGGAAAG
KY354702.2      TGGTGGGAAAGCCGAACGGTCCGGCCCCAGTATACTCGAACGCTTCTCTGGACCTCAACC    530
KF386164.1      TGGTGGGAGAGCAGGACAGTACGTCCACAGTACACTCGAACCATGCTCTGGACCTCAACA    540
JX402858.1      TGGTGGGAAAGCAGAACTGTCCGACCACAGTACACCCGCACGCTCCTTTGGACCTCATCG    479
KY315689.1      TGGTGGGAAAGCAGAACAGTCCGACCTCAGTACACCCGTACGCTCCTCTGGACCTCGTCG    539
JN189992.2      TGGTGGGAAAGCAGAACAGTCCGACCGCAGTTCACTCGCACGCTCCTCTGGACCTCGTCG    539
GU592791.1      TGGTGGGAAAGCAGAACAGTCCGACCTCAGTACACCCGCACGCTCCTCTGGACCTCGTCG    524
AF499774.1      TGGTGGGAAAGCAGAACAGTCCGACCTCAGTACACCCGCACGCTCCTCTGGACCTCGTCG    513
MG637439.1      TGGTGGGAAAGCAGAACAGTCCGACCTCAGTACACCCGCACGCTCCTCTGGACCTCGTCG    539
KU705815.1      TGGTGGGAAAGCAGAACAGTCCGACCTCAGTACACCCGTACGCTCCTCTGGACCTCGTCG    523
AF245004.1      TGGTGGGAAAGCAGAACAGTCCGACCTCAGTACACCCGCACGCTCCTCTGGACCTCGTCG    527
JF412263.1      TGGTGGGAAAGCAGAACAGTCCGACCTCAGTACACCCGCACGCTCCTCTGGACCTCGTCG    539
MN309752.1      TGGTGGGAAAGCAGAACAGTCCGACCCCAGTACACCCGCACGCTCCTCTGGACCTCGTCG    539
KM588181.1      TGGTGGGAAAGCAGAACAGTCCGACCTCAGTACACCCGTACGCTCCTCTGGACCTCGTCG    536
FJ789784.1      TGGTGGGAAAGCAGTACAGTCCGACCTCAGTACACCCGCACGCTCCTCTGGACCTCGTCG    539
AF245003.1      TGGTGGGAAAGCAGAACAGTCCGACCTCAGTACACCCGCACGCTCCTCTGGACCTCGACG    527
NC_008041.1     TGGTGGGAAAGCAGAACAGTCCGACCTCAGTACACCCGCACGCTCCTCTGGACCTCGTCG    539
AY744705.1      TGGTGGGAAAGCAGAACAGTCCGACCTCAGTACACCCGCACGCTCCTCTGGACCTCGTCG    539
                ******** *** * ** ** ** ** ****  ** ** **  * ** ********  *

KY354702.2      GGGAAGGAGCAGCGATTGACGTCACCTGGCCGGCTGGTACTCCTGTGTGTTGGCAGCAAC    590
KF386164.1      GGAAAGGAGCAGCGTTTGACTTCACCTGGACGCTTCATTCTCCTCTGTGTCGGCAGCAAC    600
JX402858.1      GGAAAGGAGCAGCGTCTCACGTCACCTGGTCGGCTGATACTCCTGTGTGTCGGCAACAAC    539
KY315689.1      GGAAAGGAGCAGCGTCTCACGTCACCTGGTCGGCTGATACTCCTGTGTGTCGGCGACAAC    599
JN189992.2      GGAAAGGAGCAGCGTCTTACATCACCTGGTCGGCTGATACTCCTGTGTGTCGGCAACAAC    599
GU592791.1      GGAAAGGAGCAGCGTCTCACGTCACCTGGTCGGCTGATACTCCTGTGTGTCGGCAACAAC    584
AF499774.1      GGAAAGGAGCAGCGTCTCATGTCACCTGGTCGGCTGATACTCCTGTGTGTCGGCAACAAC    573
MG637439.1      GGAAAGGAGCAGCGTCTCACGTCACCTGGTCGGCTGATACTCCTGTGTGTCGGCAACAAC    599
KU705815.1      GGAAAGGAGCAGCGTCTCACGTCACCTGGTCGGCTGATACTCCTGTGTGTCGGCAACAAC    583
AF245004.1      GGAAAGGAGCAGCGTCTCACGTCACCTGGTCGGCTGATACTCCTGTGTGTCGGCAACAAC    587
JF412263.1      GGAAAGGAGCAGCGTCTCACGTCACCTGGTCGGCTGATACTCCTGTGTGTCGGCAACAAC    599
MN309752.1      GGAAAGGAGCAGCGTCTCATGTCACCTGGTCGGCTGATACTCCTGTGTGTCGGCAACAAC    599
KM588181.1      GGAAAGGAGCAGCGTCTCACGTCACCTGGTCGGCTGATACTCCTGTGTGTCGGCAACAAC    596
FJ789784.1      GGAAAGGAGCAGCGTCTCACGTCACCTGGTCGGCTGATACTCCTGTGTGTCGGCAACAAC    599
AF245003.1      GGAAAGGAGCAGCGTCTCACGTCACCTGGTCGGCTGATACTCCTGTGTGTCGGCAACAAC    587
NC_008041.1     GGAAAGGAGCAGCGTCTCACGTCACCTGGTCGGCTGATACTCCTGTGTGTCGGCAACAAC    599
AY744705.1      GGAAAGGAGCAGCGTCTCACGTCACCTGGTCGGCTGATACTCCTGTGTGTCGGCAACAAC    599
                ** ***********  * *  ******** **  *  * ***** ***** ***  ****

KY354702.2      ACTGACGTTGTCAACGTGTCAGTCATGTGTCGCTGGAGCGTTCGCCTTAGTGTCCCGTCC    650
KF386164.1      ACTGACGTGGTTAACGTGTCGGTGTTATGTCGCTGGAGTGTGCGCCTCAGTGTCCCATCT    660
JX402858.1      ACTGACGTGGTTAACGTTTCAGTGCTGTGTCGCTGGAGTGTTCGACTGAGCGTTCCGTCC    599
KY315689.1      ACTGATGTGGTCAACGTGTCAGTGCTGTGTCGCTGGAGTGTTCGACTGAGCGTTCCATCT    659
JN189992.2      ACTGACGTGGTTAACGTGTCGGTGCTGTGTCGCTGGAGTGTTCGACTGAGCGTTCCGTCT    659
GU592791.1      ACTGATGTGGTCAACGTGTCAGTGCTGTGTCGCTGGAGTGTTCGACTGAGCGTTCCATCT    644
AF499774.1      ACTGATGTGGTCAACGTGTCAGTGCTGTGTCGCTGGAGTGTTCGACTGAGCGTTCCATCT    633
MG637439.1      ACTGACGTGGTCAACGTGTCGGTGCTGTGTCGCTGGAGTGTTCGACTGAGCGTTCCATCT    659
KU705815.1      ACTGATGTGGTCAACGTGTCAGTGCTGTGTCGCTGGAGTGTTCGACTGAGCGTTCCATCT    643
AF245004.1      ACTGATGTGGTCAACGTGTCAGTGCTGTGTCGCTGGAGTGTTCGACTGAGCGTTCCATCT    647
JF412263.1      ACTGATGTGGTCAACGTGTCAGTACTGTGTCGCTGGAGTGTTCGACTGAGCGTTCCATCT    659
MN309752.1      ACTGATGTGGTCAACGTGTCAGTACTGTGTCGCTGGAGTGTTCGACTGAGCGTTCCATCT    659
KM588181.1      ACTGATGTGGTCAACGTGTCAGTGCTGTGTCGCTGGAGTGTTCGACTGAGCGTTCCATCT    656
FJ789784.1      ACCGATGTGGTCAACGTGTCGGTGCTATGTCGCTGGAGTGTTCGATTGAGCGTTCCATCT    659
AF245003.1      ACTGATGTGGTCAACGTGTCAGTGCTGTGTCGCTGGAGTGTTCGACTGAGCGTTCCATCT    647
NC_008041.1     ACTGATGTGGTCAACGTGTCAGTGCTGTGTCGCTGGAGTGTTCGACTGAGCGTTCCATCT    659
AY744705.1      ACTGATGTGGTCAACGTGTCAGTGCTGTGTCGCTGGAGTGTTCGACTGAGCGTTCCATCT    659
                ** ** ** ** ***** ** **  * *********** ** **  * ** ** ** **

KY354702.2      CTTGAGACACCTGAGGACACCACCGCTCCAATTACTACCCAGGCGCCACTCCACAACGAT    710
KF386164.1      CTCGAGACGCCTGAGGACACAGCTGCACCGATTTTGTCACAGGGTCCCCTCTACAACGAT    720
JX402858.1      CTTGAAACACCTGAAGAGACCAACGCTCCCATCATGACACAGGGTCCCCTGTACAACGAT    659
KY315689.1      CTTGAGACACCTGAAGAGACCACCGCTCCCATCATGACACAAGGTTCCCTGTACAACGAT    719
JN189992.2      CTTGAAACACCTGAAGAGACCACCGCTCCCATCATGACACAAGGTCCCCTGTACAATGAT    719
GU592791.1      CTTGAGACACCTGAAGAGACCACCGCTCCCATCATGACACAAGGTTCCCTGTACAACGAT    704
AF499774.1      CTTGAGACACCTGAAGAGACCACCGCTCCCATCATGACACAAGGTTCCCTGTACAACGAT    693
MG637439.1      CTTGAGACACCTGAAGAGACCACCGCTCCCATCATGACACAAGGTTCCCTGTACAACGAT    719
KU705815.1      CTTGAGACACCTGAAGAGACCACCGCTCCCATCATGACACAAGGTTCCCTGTACAACGAT    703
AF245004.1      CTTGAGACACCTGAAGAGACCACCGCTCCCATCATGACACAAGGTTCCCTGTACAACGAT    707
JF412263.1      CTTGAGACACCTGAGGAGACAACCGCTCCCATCATGACACAAGGTTCCCTGTACAACGAT    719
MN309752.1      CTTGAGACACCTGAGGAGACAACCGCTCCCATCATGACACAAGGTTCCCTGTACAACGAT    719
KM588181.1      ATTGAGACACCTGAAGAGACCACCGCACCCATCATGACACAAGGTTCCCTGTACAACGAT    716
FJ789784.1      CTTGAGACACCTGAAGAGACCACCGCTCCCATCATGACACAAGGTTCCCTGTACAACGAT    719
AF245003.1      CTTGAGACACCTGAAGAGACCACCGCTCCCATCATGACACAAGGTTCCCTGTACAACGAT    707
NC_008041.1     CTTGAGACACCTGAAGAGACCACCGCTCCCATCATGACACAAGGTTCCCTGTACAACGAT    719
```

```
AY744705.1        CTTGAGACACCTGAAGAGACCACCGCTCCCATCATGACACAAGGTTCCCTGTACAACGAT    719
                   * ** ** ***** ** **    ** ** **     * ** *   * **  **** ***

KY354702.2        TCCATTAACAACGGTTACACTGGATTTCGGTCCATTCTTTTGGGCTCGACCCAACTCGAC    770
KF386164.1        TCCCTTGCCACAA------CTGACTTCAAATCCATCCTCCTGGGTTCCACACAGCTGGAC    774
JX402858.1        TCCCTCGCTGCAC------ATGACTTCAAGTCCATCCTCCTAGGATCCACACCACTGGAC    713
KY315689.1        TCCCTTTCCACAA------ATGACTTCAAGTCCATCCTCCTAGGATCCATACCACTGGAC    773
JN189992.2        TCCCTCGCTGCAA------CTGACTTCAAGTCCATCCTCCTAGGATCCACAGCACTGGAC    773
GU592791.1        TCCCTTTCCACAA------ATGACTTCAAGTCCATCCTCCTAGGATCCACACCACTGGAC    758
AF499774.1        TCCCTTTCCACAA------CTGACTTCAAGTCCATCCTTCTAGGATCCACACCACTGGAC    747
MG637439.1        TCCCTTTCCACAA------ATGACTTCAAGTCCATCCTCCTAGGATCCACGCCACTGGAC    773
KU705815.1        TCCCTTTCCACAA------ATGACTTCAAGTCCATCCTCCTAGGATCCACGCCACTGGAT    757
AF245004.1        TCCCTTTCCACAA------ATGACTTTAAGTCCATCCTCCTAGGATCCACACCACTGGAC    761
JF412263.1        TCCCTTTCCACAA------ATGACTTCAAGTCCATCCTCCTAGGATCCACACCACTGGAC    773
MN309752.1        TCCCTTTCCACAA------ATGACTTCAAGTCCATCCTCCTAGGATCCACACCACTGGAC    773
KM588181.1        TCCCTTTCCACAA------CTGACTTCAAGTCCATCCTCCTAGGATCCACACCACTGGAT    770
FJ789784.1        TCCCTTTCCACAA------ATGACTTCAAGTCCATCCTCCTAGGATCCACACCACTGGAC    773
AF245003.1        TCCCTTTCCACAA------ATGACTTCAAATCCATCCTCCTAGGATCCACACCACTGGAC    761
NC_008041.1       TCCCTTTCCACAA------ATGACTTCAAGTCCATCCTCCTAGGATCCACACCACTGGAC    773
AY744705.1        TCCCTTTCCACAA------ATGACTTCAAGTCCATCCTCCTAGGATCCACACCACTGGAC    773
                  *** *               **  **    ***** **  * ** ** *     ** **

KY354702.2        CTCGCTCCTGCAAACGCTGTCTTTGTCACTGACAAACCGTTGCCCATTGATTACAGTCTT    830
KF386164.1        ATATCCCCGGACGGCGCCATCTTCCAGATGGACCGCCCGCTGTCCATTGATTACAAGCTG    834
JX402858.1        ATAGCCCCTGACGGTGCAGTCTACCAGCTGGACCGTCCGCTGTCCATCGATTACAGCCTT    773
KY315689.1        ATTGCCCCTGATGGAGCAGTCTTCCAGCTGGACCGCCCGCTGTCCATTGACTACAGCCTT    833
JN189992.2        ATTGCTCCTGACGGAGCAATCTTCCAGCTGGACCGTCCGTTGTCCATTGACTACAACCTT    833
GU592791.1        ATTGCCCCTGATGGAGCAGTCTTCCAGCTGGACCGTCCGCTGTCCATTGACTACAGCCTT    818
AF499774.1        ATTGCCCCTGATGGAGCAGTCTTCCAGCTGGACCGTCCGCTGTCCATTGACTACACCCTT    807
MG637439.1        ATTGCCCCTGATGGAGCAGTCTTCCAGCTGGACCGTCCGCTGTCCATCGACTACAGCCTT    833
KU705815.1        ATTGCCCCTGATGGAGCAGTCTTCCAGCTGGACCGTCCGCTGTCCATCGACTACAGCCTT    817
AF245004.1        ATTGCCCCTGATGGAGCAGTCTTCCAGCTGGATCGACCGCTGTCCATTGACTACAGCCTT    821
JF412263.1        ATTGCCCCTGATGGAGCAGTCTTCCAGCTGGACCGTCCGCTGTCCATTGACTACAGCCTT    833
MN309752.1        ATTGCCCCTGATGGAGCAGTCTTCCAGCTGGACCGTCCGCTGTCCATTGACTACAGCCTT    833
KM588181.1        ATTGCCCCTGATGGAGCAGTCTTCCAGCTGGACCGTCCGCTGTCCATTGACTACAGCCTT    830
FJ789784.1        ATTGCCCCTGATGGAGCAGTCTTCCAGCTGGACCGTCCGCTATCCATTGACTACAGCCTT    833
AF245003.1        ATTGCCCCTGATGGAGCAGTCTTCCAGCTGGACCGTCCGCTGTCCATTGACTACAGCCTT    821
NC_008041.1       ATTGCCCCTGATGGAGCAGTCTTCCAGCTGGACCGTCCGCTGTCCATTGACTACAGCCTT    833
AY744705.1        ATTGCCCCTGATGGAGCAGTCTTCCAGCTGGACCGTCCGCTGTCCATTGACTACAGCCTT    833
                   *  * ** *     **  ***        **    *** *  **** ** ****  **

KY354702.2        GGAGTGGGCGACGTCGACCGGGCCGTATACTGGCACCTGCGGAAGAAAGCTGGAGACACT    890
KF386164.1        GGAACCGGAGATGTTGACCGTGCCGTCTATTGGCACCTCAAGAAGTTTGCGGGCACTGCC    894
JX402858.1        GGAACTGGAGATGTTGACCGTGCCGTTTATTGGCACCTCAAGAAGTTTGCTGGAACTGCC    833
KY315689.1        GGAACTGGAGATGTTGACCGTGCTGTTTACTGGCACCTCAAGAAGTTTGCTGGAAATGCT    893
JN189992.2        GGAGTTGGAGATGTTGACCGTGCCGTCTACTGGCACCTCAAGAAGTTTGCTGGCACCGCT    893
GU592791.1        GGAACTGGAGATGTTGACCGTGCTGTTTATTGGCACCTCAAGAAGTTTGCTGGAAATGCT    878
AF499774.1        GGAACTGGAGATGTTGACCGTGCTGTTTATTGGCACCTCAAGAAGTTTGCTGGAAATGCT    867
MG637439.1        GGAACTGGAGATGTTGACCGTGCTGTTTACTGGCACCTCAAGAAGTTTGCTGGAAATGCT    893
KU705815.1        GGAACTGGAGATGTTGATCGTGCTGTTTATTGGCACCTCAAGAAGTTTGCTGGAAATGCT    877
AF245004.1        GGAACTGGAGATGTTGACCGTGCTGTTTACTGGCACCTCAAGAAGTTTGCTGGAAATGCT    881
JF412263.1        GGAACTGGAGATGTTGATCGAGCTGTCTATGGGCACCTCAAGAAGTTTGCTGGAAATGCT    893
MN309752.1        GGAACTGGAGATGTTGATCGAGCTGTCTATTGGCACCTCAAGAAGTTTGCTGGAAATGCT    893
KM588181.1        GGAACTGGAGATGTTGATCGTGCTGTTTATTGGCACCTCAAGAAGTTTGCTGGAAATGCT    890
FJ789784.1        GGAACTGGAGATGTTGACCGTGCTGTTTACTGGCACCTCAAGAAGTTTGCTGGAAATGCT    893
AF245003.1        GGAACTGGAGATGTTGACCGTGCTGTTTACTGGCACCTCAAGAAGTTTGCTGGAAATGCT    881
NC_008041.1       GGAACTGGAGATGTTGACCGTGCTGTTTATTGGCACCTCAAGAAGTTTGCTGGAAATGCT    893
AY744705.1        GGAACTGGAGATGTTGACCGTGCTGTTTACTGGCACCTCAAGAAGTTTGCTGGAAATGCT    893
                  ***   ** ** ** ** ** ** ** **  *******   ****   ** **     *

KY354702.2        CAGGTACCTGCTGGGTACTTTGACTGGGGACTGTGGGATGACTTCAACAAGACATTCACA    950
KF386164.1        GCCACACCGGMTGGCTGGTTTCGCTGGGGCATCTGGGACAACTTCAACAAAACATTTACC    954
JX402858.1        ACCACACCTGCAGGTTGGTTTCGCTGGGGCGTCTGGGACAACTTTAACAAAACGTTCACA    893
KY315689.1        AGCACACCTGCAGGCTGGTTTCGCTGGGGCATCTGGGACAACTTCAACAAGACGTTCACA    953
JN189992.2        GCCACACCTGCAGGCTGGTTTCGCTGGGGCATCTGGGACAACTTCAACAAAACGTTCACA    953
GU592791.1        GGCACACCTGCAGGCTGGTTTCGCTGGGGCATCTGGGACACTTCAACAAGACGTTTCGCA    938
AF499774.1        AGCACACCTGCAGGCTGGTTTCGCTGGGGCATCTGGGACAACTTCAACAAGACGTTCACA    927
MG637439.1        GGCACACCTGCAGGCTGGTTTCGCTGGGGCATCTGGGACAACTTCAACAAGACGTTCACA    953
KU705815.1        GGCACACCTGCAGGCTGGTTTCGCTGGGGCATCTGGGACAACTTCAATAAGACGTTCACA    937
AF245004.1        GGCACACCTGCAGGCTGGTTTCGCTGGGGCATCTGGGACAACTTCAACAAGACGTTCACA    941
JF412263.1        GGCACACCTGCAGGCTGGTTTCGCTGGGGCATCTGGGACAACTTCAACAAGACGTTCACA    953
MN309752.1        GGCACACCTGCAGGCTGGTTTCGCTGGGGCATCTGGGACAACTTCAACAAGACGTTCACA    953
KM588181.1        GGCACACCTGCAGGCTGGTTTCGCTGGGGCATCTGGGACAACTTCAATAAGACGTTCACA    950
FJ789784.1        GGCACACCTGCAGGCTGGTTTCGCTGGGGCATCTGGGACAACTTCAACAAGACGTTCACA    953
AF245003.1        GGCACACCTGCAGGCTGGTTTCGCTGGGGCATCTGGGACAACTTCAACAAGACGTTCACA    941
NC_008041.1       GGCACACCTGCAGGCTGGTTTCGCTGGGGCATCTGGGACAACTTCAACAAGACGTTCGCA    953
AY744705.1        GGCACACCTGCAGGCTGGTTTCGCTGGGGCATCTGGGACAACTTCAACAAGACGTTCACA    953
```

```
                      *** *  ** *  ***  ******  * *****    *  *  *     **  *

KY354702.2        GTTGGGGCGCCCTACTACTCCGACCAGCAACCACGGCAAATCTTGCTGCCGGCTGGCACG    1010
KF386164.1        GACGGTATCGCTTACTATTCTGACGAGCAGCCACGCCAGATCCTGCTCCCGGTAGGCACT    1014
JX402858.1        GATGGCGTTGCTTACTACCCTGACGAGCAGCCTCGTCAAATTCTCCTGCCCGTTGGCACT    953
KY315689.1        GATGGCGTTGCTTACTACCCTGATGAGCAGCCTCGTCAAATCCTGCTGCCTGTTGGCACT    1013
JN189992.2        GATGGCGTTGCTTACTACTCCGATCAGCAGCCTCGTCAAATCCTCCTGCCTGTTGGCACT    1013
GU592791.1        GATGCCGTTGGCTACTACTCTGATGCGCAGCCTCGTCAAATCCTGCTGCTGTTGGCCACT    998
AF499774.1        GATGGCGTTGCCTACTACTCTGATGAGCAGCCCCGTCAAATCCTGCTGCCTGTTGGCACT    987
MG637439.1        GATGGCGTTGCTTACTACTCTGATGAGCAGCCCCGTCAAATCCTGCTGCCTGTTGGCACT    1013
KU705815.1        GATGGCGTTGCCTACTACTCTGATGAGCAGCCCCGTCAAATCCTGCTGCCTGTTGGCACT    997
AF245004.1        GATGGCGTTGCCTACTACTCTGATGAGCAGCCCCGTCAAATCCTGCTGCCTGTTGGCACT    1001
JF412263.1        GATGGCGTTGCCTACTACTCTGATGAGCAGCCCCGTCAAATCCTGCTGCCTGTTGGCACT    1013
MN309752.1        GATGGCGTTGCCTACTACTCTGATGAGCAGCCCCGTCAAATCCTGCTGCCTGTTGGCACT    1013
KM588181.1        GATGGCGTTGCCTACTACTCTGATGAGCAGCCCCGTCAAATCCTGCTGCCTGTTGGCACT    1010
FJ789784.1        GATGGCGTTGCTTACTACTCTGATGAGCAGCCTCGTCAAATCCTGCTGCCTGTTGGCACT    1013
AF245003.1        GATGGCGTTGCCTACTACTCTGATGAGCAGCCCCGTCAAATCCTGCTGCCTGTTGGCACT    1001
NC_008041.1       GATGGCGTTGCCTACTACTCTGATGAGCAGCCTCGTCAAATCCTGCTGCCTGTTGGCACT    1013
AY744705.1        GATGGCGTTGCCTACTACTCTGATGAGCAGCCCCGTCAAATCCTGCTGCCTGTTGGCACT    1013
                  *  *        *****  * **   *** ** ** ** **  * ** *     * ***
                      Conventional PCR reverse primer: ATCCGGTTCCCTAGTGC
KY354702.2        CTCTTCACCCGTGTTGACTCGGAAAACTAACCGGGTCATCCGGATCCCTAGTGCGTATCG    1070
KF386164.1        ACCTTCACCAGGGTCGATGCGGGAAACTAACCGGGTCATCCGGTTCCCTAGTGCGTATCG    1074
JX402858.1        GTCTGCACCAGGGTTGATTCGGAAAACTAACCGGGTCATCCGGTTCCCTAGTGCGTATCG    1013
KY315689.1        GTCTGCACCAGGGTTGACTCGGAAAACTAACCGGGTCATCCGGTTCCCTAGTGCGTATCG    1073
JN189992.2        GTCTGCACCAGGGTTGACTCGGAAAACTAACCGGGTCATCCGGTTCCCTAGTGCGTATCG    1073
GU592791.1        GTCTGCACCAGGGTTGACTCGGAAAACTAACCGGGTCATCCGGTTCCCTAGTGCGTATCG    1058
AF499774.1        GTCTGCATCAGGGTTGACTCGGAAAACTAACCGGGTCATCCGGTTCCCTAGTGCGTATCG    1047
MG637439.1        GTCTGCACCAGGGTTGACTCGGAAAACTAACCGGGTCATCCGGTTCCCTAGTGCGTATCG    1073
KU705815.1        GTCTGCACCAGGGTTGACTCGGAAAACTAACCGGGTCATCCGGTTCCCTAGTGCGTATCG    1057
AF245004.1        GTCTGCACCAGGGTTGACTCGGAAAACTAACCGGGTCATCCGGTTCCCTAGTGCGTATCG    1061
JF412263.1        GTCTGCACCAGGGTTGACTCGGAAAACTAACCGGGTCATCCGGTTCCCTAGTGCGTATCG    1073
MN309752.1        GTCTGCACCAGGGTTGACTCGGAAAACTAACCGGGTCATCCGGTTCCCTAGTGCGTATCG    1073
KM588181.1        GTCTGCACCAGGGTTGACTCGGAAAACTAACCGGGTCATCCGGTTCCCTAGTGCGTATCG    1070
FJ789784.1        GTCTTCACCCGTGTTGACTCGGGAAACTAACCGGGTCATCCGGTTCCCTAGTGCGTATCG    1073
AF245003.1        GTCTGCACCAGGGTTGACTCGGAAAACTAACCGGGTCATCCGGTTCCCTAGTGCGTATCG    1061
NC_008041.1       GTCTGCACTAGGGTTGACTCGGAAAACTAACCGGGTCATCCGGTTCCCTAGTGCGTATCG    1073
AY744705.1        GTCTGCACCAGGGTTGACTCGGAAAACTAACCGGGTCATCCGGTTCCCTAGTGCGTATCG    1073
                    ** **    * ** **   *** ******************** ****************

KY354702.2        TGGATGACCAATTTGAGAAATTGATTATGGCACTAACCACTATCAAAATT----------    1120
KF386164.1        TTGATGACCAATTTGAACAATTAATTAATGCACTAAGTATTATAACTAATGAAATACAAA    1134
JX402858.1        TTGATGACCAATTTGAACAATTGATTAAAGCACTAACATATATAAATGAAGAAATACAAA    1073
KY315689.1        TTGATGACCAATTTGAACAATTGATTAAAGCACTAACAAATATAAATAAAGAAATACAAA    1133
JN189992.2        TTGATGACCAATTTGAACAATTGATTAAAGCACTAACAAACATAAACAAAGAAATACAAA    1133
GU592791.1        TTGATGACCAATTTGAACAATTGATTAAAGCACTAACAAATATAAATAAAGAAATACAAA    1118
AF499774.1        TTGATGACCAATTTGAACAATTGATTAAAGCACTAACAAACATAAATAAAGAAATACAAA    1107
MG637439.1        TTGATGACCAATTTGAACAATTGATTAAAGCACTAACAAATATAAATAAAGAAATAGAAA    1133
KU705815.1        TTGATGACCAATTTGAACAATTGATTAAAGCACTAACAAATATAAATAAAGAAATACAAA    1117
AF245004.1        TTGATGACCAATTTGAACAATTGATTAAAGCACTAACGAACATAAATAAAGAAATACAAA    1121
JF412263.1        TTGATGACCAATTTGAACAATTGATTAAAGCACTAAAGAATATAAATAAAGAAATACAAA    1133
MN309752.1        TTGATGACCAATTTGAACAATTGATTAAAGCACTAACAAATATAAATGAAGAAATACAAA    1133
KM588181.1        TTGATGACCAATTTGAACAATTGATTAAAGCACTAACAAATATAAATAAAGAAATACAAA    1130
FJ789784.1        TTGATGACCAATTTGAACAATTGATTAAAGCACTAACAAACATAAATAAAGAAATACAAA    1133
AF245003.1        TTGATGACCAATTTGAACAATTGATTAAAGCACTAACAAATATAAATAAAGAAATACAAA    1121
NC_008041.1       TTGATGACCAATTTGAACAATTGATTAAAGCACTAACAAATATAAATAAAGAAATACAAA    1133
AY744705.1        TTGATGACCAATTCGAACAATTGATTAAAGCACTAACAAATATAAATAAAGAAATACAAA    1133
                  * *********** **  **** ****  *******     ** *

KY354702.2        -----------GAAATTGACAATAACAAGAGCGAAATTGAAGCTATCGCTAACAAATTAA    1169
KF386164.1        TAAACAAAACCGAAATTGGCAAGAATAAGAGCGAAATTGACGCTATCGCCACTAAATTAA    1194
JX402858.1        CAAACAAAATTGAAATTGGAAAGAACAGAAGCGAAATTGAATCACTCGCTGGCAAATTAA    1133
KY315689.1        CAAACAAAACTGAAATTGGAAAGAATAGAAGCGAAATTGAATCACTCGCTAGCAAATTAA    1193
JN189992.2        TAAATAAAACTGAAATTGGAAAGAATAGAAGCGAAATTGAATCACTCGCTAGCAAATTAA    1193
GU592791.1        CAAACAAAACTGAAATTGGAAAGAATAGAAGCGAAATTGAATCACTCGCTAGCAAATTAA    1178
AF499774.1        CAAACAAAACTGAAATTGGAAAGAATAGAAGCGAAATTGAATCACTCGCTAGCAAATTAA    1167
MG637439.1        CAAACAAAACTGAAATTGGAAAGAACAGAAGCGAAATTGAATCACTCGCTAGCAAATTAA    1193
KU705815.1        CAAACAAAACTGAAATTGGAAAGAATAGAAGCGAAATTGAATCACTCGCTAGCAAATTAA    1177
AF245004.1        CAAACAAAACTGAAATTGGAAAGAATAGAAGCGAAATTGAATCACTCGCTAGCAAATTAA    1181
JF412263.1        CAAACAAAACTGAAATTGGAAAGAATAGAAGCGAAATTGAATCACTCGCTAGCAAATTAA    1193
MN309752.1        CAAACAAAACTGAAATTGGAAAGAATAGAAGCGAAATTGAATCACTCGCTAGCAAATTAA    1193
KM588181.1        CAAACAAAACTGAAATTGGAAAGAATAGAAGCGAAATTGAATCACTCGCTAGCAAATTAA    1190
FJ789784.1        CAAACAAAACTGAAATTGGAAAGAATAGAAGCGAAATTGAATCACTCGCTAGCAAATTAA    1193
AF245003.1        CAAACAAAACTGAAATTGGAAAGAATAGAAGCGAAATTGAATCACTCGCTAGCAAATTAA    1181
NC_008041.1       CAAACAAAACTGAAATTGGAAAGAATAGAAGCGAAATTGAATCACTCGCTAGCAAATTAA    1193
AY744705.1        CAAACAAAACTGAAATTGGAAAGAATAGAAGCGAAATTGAACCACTCGCTAGCAAATTAA    1193
                             *******  ** ** *  ***********  *  ****    *******
```

```
KY354702.2      ACGACAAAGCACCCAAAGAGGGCTCGATTGCTATTGTTGGTACCATTGACGGCGTACCTG    1229
KF386164.1      AAGACAAAGCCCCCAAGGAGGGTGCTATTGCTATTGTTGGTACCATTGACGGCGTACCGG    1254
JX402858.1      ACGACAAAGCACCCAAGGAGGGTGCGATTGCTATTGTTGGTACCCTTGATGGCGTACCGG    1193
KY315689.1      ACGACAAAGCACCCAAGGAGGGTGCGATTGCTATTGTTGGTACCCTTGACGGCGTACCGG    1253
JN189992.2      ATGACAAAGCACCCAAGGAGGGTGCGATTGCTATTGTTGGTACCCTTGACGGCGTACCGG    1253
GU592791.1      ACGACAAAGCACCCAAGGAGGGTGCGATTGCTATTGTTGGTACCCTTGACGGCGTACCGG    1238
AF499774.1      ACGACAAAGCACCCAAGGAGGGTGCGATTGCTATTGTTGGTACCCTTGACGGCGTACCGG    1227
MG637439.1      ATGACAAAGCACCCAAGGAGGGTGCGATTGCTATTGTTGGTACCCTTGACGGCGTACCGG    1253
KU705815.1      ACGACAAAGCACCCAAGGAGGGTGCGATTGCTATTGTTGGTACCCTTGACGGCGTACCGG    1237
AF245004.1      ACGACAAAGCACCCAAGGAGGGTGCGATTGCTATTGTTGGCACCCTTGACGGCGTACCGG    1241
JF412263.1      ACGACAAAGCACCCAAGGAGGGTGCGATTGCTATTGTTGGTACCCTTGACGGCGTACCGG    1253
MN309752.1      ACGACAAAGCACCCAAGGAGGGTGCGATTGCTATTGTTGGTACCCTTGACGGCGTACCGG    1253
KM588181.1      ACGACAAAGCACCCAAGGAGGGTGCGATTGCTATTGTTGGTACCCTTGACGGCGTACCGG    1250
FJ789784.1      ACGACAAAGCACCCAAGGAGGGTGCGATTGCTATTGTTGGTACCCTTGACGGCGTACCGG    1253
AF245003.1      ATGACAAAGCACCCAAGGAGGGTGCGATTGCTATTGTTGGTACCCTTGACGGCGTACCGG    1241
NC_008041.1     ACGACAAAGCACCCAAGGAGGGTGCGATTGCTATTGTTGGTACCCTTGACGGCGTACCGG    1253
AY744705.1      ATGACAAAGCACCCAAGGAGGGTGCGATTGCTATTGTTGGTACCCTTGACGGCGTACCGG    1253
                * ******** ***** *****  * ************** *** **** ******** *

KY354702.2      GAACAGTTGACGGCGCTTACCTCGCCGAATCTGTCTAGCGTGCTTGATACGGTGCCAGCT    1289
KF386164.1      CTACGGTTGACGGCCTTTACACGGCCACACCCGTGCCGCGTGCCTAATAGGGTGCCAGCT    1314
JX402858.1      CTACGCTCGACGGCCTTTATACGGCTGGAGACGTGCCGCGTGCTTGATTGGGTGCCAGTG    1253
KY315689.1      CTACGCTTGAAGGCCTATACACGGCTTGAAGCGCGCCGCTTGTCTGTGCACAGGCTAAAT    1313
JN189992.2      CTACGCTCGAAGGCCTATATACGGCTGGAAGCGCGCCGCGTGCTTGATTGGGTGCCAGTG    1313
GU592791.1      CTACGCTTGAAGGCCTATACACGGCTTGAAGCGCGCCGCGTGCTTAATTGGGTGCCAGTG    1298
AF499774.1      CT-CGCTTGAAGGCCTATACACGGCTGGAAGCGCGCCGCGTGCTTAATTGGGTGCCAGTG    1286
MG637439.1      CTACGCTTGAAGGCCTATATACGGCTGGAAGCGCGCCGCGTGCTTGATTGGGTGCCAGTG    1313
KU705815.1      CTACGCTTGAAGGCCTATACACGGCTGGAAGCGCGCCGCGTGCTTAATTGGGTGCCAGTG    1297
AF245004.1      CCACGCTTGAAGGCCTATATACGGCTGGAAGCGCGCCGCGTGCTTAATTGGGTGCCAGTG    1301
JF412263.1      CTACGCTTGAAGGCCTATACACGGCTGGAAGCGCGCCGCGTGCTTAATTGGGTGCCAGTG    1313
MN309752.1      CTACGCTTGAAGGCCTATACACGGCTGGAAGCGCGCCGCGTGCTTAATTGGGTGCCAGTG    1313
KM588181.1      CTACGCTTGAAGGCCTATACACGGCTGGAAGCGCGCCGCGTGCTTAATTGGGTGCCAGTG    1310
FJ789784.1      CTACGCTTGAAGGCCTATACACGGCTGGAAGCGCGCCGCGTGCTTAATTGGGTGCCAGTG    1313
AF245003.1      CTACGCTTGAAGGCCTGTACACGGCTGGAAGCGCGCCGCGTGCTTAATTGGGTGCCAGTG    1301
NC_008041.1     CTACGCTTGAAGGCCTATACACGGCTGGAAGCGCGCCGCGTGCTTAATTGGGTGCCAGTG    1313
AY744705.1      CTACGCTTGAAGGCCTATACACGGCTGGAAGCGCGCCGCGTGCTTAACTGGGTGCCAGTG    1313
                   *  * ** ***   **    **   *   *    ** **  *        ** *

KY354702.2      TTACCAGTCTTGTCCAACGCCGAGGATTTCCCTCTTTGGGCATGTTGGGTTACCGTTAGC    1349
KF386164.1      TTACCAGTCGTATCCACGCCGAGGATTTCCCCCCTTGG-GCATGTTGGGTTACCGTCAGC    1373
JX402858.1      GTACCATTCGTATCCACCGCCGAGGATTTCCCTCTTTG-GGCTGTTGGGTTACCGTTAGC    1312
KY315689.1      GTCTGTACACAGGCTAAAACCAGTGTTCTCCAGCA-------TGTTCCGTGGTACATGCC    1366
JN189992.2      GTACCAGTCGTATTCAACGCCGATGAAATCCCTCTTTG-GGCTGTTGGGTTACCGTCAGC    1372
GU592791.1      GTACCAGTCGTATCCAACGCCGAGGAAGTCCCTCTTTG-GGCTGTTGGGTTACCGTTAGC    1357
AF499774.1      GTACCAGTCCGTATCCAACGCCGAGGAAGTCCTCTTTG-GGCTGTTGGGTTACCCGTAGC    1345
MG637439.1      GTACCAGTCGTATCCACG-CCGAGGAATTCCCTCTTTG-GGCTGTTGGGTTACCGTTAGC    1371
KU705815.1      GTACCAGTCGTATCCAACGCCGAGGAAATCCTCTGGCT-GGA------------------    1338
AF245004.1      GTACCAGTCGTATCCAACGCCGAGGAAGTCCCTCTTTG-GGCTGTTGGGTTACCGTTAGC    1360
JF412263.1      GTACCAGTCGTATCCAACGCCGAGGAACTCCCTCTTTG-GGCTGTTGGGTTACCGTTAGC    1372
MN309752.1      GTACCAGTCGTATCCAACGCCGAGGAATTCCCTCTTTG-GGCTGTTGGGTTACCGTTAGC    1372
KM588181.1      GTACCAGTCGTATCCAACGCCGAGGACATCCCTCTTTG-GGCTGTTGGGTTACCGTTAGC    1369
FJ789784.1      GTACCAGTCGTATCCAACGCCGAGGAAGTCCCTCTTTG-GGCTGTTGGGTTACCGTTAGC    1372
AF245003.1      GTACCAGTCGTATCCAACGCCGAGGAAGTCCCTCGTTG-GGCTGTTGGGTTACCGTTAGC    1360
NC_008041.1     GTACCAGTCGTATCCAACGCCGAGGAAGTCCCTCTTTG-GGCTGTTGGGTTACCGTTAGC    1372
AY744705.1      GTACCAGTCGTATCCAACGCCGAGGAAGTCCCTCTTTG-GGCTGTTGGGTTACCGTTAGC    1372
                 *                             *

KY354702.2      TCCGCGCAGTGAGCACCACCG------CCATGTGGTTAAATGGCCGCTGATCG-------    1396
KF386164.1      TCCACGTATCGAGCGCCACCGCCA------TGTGGCTAAATGGCCGCTGATCGCTACCCA    1427
JX402858.1      TCCGCGCAGTGAGCACCACCG---------------------------------------    1333
KY315689.1      AAGGCACACACCAGCCGAAATCGCTGACGCAGTGCAAAACTTGGCCATGCCAAGGCACAC    1426
JN189992.2      TCCGCGCA-----GTGAGCACCAC-CGCCATGTGGTTAAATGGCCGCTGATC-G------    1419
GU592791.1      TCCGCGTA-----GTGAGCACCAC-CGCCATGTGGTAAANNGGCCCCNGNCT-A------    1404
AF499774.1      TCCGCGCA-----GGGAGCACCAC-CGC--------------------------------    1367
MG637439.1      TCCGCGCA-----GTGAGCACCAC-CGCCATGTGGTTAAATGGCCGCTGATC-G------    1418
KU705815.1      ------------------------------------------------------------    1338
AF245004.1      TCCGCGCA-----GTGAGCACCAC-CGCCATGTGGT------------------------    1390
JF412263.1      TCCGCGCA-----GTGAGCACCAC-CGCCATGTGGTTAAATGGCCGCTGATC-G------    1419
MN309752.1      TCCGCGCA-----GTGAGCACCAC-CGCCATGTGGTTAAATGGCCGCTGATC-G------    1419
KM588181.1      TCCGCGCA-----GTGAGCACCAC-CGCCATGCGGTTAAATGGCCGCTGATC-G------    1416
FJ789784.1      TCCGCGCA-----GTGAGCACCAC-CGCCATGTGGTTAAATGGCCGCTGATC-G------    1419
AF245003.1      TCCGCGCA-----GTGAGCACCAC-CGCCATGTGGT------------------------    1390
NC_008041.1     TCCGCGTA-----GTGAGCACCAC-CGCCATGTGGTTAAATGGCCGCTGATC-G------    1419
AY744705.1      TCCGCGCA-----GTGAGCACCAC-CGCCATGTGGTTAAATGGCCGCTGATC-G------    1419
```

```
KY354702.2      ------------------------------------------------------------    1396
KF386164.1      TTTCGGC-----------------------------------------------------    1434
JX402858.1      ------------------------------------------------------------    1333
KY315689.1      ACCAGCCGAAATCGCTGACGCAGTGCAAAACTTGGCCGCAAGTTCCGCTGAGCTGGTTGA    1486
JN189992.2      --CTTCTCAACTCGGC--------------------------------------------    1433
GU592791.1      ------------------------------------------------------------    1404
AF499774.1      ------------------------------------------------------------    1367
MG637439.1      --CTTCTCAACTCGGC--------------------------------------------    1432
KU705815.1      ------------------------------------------------------------    1338
AF245004.1      ------------------------------------------------------------    1390
JF412263.1      --CTTCTCAACTCGGCG-------------------------------------------    1434
MN309752.1      --CTTCTCAACTCGGCG-------------------------------------------    1434
KM588181.1      --CTTCTCAACTCTGC--------------------------------------------    1430
FJ789784.1      --CTTCTCAACTCGGC--------------------------------------------    1433
AF245003.1      ------------------------------------------------------------    1390
NC_008041.1     --CTTCTCAACTCGGCG-------------------------------------------    1434
AY744705.1      --CTTCTCAACTGTGGTT------------------------------------------    1435

KY354702.2      ------------------------------------------------------------    1396
KF386164.1      ------------------------------------------------------------    1434
JX402858.1      ------------------------------------------------------------    1333
KY315689.1      AACTGACTGTGCTTAATTGGGTGCCAGTGGTACCAGTCGTATCCACCGTGCCAAGGCACA    1546
JN189992.2      ------------------------------------------------------------    1433
GU592791.1      ------------------------------------------------------------    1404
AF499774.1      ------------------------------------------------------------    1367
MG637439.1      ------------------------------------------------------------    1432
KU705815.1      ------------------------------------------------------------    1338
AF245004.1      ------------------------------------------------------------    1390
JF412263.1      ------------------------------------------------------------    1434
MN309752.1      ------------------------------------------------------------    1434
KM588181.1      ------------------------------------------------------------    1430
FJ789784.1      ------------------------------------------------------------    1433
AF245003.1      ------------------------------------------------------------    1390
NC_008041.1     ------------------------------------------------------------    1434
AY744705.1      ------------------------------------------------------------    1435

KY354702.2      ------------------------------------------------------------    1396
KF386164.1      ------------------------------------------------------------    1434
JX402858.1      ------------------------------------------------------------    1333
KY315689.1      CACCAGCCGAAATCGCTGACGCAGTGCAAAACTTGGCCATGCCAAGGCACACACCAGCCG    1606
JN189992.2      ------------------------------------------------------------    1433
GU592791.1      ------------------------------------------------------------    1404
AF499774.1      ------------------------------------------------------------    1367
MG637439.1      ------------------------------------------------------------    1432
KU705815.1      ------------------------------------------------------------    1338
AF245004.1      ------------------------------------------------------------    1390
JF412263.1      ------------------------------------------------------------    1434
MN309752.1      ------------------------------------------------------------    1434
KM588181.1      ------------------------------------------------------------    1430
FJ789784.1      ------------------------------------------------------------    1433
AF245003.1      ------------------------------------------------------------    1390
NC_008041.1     ------------------------------------------------------------    1434
AY744705.1      ------------------------------------------------------------    1435

KY354702.2      ------------------------------------------------------------    1396
KF386164.1      ------------------------------------------------------------    1434
JX402858.1      ------------------------------------------------------------    1333
KY315689.1      AAATCGCTGACGCAGTGCAAAACTTGGCCGCAACCGAGGAGTCCCTCTTTGGGCTGTTGG    1666
JN189992.2      ------------------------------------------------------------    1433
GU592791.1      ------------------------------------------------------------    1404
AF499774.1      ------------------------------------------------------------    1367
MG637439.1      ------------------------------------------------------------    1432
KU705815.1      ------------------------------------------------------------    1338
AF245004.1      ------------------------------------------------------------    1390
JF412263.1      ------------------------------------------------------------    1434
MN309752.1      ------------------------------------------------------------    1434
KM588181.1      ------------------------------------------------------------    1430
FJ789784.1      ------------------------------------------------------------    1433
AF245003.1      ------------------------------------------------------------    1390
NC_008041.1     ------------------------------------------------------------    1434
AY744705.1      ------------------------------------------------------------    1435

KY354702.2      ------------------------------------------------------------    1396
```

```
KF386164.1      ------------------------------------------------------------    1434
JX402858.1      ------------------------------------------------------------    1333
KY315689.1      ATTACCGTTAGCTCCGCGCAGTGAGCACCACCGCCATGTGGTTAAATGGCCACTGATCGC    1726
JN189992.2      ------------------------------------------------------------    1433
GU592791.1      ------------------------------------------------------------    1404
AF499774.1      ------------------------------------------------------------    1367
MG637439.1      ------------------------------------------------------------    1432
KU705815.1      ------------------------------------------------------------    1338
AF245004.1      ------------------------------------------------------------    1390
JF412263.1      ------------------------------------------------------------    1434
MN309752.1      ------------------------------------------------------------    1434
KM588181.1      ------------------------------------------------------------    1430
FJ789784.1      ------------------------------------------------------------    1433
AF245003.1      ------------------------------------------------------------    1390
NC_008041.1     ------------------------------------------------------------    1434
AY744705.1      ------------------------------------------------------------    1435

KY354702.2      -------------       1396
KF386164.1      -------------       1434
JX402858.1      -------------       1333
KY315689.1      TTTTCTACTCGGC       1739
JN189992.2      -------------       1433
GU592791.1      -------------       1404
AF499774.1      -------------       1367
MG637439.1      -------------       1432
KU705815.1      -------------       1338
AF245004.1      -------------       1390
JF412263.1      -------------       1434
MN309752.1      -------------       1434
KM588181.1      -------------       1430
FJ789784.1      -------------       1433
AF245003.1      -------------       1390
NC_008041.1     -------------       1434
AY744705.1      -------------       1435
```

Figure S3. Sequences of the RNA1 and RNA2 fragments of the NNV isolate from this study.

```
RNA1 fragment amplified in this study                          bp
GATGAGTTCACTATGATGGTCAAAGCGTGTTCAAAGAAAGAAGCATACAACGCCCCAAAT    60
TACCCCAGGAACATTTCAACCGTTCCGCACACCCAAAACGTCAAGTTGTCCAGCTACACC   120
TACGCTTTCAAAGCCAGTGTTCTCCAGCATGTTCCGTGGTACATGCCAACGCACACCCCA   180
GCGGAAATCGCTGACGCAGTGCAAAACTTGGCCGCAAGTTCCACTGAGCTGGTTGAAACT   240
GACTACAGCAAGTTCGATGGCACATTCTTGCGCTTTATGCGTGAGTGCGTCGAATTTGCC   300
ATCTATAAGCGCTGGGTTCACCTGGACCACTTGCCAGAGTTAACAACCTTATTGGCTAAT   360
GAGATCCAAGCACCTGCTGTTACACGACTGGGCATCAAGTATGATCCTGATTGCAGTCGC   420
CTCAGTGGTTCTGCTCTCACGACAGACGGAAACAGCATTGCTAATGCGTTCGTCTCATAC   480
CTTGCTGGTCGCATGGCTGGCATGGATGATGATGAAGCTTGGTCTTGGATCGGCATTGTC   540
TACGGTGATGATGGGCTCCGATCTGGTAATGTTTCAAATGAGCTCCTCACCAACACTGCT   600
TCTTCCCTCGGCTTTGACTTGAAAATAGTGAATCGCGCGCCACGTGGCTCTCCAGTGACA   660
TTTCTGTCTCGAGTATACCTCGATCCTTGGTCCTCACCGGCTTCCGTGCAGTCGCCATTA   720
AGAACATTATTGAAATTGCACACCACCTGTGATACCCAGTCAGAGATTGACGACATTG     778
```

```
RNA2 fragment amplified in this study                          bp
CCAAGGCCCGCGAATCCGCAACCTCGTCGACGTGCTAACAATCGTCGGCGTAGTAATCGC    60
ACTGACGCACCTGTGTCCAAGGCCTCGACCGTAACTGGATTTGGACGTGGGACCAATGAC   120
GTCCATCTCTCAGGTATGTCGAGAATCTCCCAGGCCGTCCTCCCAGCCGGGACAGGAACA   180
GACGGATACGTTGTTGTTGACGCAACCATCGTCCCCGACCTCCTGCCACGACTGGGACAC   240
GCTGCTAGAATCTTCCAGCGATACGCTGTTGAAACACTGGAGTTTGAAATTCAGCCAATG   300
TGCCCCGCAAACACGGGCGGTGGTTACGTTGCTGGCTTCCTGCCTGATCCAACTGACAAC   360
GATCACACCTTCGACGCACTTCAAGCAACTCGTGGTGCAGTCGTTGCCAAATGGTGGGAA   420
AGCAGAACAGTCCGACCGCAGTTCACTCGCACGCTCCTCTGGACCTCGTCGGGAAAGGAG   480
CAGCGTCTTACATCACCTGGTCGGCTGATACTCCTGTGTGTCGGCAACAACACTGACGTG   540
GTTAACGTGTCGGTGCTGTGTCGCTGGAGTGTTCGACTGAGCGTTCCGTCTCTTGAAACA   600
CCTGAAGAGACCACCGCTCCCATCATGACACAAGGTCCCCTGTACAATGATTCCCTCGCT   660
GCAACTGACTTCAAGTCCATCCTCCTAGGATCTACAGCACTGGACATTGCTCCTGACGGA   720
GCAATCTTCCAGCTGGACCGTCCGTTGTCCATTGACTACAACCTTGGAGTTGGAGATGTT   780
GACCGTGCCGTCTACTGGCACCTCAAGAAGTTTGCTGGCACCGCTGCCACACCTGCAGGC   840
TGGTTTCGCTGGGGCATCTGGGACAACTTCAACAAAACGTTCACAGATGGCGTTGCTTAC   900
TACTCCGATCAGCAGCCTCGTCAAATCCTGCTGCCTGTTGGCACTGTCTGCACCAGGGGT   960
TGACTCGGAAAACTAACCGGGTCATCC                                    987
```

References

1. Okinaka, Y.; Nakai, T. Comparisons among the complete genomes of four betanodavirus genotypes. *Diseases of aquatic organisms* **2008**, *80*, 113-121.
2. Iwamoto, T.; Okinaka, Y.; Mise, K.; Mori, K.-I.; Arimoto, M.; Okuno, T.; Nakai, T. Identification of host-specificity determinants in betanodaviruses by using reassortants between striped jack nervous necrosis virus and sevenband grouper nervous necrosis virus. *Journal of virology* **2004**, *78*, 1256-1262.
3. Iwamoto, T.; Mise, K.; Mori, K.-i.; Arimoto, M.; Nakai, T.; Okuno, T. Establishment of an infectious rna transcription system for striped jack nervous necrosis virus, the type species of the betanodavirusesthe ddbj accession nos of the sequences reported in this paper are ab056571 and ab056572. *Journal of General Virology* **2001**, *82*, 2653-2662.
4. Vendramin, N.; Toffan, A.; Mancin, M.; Cappellozza, E.; Panzarin, V.; Bovo, G.; Cattoli, G.; Capua, I.; Terregino, C. Comparative pathogenicity study of ten different betanodavirus strains in experimentally infected e uropean sea bass, d icentrarchus labrax (l.). *Journal of Fish Diseases* **2014**, *37*, 371-383.
5. Panzarin, V.; Fusaro, A.; Monne, I.; Cappellozza, E.; Patarnello, P.; Bovo, G.; Capua, I.; Holmes, E.C.; Cattoli, G. Molecular epidemiology and evolutionary dynamics of betanodavirus in southern europe. *Infection, genetics and evolution* **2012**, *12*, 63-70.
6. Chérif, N.; Gagne, N.; Groman, D.; Kibenge, F.; Iwamoto, T.; Yason, C.; Hammami, S. Complete sequencing of tunisian redspotted grouper nervous necrosis virus betanodavirus capsid gene and rna-dependent rna polymerase gene. *Journal of fish diseases* **2010**, *33*, 231-240.
7. Tan, C.; Huang, B.; Chang, S.F.; Ngoh, G.H.; Munday, B.; Chen, S.C.; Kwang, J. Determination of the complete nucleotide sequences of rna1 and rna2 from greasy grouper (epinephelus tauvina) nervous necrosis virus, singapore strainthe sequences reported in this paper have been deposited in genbank under the accession nos af318942 and af319555. *Journal of General Virology* **2001**, *82*, 647-653.
8. Chen, X.; Weng, S.; Chen, S.; Chen, Z.; He, J. Protective immunity of orange-spotted grouper (epinephelus coioids) against a nervous necrosis virus isolated from china, and determination of the complete sequences of the virus. *ACTA OCEANOLOGICA SINICA-ENGLISH EDITION-* **2007**, *26*, 101.
9. Liu, H.; Teng, Y.; Zheng, X.; Wu, Y.; Xie, X.; He, J.; Ye, Y.; Wu, Z. Complete sequence of a viral nervous necrosis virus (nnv) isolated from red-spotted grouper (epinephelus akaara) in china. *Archives of virology* **2012**, *157*, 777-782.
10. Jia, P.; Jia, K.-T.; Yi, M.-S. Complete genome sequence of a fish nervous necrosis virus isolated from sea perch (lateolabrax japonicus) in china. *Genome announcements* **2015**, *3*.
11. Qin, Y.; Liu, J.; Liu, W.; Shi, H.; Jia, A.; Lu, Y.; Liu, X. First isolation and identification of red-grouper nervous necrosis virus (rgnnv) from adult hybrid hulong grouper (epinephelus fuscoguttatus× epinephelus lanceolatus) in china. *Aquaculture* **2020**, *529*, 735662.
12. Xing, J.; Zhang, Z.; Sheng, X.; Tang, X.; Chi, H.; Zhan, W. Identification and characterization of a new strain of nervous necrosis virus isolated from pearl gentian grouper (epinephelus lanceolatus× epinephelus fuscoguttatus) in china. *Aquaculture* **2020**, *529*, 735663.
13. Sommerset, I.; Nerland, A.H. Complete sequence of rna1 and subgenomic rna3 of atlantic halibut nodavirus (ahnv). *Diseases of aquatic organisms* **2004**, *58*, 117-125.
14. Chien, M.-H.; Vo, T.T.M.; Wu, S.-Y.; Lin, C.-H. Complete genome sequence of nervous necrosis virus isolated from orange-spotted grouper (epinephelus coioides) in taiwan. *Genome announcements* **2017**, *5*.
15. Toffan, A.; Pascoli, F.; Pretto, T.; Panzarin, V.; Abbadi, M.; Buratin, A.; Quartesan, R.; Gijón, D.; Padrós, F. Viral nervous necrosis in gilthead sea bream (sparus aurata) caused by reassortant betanodavirus rgnnv/sjnnv: An emerging threat for mediterranean aquaculture. *Scientific reports* **2017**, *7*, 1-12.
16. Toffan, A.; De Salvador, M.; Scholz, F.; Pretto, T.; Buratin, A.; Rodger, H.D.; Toson, M.; Cuenca, A.; Vendramin, N. Lumpfish (cyclopterus lumpus, linnaeus) is susceptible to viral nervous necrosis: Result of an experimental infection with different genotypes of betanodavirus. *Journal of fish diseases* **2019**, *42*, 1667-1676.
17. Huang, J.; Lin, L.; Weng, S.; He, J. High expression of capsid protein of red-spotted grouper nervous necrosis virus in an avian cell line requires viral rna2 non-coding regions. *Journal of fish diseases* **2007**, *30*, 439-444.
18. Chen, C.-W.; Wu, M.-S.; Huang, Y.-J.; Cheng, C.-A.; Chang, C.-Y. Recognition of linear b-cell epitope of betanodavirus coat protein by rg-m18 neutralizing mab inhibits giant grouper nervous necrosis virus (ggnnv) infection. *PLoS One* **2015**, *10*, e0126121.
19. Toffan, A.; Biasini, L.; Pretto, T.; Abbadi, M.; Buratin, A.; Franch, R.; Dalla Rovere, G.; Panzarin, V.; Marsella, A.; Bargelloni, L. Age dependency of rgnnv/sjnnv viral encephalo-retinopathy in gilthead sea bream (sparus aurata). *Aquaculture* **2021**, *539*, 736605.
20. Lin, C.-S.; Lu, M.-W.; Tang, L.; Liu, W.; Chao, C.-B.; Lin, C.J.; Krishna, N.K.; Johnson, J.E.; Schneemann, A. Characterization of virus-like particles assembled in a recombinant baculovirus system expressing the capsid protein of a fish nodavirus. *Virology* **2001**, *290*, 50-58.
21. Mao, M.-G.; Wen, S.-H.; Perálvarez-Marín, A.; Li, H.; Jiang, J.-L.; Jiang, Z.-Q.; Li, X.; Sun, H.; Lü, H.-Q. Evidence for and characterization of nervous necrosis virus infection in pacific cod (gadus macrocephalus). *Archives of virology* **2015**, *160*, 2237-2248.
22. Hegde, A.; Teh, H.; Lam, T.; Sin, Y. Nodavirus infection in freshwater ornamental fish, guppy, poicelia reticulata–comparative characterization and pathogenicity studies. *Archives of virology* **2003**, *148*, 575-586.
23. Knibb, W.; Luu, G.; Premachandra, H.; Lu, M.-W.; Nguyen, N.H. Regional genetic diversity for nnv grouper viruses across the indo-asian region–implications for selecting virus resistance in farmed groupers. *Scientific reports* **2017**, *7*, 1-11.
24. Keawcharoen, J.; Techangamsuwan, S.; Ponpornpisit, A.; Lombardini, E.; Patchimasiri, T.; Pirarat, N. Genetic characterization of a betanodavirus isolated from a clinical disease outbreak in farm-raised tilapia oreochromis niloticus (l.) in thailand. *Journal of Fish Diseases* **2015**, *38*, 49-54.
